# Synthesis of diarylmethanes by means of Negishi cross-coupling enabled by cobalt-solvent coordination

**DOI:** 10.1038/s41598-025-10180-1

**Published:** 2025-07-23

**Authors:** Jakub Robaszkiewicz, Wojciech Chaładaj, Piotr Pawluć, Maciej Zaranek

**Affiliations:** 1 Center for Advanced Technologies, Adam Mickiewicz University, Uniwersytetu Poznańskiego 10, Poznań, 61-614 Poland; 2 Faculty of Chemistry, Adam Mickiewicz University, Uniwersytetu Poznańskiego 8, Poznań, 61-614 Poland; 3 Institute of Organic Chemistry, Polish Academy of Sciences, Kasprzaka 44/52, Warszawa, 01-224 Poland

**Keywords:** Homogeneous catalysis, Reaction mechanisms, Cross-coupling reactions

## Abstract

The diarylmethane backbone is a base of molecules relevant for various industrial and especially pharmaceutical applications where they serve as a platform for the discovery of new drugs. One of the most versatile methods of their synthesis are cross-coupling reactions that have served synthetic chemistry for nearly five decades and offer diverse opportunities for the synthesis of complex organic compounds. Although palladium catalysts have traditionally dominated this field, the emergence of first-row transition-metal (pre)catalysts presents promising alternatives because of their cost-effectiveness and the possibility of new reactivities. In this context, Negishi cross-coupling of benzylzinc bromide and its congeners with aryl, alkyl, alkenyl, alkynyl iodides and bromides, facilitated by simple cobalt halides without the addition of an auxiliary ligand, is presented. The optimisation of reaction conditions, including solvent selection, is discussed alongside the mechanistic insights gained through computational studies. A variety of diarylmethanes were synthesised under mild conditions, with yields comparable to those obtained with Pd catalysts, with excellent selectivity (> 99%) provided by commercially available anhydrous CoBr_2_ / DMAc catalytic system.

**Supplementary Information:**

The online version contains supplementary material available at 10.1038/s41598-025-10180-1.

## Introduction

Diarylmethanes are useful platforms that can be easily modified by benzylic C-H functionalisation to give a variety of molecules of unique importance in pharmaceutical, agrochemical, and material sciences^1^. Their synthesis is a key step in the synthesis of various drugs, including antihistamines such as benadryl^2^, anticancer agents such as piritrexim^3^, vasodilators such as segontin^4^, and antidiabetics such as dapagliflozin, a sodium-glucose co-transporter 2 (SGLT-2) inhibitor^5^. Due to this breadth of application, various methods of constructing diarylmethane backbone have been devised. The most handbook ones are different variations on Friedel-Crafts alkylation or Friedel-Crafts acylation / reduction protocols^6–12^. Methods aimed at diarylmethanes also cover classical Pd-catalysed Suzuki-Miyaura and related cross-couplings^13–16^, organocatalysed^7^ and radical reactions^17–21^, as well as iron-catalysed Kumada cross-coupling^22^ and cobalt-catalysed reductive coupling^23,24^. The report on the last of these describes also conditions for Negishi cross-coupling, although it was not the goal of that research.

Cross-coupling reactions with organometallic reagents have been established as synthetic tools for nearly 50 years^25–27^. Wide scope of reagents and great number of reaction conditions to choose from made these processes successful despite the frequent need for expensive palladium catalysts. Negishi reaction is one such example which uses organozinc reagents whose great advantage is the relative stability and low nucleophilicity compared to organolithium and organomagnesium reagents (used in Murahashi and Kumada reactions, respectively)^28,29^ while maintaining the ability for fast transmetallation, contrary to organoboron and organosilicon compounds (used in Suzuki-Miyaura and Hiyama reactions). Furthermore, organozinc reagents are considerably less toxic than equivalent organotin compounds still used in Stille cross-coupling^30^.

Even though the field of cross-couplings can be considered mature, it is still a realm of intense research, especially considering the trend to abandon using platinum-group-metal catalysts. Cobalt catalysis has emerged as a promising alternative, mainly due to its reactivity patterns, some of which are unique to this metal^31–33^. Although studied extensively, new base metal catalytic systems for cross-coupling reactions are still sought after, especially those that would combine mild reaction conditions with stability and simple structure of (pre)catalysts.

**Fig. 1:**
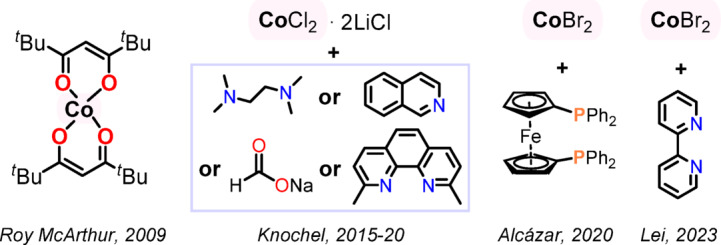
Representative cobalt-catalysed Negishi reaction systems^34–40^.

Figure 1 shows representative examples of such base-metal catalytic systems published over the past two decades. The first report on the cobalt-catalysed Negishi reaction dates back to 2009^34^. Roy MacArthur et al. described Co(II) acetylacetonato derivative complexes capable of catalysing cross-coupling between (*E*)-1-iodo-1-octene and butylzinc iodide to form (*E*)‑5‑dodecene. In 2015, diarylzinc reagents were the subject of a report by Knochel et al., in which they described the CoCl_2_ / 2LiCl (20 mol%) + *N*,*N*,*N’*,*N’‑*tetramethylethanediamine (30 mol%) catalytic system whose use provided cross-coupling products with primary and secondary alkyl iodides^36^. By further development of their catalytic system, the Knochel group has made a significant contribution to advance Co-catalysed Negishi cross-couplings^35,37,39^. Further, Dorval et al. showed Negishi-type cross coupling of glutaramides with organozinc reagents enabled by 20 mol% of cobalt(II) bromide in 1,4-dioxane^41^. The authors observed that polar aprotic solvents were incompatible with their conditions. Recently, Cossy et al. have proposed and described a mechanism of cobalt-catalysed Negishi cross-coupling of arylzinc reagents with (hetero)aryl halides catalysed by CoCl_2_(bpy)_2_ system that fundamentally relied on Co(I) generation through solvent-dependent disproportionation of Co(II)^40^. The formation of highly active Co(I) species and their stabilization by acetonitrile have been verified based on X-ray absorption fine structure spectroscopy allied with DFT calculations. This CoCl_2_/bipyridine/acetonitrile system has been extended to the Negishi acylation and allylation. Most recently, Lu and colleagues performed an enantioselective transformation of α-bromoketones by using cobalt iodide (10 mol%) and chiral unsymmetrical *N*,*N*,*N*‑tridentate ligand^42^. Common to all the reports presented above is that the addition of auxiliary ligands or activation was required.

It appears that in the literature there is no example of effective synthesis of diarylmethanes using Negishi cross coupling in a catalytic system comprised of a cobalt(II) (pre)catalyst without additives. Here, we report Negishi cross-coupling of benzylzinc bromide and its congeners with organic iodides and bromides catalysed by CoBr_2_ / solvent system, which turned out to be useful method of the synthesis of diarylmethanes. DFT studies were undertaken to shed light on the workings of the process described.

## Results and discussion

### Experimental research

**Fig. 2:**
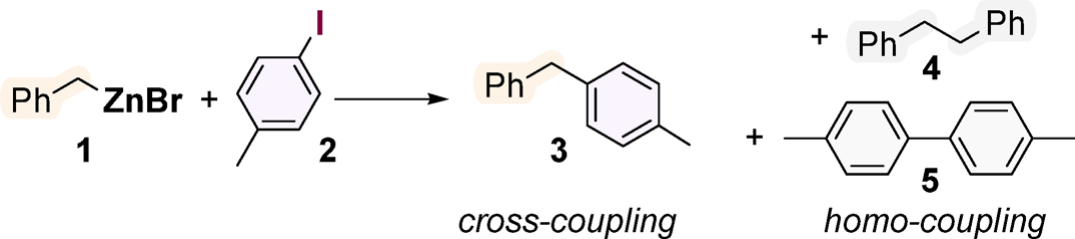
The model Negishi reaction system.

The research began with cross-coupling experiments between phenylzinc bromide and 4-iodotoluene in tetrahydrofuran (THF) catalysed by cobalt(II) complexes (see SI). These preliminary results indicated that, although possible, such a reaction was very nonselective, yielding the desired 4-methylbiphenyl in yields lower than 40% with other products resulting from homocoupling of the starting reactants. Curious as to whether the low selectivity is due to the influence of an aromatic Negishi reagent or whether it is an inherent property of the catalytic systems used, further experiments were conducted with benzylzinc bromide **1** as a coupling partner for 4-iodotoluene **2** (Fig. 2). Table 1 summarises the screening of various cobalt coordination complexes as potential (pre)catalysts of such Negishi cross-coupling.

**Table 1:** Screening of cobalt(II) complexes as potential (pre)catalysts.

#	Catalyst	Conv. of 2 ^b)^	Selectivity ^b)^		
			3	4	Other ^c)^
1	[CoBr_2_(PPh_3_)_2_]	70	38	38	24
2	[CoCl_2_(PPh_3_)_2_]	41	30	44	26
3	[CoBr_2_(2-Me-py)_2_]	53	27	44	29
4	[CoBr_2_(dppe)]	56	37	45	18
5	CoCl_2_	42	65	18	17
6	CoBr_2_	79	81	7	12
7	[CoCl_2_(2-Me-py)_2_]	57	17	48	35
8	[CoCl_2_(bipicoline)]	69	28	45	27
9	none	0	-	-	-

^(a)^ Conditions: 0.4 M in THF, 40 °C, 20 h [BnZnBr]: [4-MeC_6_H_4_I]: [Co] = 2 : 1 : 0.05; ^(b)^ determined by GC-MS; ^(c)^ **5** and toluene as product of possible demetalation of **1** and/or dehalogenation of **2**.

All cobalt compounds presented above turned out to catalyse the Negishi cross-coupling of benzylzinc bromide with 4-iodotoluene. Unexpectedly, the highest conversion of 4-iodotoluene was delivered by the simple, commercially available anhydrous cobalt(II) bromide (79%, entry 6), followed by two unrelated complexes (entries 1 and 8, 70% and 69%, respectively). It should be noted that reactions with simple Co halides (entries 5–6) were visibly more selective than those catalysed by other Co species. Having chosen the precatalyst, the next logical step was to determine the influence of the solvent, especially as a means of potentially increasing the yield of **3**. These experiments are summarised in Table 2.

**Table 2:** Screening of solvents for the negishi cross-coupling. Conditions: 1 M, 80 °C, 20 h, [BnZnBr]: [4-MeC_6_H_4_I]: [CoBr_2_] = 2 : 1 : 0.05;


#	Solvent	Conv. of **2** [%]^a)^	Selectivity of **3a** [%]^a)^
1	THF	90	91
2	1,4-dioxane	86	90
3	Eucalyptol	61	90
4	DMAc	> 99	> 99
5	DMF	76	88
6	Acetonitrile	81	92
7	NMP	87	92

^a^ determined by GC-MS. THF – tetrahydrofuran, DMAc – *N*,*N*-dimethylacetamide, DMF - *N*,*N-*dimethylformamide, NMP – *N*-methyl-2-pyrrolidone.

**Fig. 3:**
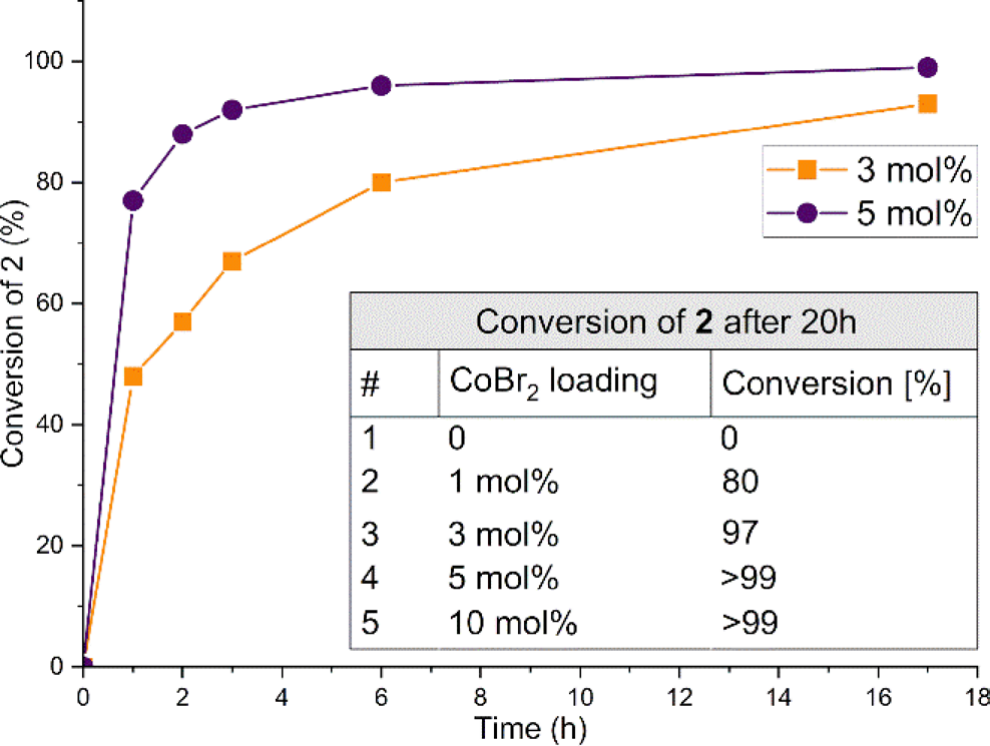
Screening of CoBr_2_ loading. Conditions: 1 M in DMAc, 80 °C, [BnZnBr]: [4-MeC_6_H_4_I] = 2 : 1; conversion determined by GC-MS.

As demonstrated by the results in the table, a variety of solvents, both very well known in the Negishi reaction and noncanonical ones, were reaction media appropriate for carrying out the model reaction. The least successful turned out to be eucalyptol (61%; entry3), an ether considered a green alternative to other ether solvents, such as tetrahydrofuran (THF) and 1,4-dioxane. The use of both of the latter led to conversions of 90% and 86% (entries 1 and 2, respectively), but the highest yield of **3a** with excellent selectivity (> 99%) was provided by *N*,*N-*dimethylacetamide (DMAc, entry 4) and it was the solvent of choice for the last step of optimisation of reaction conditions, i.e., screening of catalyst loading, whose results are presented in Fig. 3 and the inset table. The collected data suggested that for future complete conversion of more demanding aryl iodides, 5 mol% of CoBr_2_ and a reaction time longer than 8 h should be applied.

Concluding, the screening has shown that 5 mol% of CoBr_2_ without an additive is enough to achieve full conversion of 4-iodotoluene over about 6 h at 80 ºC.

During considerations regarding the role of solvent in this transformation, the evaporation of THF from the solution of benzylzinc bromide prior to the addition of DMAc was tried. Decreasing the amount of THF allowed for a higher conversion of **2** (see SI), and this information together with the data from Tables 1 and 2 led us to the conclusion that only a small amount of solvent (*N*,*N-*dimethylacetamide) is required. In fact, it was possible to perform a reaction of 1 mmol of **2** in only 0.2 ml of DMAc without THF which enabled full conversion of aryl iodides at the room temperature with the same selectivity.

**Fig. 4:**
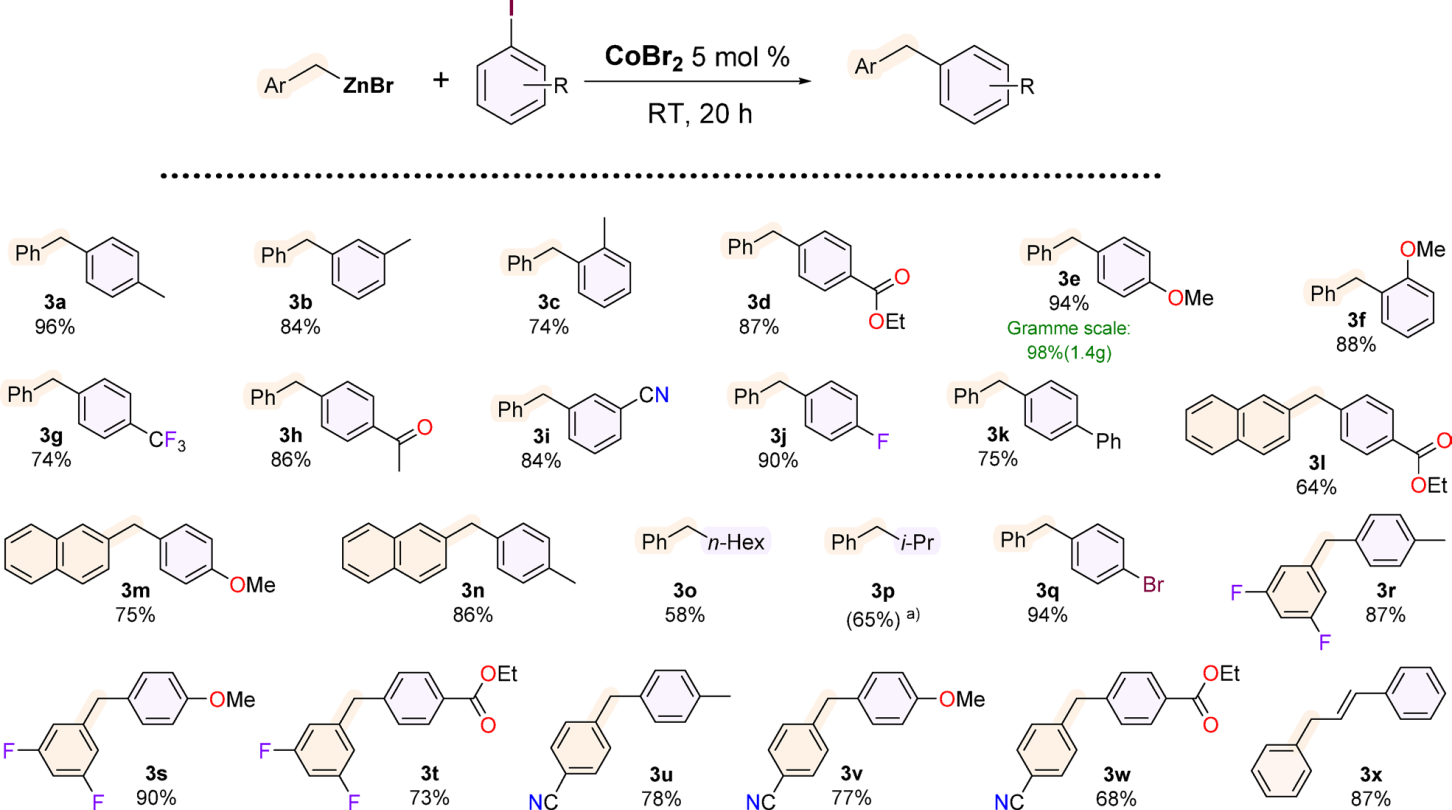
Products of CoBr_2_/DMAc-catalysed Negishi cross-coupling of aryl and alkyl iodides. Reaction conditions: ArCH_2_ZnBr (4 mmol), aryl/alkyl iodide (2 mmol), CoBr_2_ (0.1 mmol, 5 mol%), 0.4 mL DMAc, RT, inert atmosphere, 20 h. All values are isolated yields; ^b)^ Not isolated – GC yield given.

Having optimised the reaction conditions, we proceeded to examine the substrate scope of the proposed catalytic system (Fig. 4). Aryl iodides with groups such as methyl, methoxy, acyl, trifluoromethyl, nitrile, nitro, and amine were examined. Surprisingly, in reactions with alkyl iodides such as 1-iodohexane (**3o**), 2-iodopropane (**3p**), complete conversion of substrates was obtained as well as it was in the reaction of (*E*)‑β‑iodostyrene (**3x**) We observed that, most probably due to coordination properties, nitro and especially the amino groups were not suitable reagents for cross-coupling in this particular reaction system. A low conversion of these iodobenzene derivatives was recorded, but also many unidentified byproducts were observed. However, it is worth noting that a gramme-scale Negishi reaction between benzylzinc bromide and 4-iodoanisole resulted in isolating product **3e** with a 98% yield. It is also significant that the reaction with *p*-bromoiodobenzene at room temperature led to the sole substitution of the iodine atom, leaving the bromine unreacted (3q), which opens the possibility for further functionalisation.

Knowing the importance and greater accessibility of aryl bromides, we attempted to modify the reaction conditions to include this class of reagents. It turned out that a modification of the conditions by increasing the temperature and CoBr_2_ loading allowed us to carry out cross-coupling of these as far unreactive reagents.

**Fig. 5:**
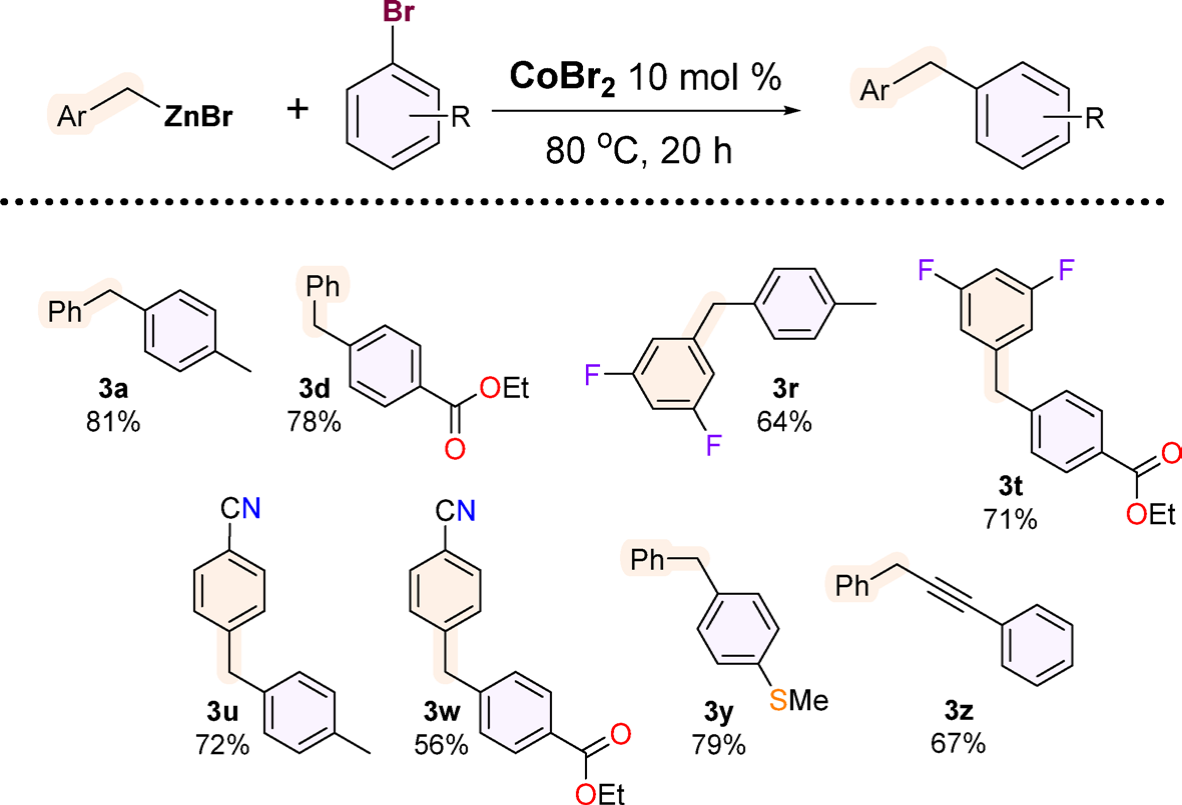
Products of CoBr_2_/DMAc-catalysed Negishi cross-coupling of aryl bromides. Reaction conditions: ArCH_2_ZnBr (2 mmol), aryl bromide (1 mmol), CoBr_2_ (0.1 mmol, 10 mol%), 0.4 mL DMAc, 80 °C, inert atmosphere, 20 h. All values are isolated yields.

The results of the Negishi benzylation of selected aryl bromides are presented in Fig. 5. The cross-coupling reaction carried out at a higher temperature was accompanied by the formation of slightly higher amounts of 1,2-diphenylethane compared to the reactions with aryl iodides.

Regarding the variability of the organozinc coupling partner, 2-naphthylmethylzinc bromide was also successfully transformed, as well as 3,5-difluorobenzylzinc bromide and 4-cyanobenzylzinc bromide. A couple of common products show a comparison in the reactivity of organic bromides and iodides, where the latter turned out to be significantly easier to convert.

**Fig. 6:**
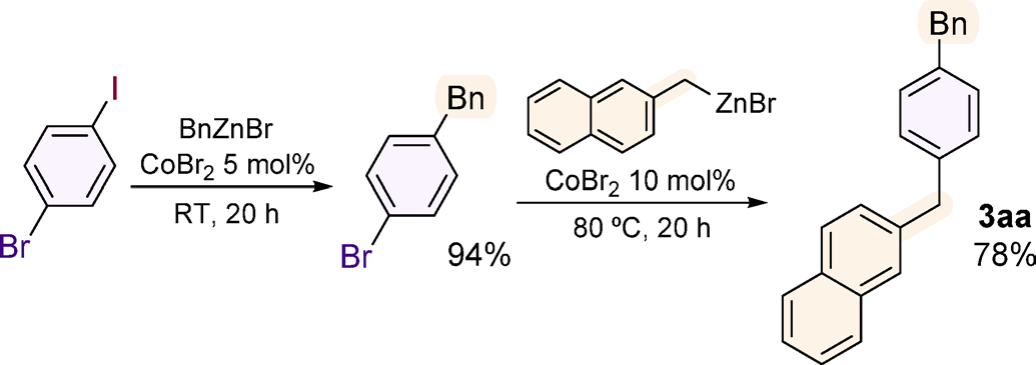
Two-step chemoselective cross-coupling of 1‑bromo‑4‑iodobenzene.

Distinct conditions required to carry out reactions with aryl iodides vs. aryl bromides were a premise that a sequential chemoselective cross-coupling of these moieties might be possible, which was demonstrated using 1‑bromo‑4‑iodobenzene (Fig. 6).

Furthermore, to ensure that our system was homogeneous, we decided to use scanning electron microscopy (SEM) to analyse the sample of solid residue separated from the reaction mixture. Energy-dispersive spectroscopy (SEM-EDS) showed no presence of cobalt, which allowed us to conclude about the absence of catalytic metal nanoparticles.

### Theoretical modelling

**Fig. 7:**
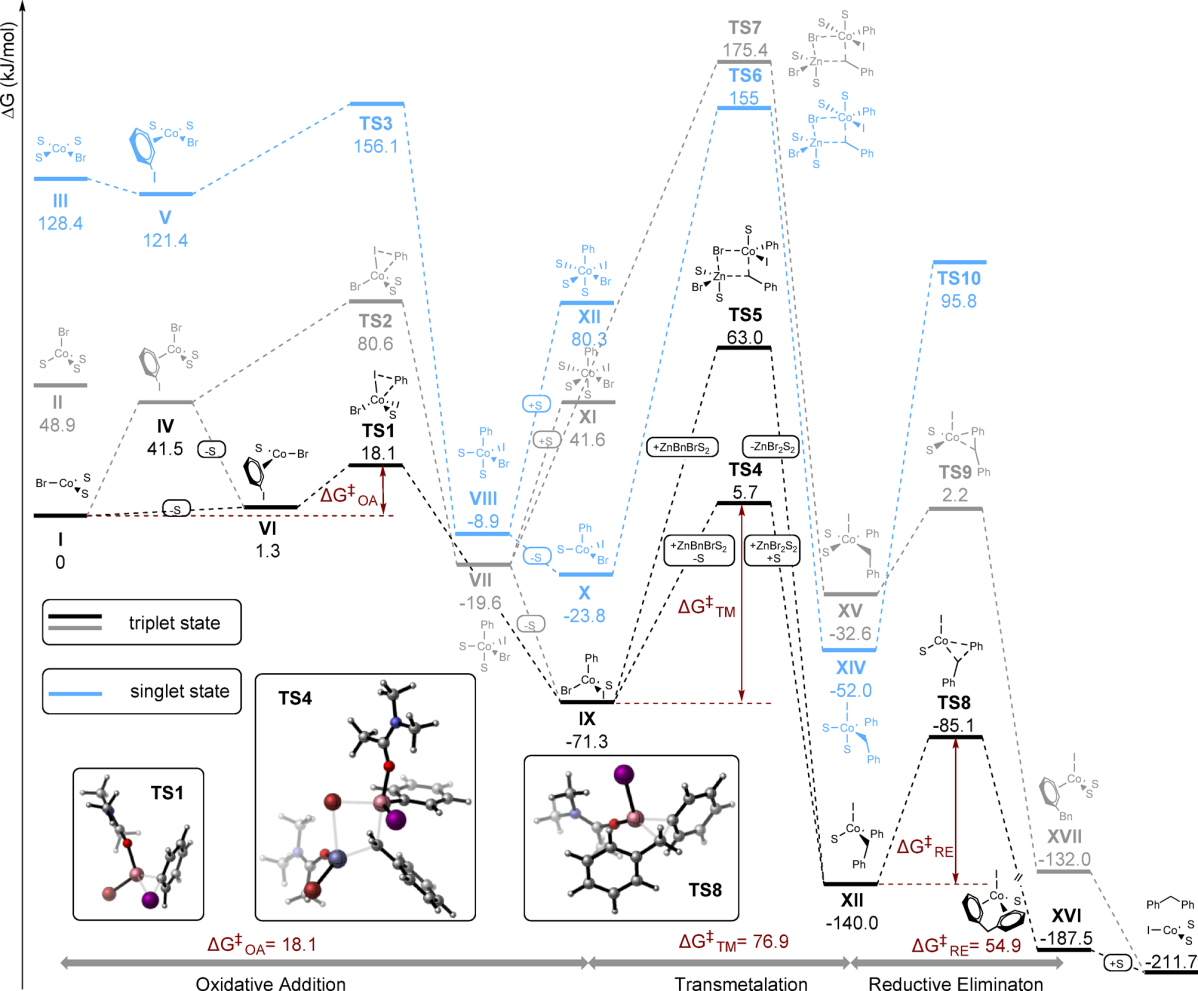
Gibbs Free Energy profiles calculated for possible reaction pathways starting from either a triplet (black and grey lines) or a singlet (blue lines) state.

To gain insight into the mechanistic details, the reaction was investigated computationally (Fig. 7). Reaction paths in both singlet and triplet states were considered. Generally, the triplet pathway (black and grey) is strongly preferred over the singlet pathway (blue), which is consistent with the reported models for cross-couplings catalysed by Co complexes (typically with N-ligands)^40^. Furthermore, complexes with a variety of coordinated solvent molecules were investigated.

**Fig. 8:**
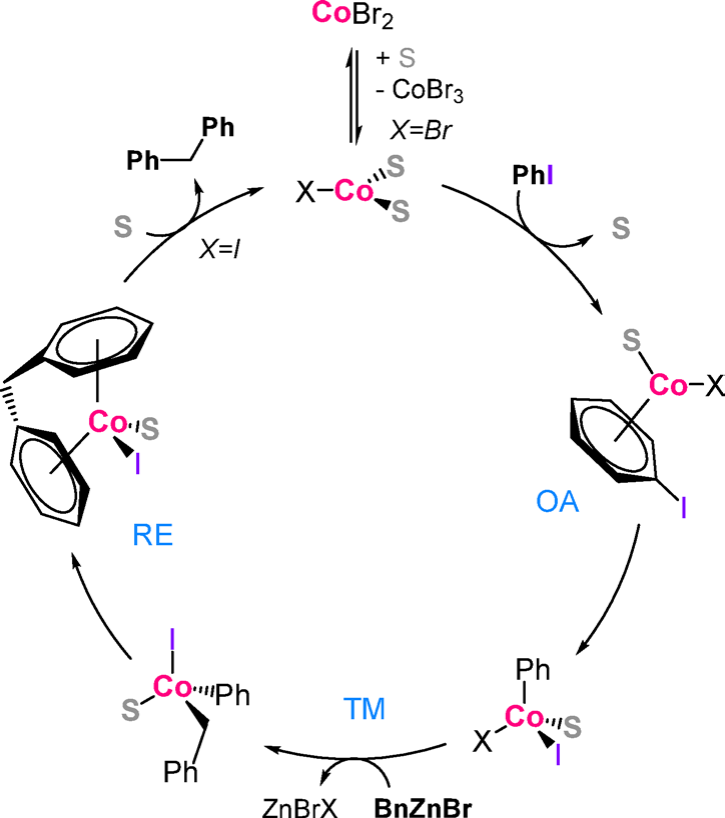
Catalytic cycle of Negishi cross-coupling in CoBr_2_ / DMAc catalytic system.

The initial generation of Co(I) species has already been extensively described^40^. Triplet Co(I) complexes prefer flat trigonal over tetrahedral geometries (cf. **I** with **II** and **IV** with **VI**), also contrasting with singlet square planar arrangements (**III** and **V**). In contrast, Co(III) intermediates adopt only slightly distorted tetrahedral geometries favoured over trigonal bipyramidal and octahedral complexes bearing more solvent molecules (cf. **IX** with **VII** and **XI** and **XII** with **XV**).Therefore, the most probable pathway (black) involves facile oxidative addition (**TS1**) followed by rate-limiting transmetalation (**TS 4**) and reductive elimination (**TS8**). Transmetalation occurs through a four-membered transition state involving bridging of the Co and Zn centres by bromide and benzyl ligands. The most favourable pathway for this step involves initial substitution of a solvent molecule in BnZnBr(DMAc)_2_ with bromide coordinated to Co centre of **IX** leading to µ_2_ bromide bridging Zn and Co centres, (not depicted in Fig. 7) followed by transfer of benzyl *via* **TS4** (ΔG^‡^= 76.9 kJ/mol). The alternative manifolds involving pentavalent Zn-centre (**TS5**) or hexavalent Co-centre (**TS7**) are considerably higher in energy. Finally, intermediate **XII** undergoes relatively easy reductive elimination through **TS8** (ΔG^‡^= 54.9 kJ/mol). The resulting catalytic cycle is presented in Fig. 8.

## Conclusions

In conclusion, a straightforward method for performing the Negishi cross-coupling benzylation of aryl halides using the simplest cobalt precatalyst, cobalt bromide, in *N*,*N-*dimethylacetamide without addition of auxiliary ligands has been devised. With the use of the mild conditions described in the article, 26 products, including a variety of diarylmethanes, were synthesised, which was accomplished with yields comparable to those obtained with Pd catalysts. Additionally, the method has the potential to be extended to include alkyl alkenyl, and alkynyl iodides and bromides as coupling partners for arylmethylzinc bromides. At room temperature, the reaction is chemoselective toward aryl iodides, whereas carrying it out at elevated temperature allows for the transformation of aryl bromides, thus unlocking the possibility of sequential functionalisation. The role of solvent coordination in this transformation seems crucial and has been explained on the basis of DFT calculations.

## Methods

### General remarks

All reactions were carried out under argon atmosphere using standard Schlenk techniques and thoroughly dried glassware. Liquids were transferred using disposable syringes. *N*,*N*-dimethylacetamide (Merck/Sigma-Aldrich) was transferred to a Schlenk flask and degassed prior to use. Negishi reagents were prepared from the respective arylmethyl bromides in a direct reaction with zinc and their concentrations were determined by titration against iodine. Anhydrous cobalt(II) bromide and other reagents were purchased from Merck/Sigma-Aldrich and used as received.

### General procedure of Negishi cross coupling

A calculated volume of solution of arylmethylzinc bromide in THF containing 4 mmol (2 eq. respectively to aryl halide) of this reagent was placed in a carefully dried Schlenk bomb flask with a PTFE valve plug. The introduced THF was evaporated *in vacuo* through the Schlenk line. Then, dimethylacetamide (0.4 ml), the corresponding organic halide (1 eq., 2 mmol) and cobalt bromide (21.8 mg, 5 mol%, 0.1 mmol) were added. After closing, the reaction mixture was stirred for 20 h at room temperature (for ArI), or at 80 °C (for ArBr). The mixture was quenched with concentrated aqueous NH_4_Cl solution (10 ml) and ethyl acetate (10 ml) was added. The mixture was extracted with ethyl acetate (3 × 10 ml). The combined organic layers were washed with brine, dried over Na_2_SO_4,_ and concentrated *in vacuo*. Trace amounts of DMAc were removed by prolonged evacuation on the Schlenk line.

### Theoretical modelling

Calculations were conducted with Gaussian 16 package^43^. Structures of minima and transition states were optimized employing BP86 functional, def2-SVP basis set^44^ Frequency analysis was performed at the same level of theory to provide correction to thermodynamic functions and confirm the nature of optimized structures (minima and transition states featured zero and one imaginary frequency, respectively). Single point energies were calculated with BP86 functional employing def2-TZVPP^44^ basis set and solvation (*N*,*N*-dimethylacetamide) with the SMD model^45^. Molecular structures were visualized in CYLview^46^.

## Electronic supplementary material

Below is the link to the electronic supplementary material.

Supplementary Material 1

## Data Availability

All underlying data is available in the article itself and the accompanying Supplementary Material.
