# Supplementary material for: Synthesis of diarylmethanes by means of Negishi cross-coupling enabled by cobalt-solvent coordination
Source: Sci Rep. 2025 Jul 23;15:26809. doi: 10.1038/s41598-025-10180-1 (PMC12287326; doi:10.1038/s41598-025-10180-1)
Supplement: Supplementary file 1 — Supplementary Material 1 [file 41598_2025_10180_MOESM1_ESM.pdf]

Supporting Information

for

Synthesis of diarylmethanes by means of Negishi cross-coupling enabled by cobalt-solvent coordination

Jakub Robaszkiewicz[†‡], Wojciech Chaładaj[§], Piotr Pawluć[†‡], Maciej Zaranek[†*]

[†] Center for Advanced Technologies, Adam Mickiewicz University, Uniwersytetu Poznańskiego 10, 61-614 Poznań, Poland
[‡]Faculty of Chemistry, Adam Mickiewicz University, Uniwersytetu Poznańskiego 8, 61-614 Poznań, Poland
[§] Institute of Organic Chemistry, Polish Academy of Science, Kasprzaka 44/52, 01-224 Warszawa, Poland
*m.zaranek@amu.edu.pl

Contents

1. General remarks 2
2. General procedure of Negishi cross-coupling 2
3. Optimization of Negishi cross-coupling reaction – additional tables 3
4. SEM-EDS analysis 5
5. ICP-MS Analysis 7
6. Identification of Negishi cross-coupling products 8
7. Computational methods 48
8. References 85

1. General remarks

All reactions were carried out under argon atmosphere using standard Schlenk techniques and thoroughly dried glassware. Tetrahydrofuran was purified by distillation over sodium/benzophenone and stored under argon. *N,N*-dimethylacetamide (Merck/Sigma-Aldrich) was transferred to a Schlenk flask and degassed prior to use. Cobalt complexes were synthesized according to known literature procedures. Negishi reagents were prepared from the respective benzyl bromides in a direct reaction with zinc. Anhydrous cobalt(II) bromide and other reagents were purchased from Merck/Sigma-Aldrich and used as received. GC-MS analyses were performed using Agilent GC 8860 – MSD 5977B System. NMR spectra were collected using Bruker Avance Neo 300 MHz (^{1}H and ^{13}C) and Bruker Avance Neo 400 MHz (^{19}F) spectrometers with chemical shift scale referenced to residual solvent peaks assuming standardized values or, in case of ^{19}F, external reference and lock signal.

2. General procedure of Negishi cross-coupling

A calculated volume of solution of arylmethyl bromide in THF containing 4 mmol (2 eq. respectively to aryl halide) of this reagent was placed in a carefully dried Schlenk bomb flask with a PTFE valve plug. The introduced THF was evaporated *in vacuo* through the Schlenk line. Then, dimethylacetamide (0.4 ml), the corresponding organic halide (1 eq., 2 mmol) and cobalt bromide (21.8 mg, 5 mol%, 0.1 mmol) were added. After closing, the reaction mixture was stirred for 20 h at room temperature (for ArI), or at 80 °C (for ArBr). The mixture was quenched with concentrated aqueous NH_4Cl solution (10 ml) and ethyl acetate (10 ml) was added. The mixture was extracted with ethyl acetate (3 x 10 ml). The combined organic layers were washed with brine, dried over Na_2SO_4, and concentrated *in vacuo*. Trace amounts of DMAc were removed by prolonged evacuation on the Schlenk line.

3. Optimization of Negishi cross-coupling reaction – additional tables

Table S1. DMA as an additive to reactions carried out in THF.

Entry	DMA loading [mol %] (eqiv. rel. to Co)	Conversion of 4-iodotoluene [%]
1.	5 (1)	41
2.	10 (2)	47
3.	20 (4)	68
4.	30 (6)	85
5.	50 (19)	90
6.	100 (20)	93

Conditions: 4-iodotoluene 0.4 M in THF, [Negishi reagent]:[Ar-I]= 2:1, Cobalt bromide 5 mol %, temperature: 80 °C, inert gas.

Table S2. Using 3 mol% of cobalt bromide(II) as a precatalyst.

Table Entry	Time [hours]	Conversion of 4-iodotoluene [%]
1.	1	48
2.	2	57
3.	3	67
4.	6	80
5.	17	93

Conditions: 4-iodotoluene 5 M in DMA, [Negishi reagent]:[Ar-I]= 2:1, Cobalt bromide 3 mol %, room temperature, inert gas.

Table S3. Using 5 mol% of cobalt bromide(II) as a precatalyst.

Entry	Time [hours]	Conversion of 4-iodotoluene [%]
1.	1	77
2.	2	88
3.	3	92
4.	6	96
5.	17	99

Conditions: 4-iodotoluene 5 M in DMA, [Negishi reagent]:[Ar-I]= 2:1, Cobalt bromide 5 mol %, room temperature, inert gas.

4. SEM-EDS analysis

Chart S1a. SEM-EDS analysis of the solid residue after a completed reaction run.

kV: 30 Mag: 200 Takeoff: 34.8 Live Time(s): 50 Amp Time(μs): 1.92 Resolution:(eV) 123.8

10/4/2023 2:29:32 PM

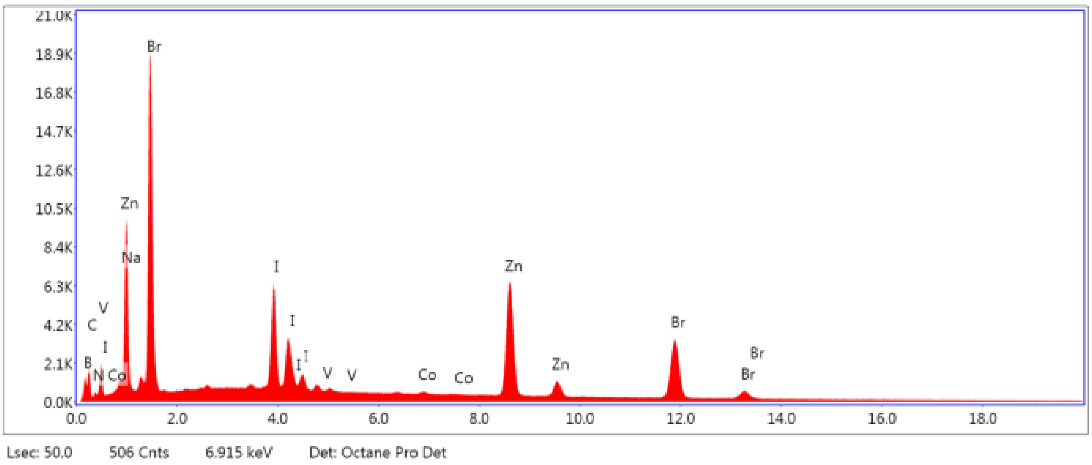

eZAF Smart Quant Results

Element	Weight %	Atomic %	Net Int.	Error %	Kratio	Z	R	A	F
B K	31.34	58.24	278.87	8.83	0.0935	1.0982	0.8896	0.2726	1.0000
C K	14.58	24.39	402.38	10.30	0.0189	1.1626	0.9031	0.1121	1.0000
V L	26.70	10.53	319.29	7.93	0.0872	0.8955	1.0568	0.3667	0.9979
I L	5.32	0.84	2590.15	3.67	0.0514	0.7766	1.2427	1.1777	1.0589
CoK	0.10	0.04	71.30	27.60	0.0010	0.8889	1.0608	0.9293	1.2131
ZnK	7.84	2.41	4030.48	2.64	0.0814	0.8786	1.0725	0.9722	1.2209
BrK	14.11	3.55	2487.20	2.78	0.1367	0.8174	1.0816	0.9914	1.2006

Chart S1b. SEM-EDS analysis of the solid residue after a completed reaction run (continuation).

kV: 30 Mag: 100 Takeoff: 35 Live Time(s): 163.8 Amp Time(µs): 1.92 Resolution:(eV) 123.8

map20231004143528146_0

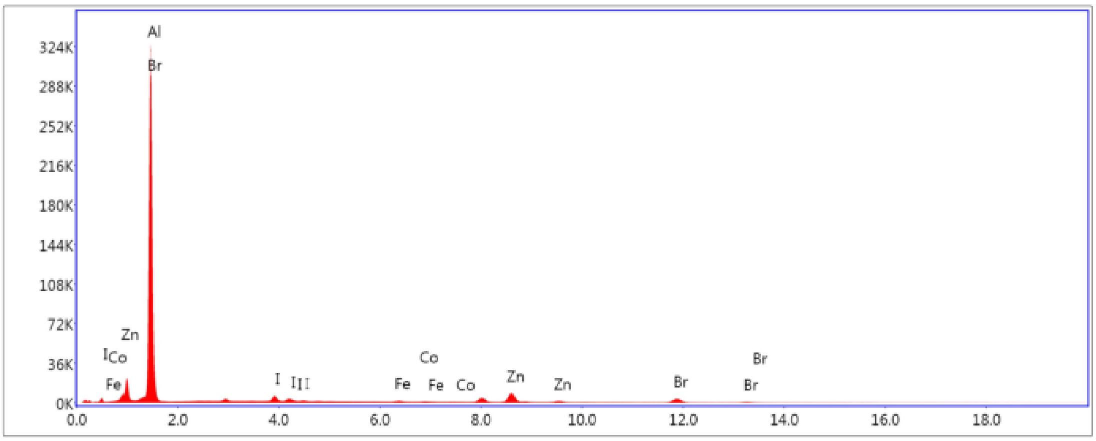

Lsec: 163.8 1.929K Cnts 6.410 keV Det: Octane Pro Det

eZAF Smart Quant Results

Element	Weight %	Atomic %	Net Int.	Error %	Kratio	Z	R	A	F
AlK	63.42	83.38	15371.31	5.89	0.3273	1.0650	0.9492	0.4836	1.0020
I L	6.26	1.75	697.10	2.52	0.0523	0.8106	1.2173	0.9858	1.0456
FeK	0.71	0.45	144.73	8.96	0.0072	0.9471	1.0369	0.9395	1.1318
CoK	0.30	0.18	56.28	13.75	0.0031	0.9279	1.0420	0.9572	1.1726
ZnK	12.39	6.72	1707.12	2.21	0.1306	0.9173	1.0552	0.9870	1.1646
BrK	16.92	7.51	805.13	2.77	0.1676	0.8541	1.0677	0.9925	1.1684

5. ICP-MS Analysis

Chart S2. SEM-EDS analysis of the solid residue after a completed reaction run.

Report Concentration Unit: ppb

Analyte Intensities

Analyte	Concentration	Intensity	Intensity Comment
H			Not Measured
He			Not Measured
Li	1.813	60008	
Be	0.007	71	
B	18.305	188546	
C	32856.074	49284111	
N	10332636.390	103326364	
O			Not Measured
F		0	
Ne		0	
Na	884.093	49067170	
Mg	166.052	7306301	
Al	137.531	7124104	
Si	3095.455	95959117	
P	11.524	59928	
S	0.000	0	
Cl	834167.134	333666854	
Ar	0.000	0	
K	497.744	9457139	
Ca	1620.215	33214412	
Sc	0.799	56179	
Ti	94.860	5293183	
V	315.598	17925942	
Cr	43.586	2780798	
Mn	0.506	34344	
Fe	0.000	0	
Co	25423.718	1372880799	
Ni	11.838	542197	
Cu	0.000	0	
Zn	173089.377	4656104250	
Ga	0.000	0	
Ge	0.000	0	
As	257.300	1458890	
Se	33278.430	133113721	
Br	S	S	
Kr	0.000	0	
Rb	0.000	0	
Sr	0.000	0	
Y	0.034	2553	

Analyte	Concentration	Intensity
Zr	0.000	0
Nb	0.137	6690
Mo	0.007	338
Ru	2.535	153361
Rh	0.824	46301
Pd	0.162	8851
Ag	0.012	648
Cd	0.046	2313
In	0.007	518
Sn	0.000	0
Sb	0.195	6229
Te	0.000	0
I	0.000	0
Xe	0.000	0
Cs	0.030	2102
Ba	0.000	0
La	0.002	162
Ce	1.323	98528
Pr	0.002	182
Nd	0.003	210
Sm	0.000	0
Eu	0.000	0
Gd	0.000	0
Tb	0.001	34
Dy	0.004	196
Ho	0.000	0
Er	0.000	0
Tm	0.000	21
Yb	0.000	0
Lu	0.000	0
Hf	0.006	246
Ta	0.000	17
W	0.018	556
Re	0.013	441
Os	0.000	0
Ir	0.000	0
Pt	0.125	2579
Au	0.000	0
Hg	0.000	0
Tl	0.003	159
Pb	0.000	0
Bi	0.055	2207
Th	0.000	9
U	0.019	996

TotalQuant Equations

Analyte	Equation
Ca	740.74*mass 43
Ti	13.44*mass 47
Se	11.455*mass 82
Fe	47.17*mass 57

Note: presented are the relative concentrations by weight of elements and calculated Pd concentration in this case is ≅6 × 10^{-7} mol per 1 mol of aryl halide, i.e., ≅0.6 mol ppb.

6. Identification of Negishi cross-coupling products

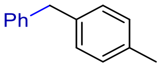

1-benzyl-4-methylbenzene (3a), Yield=96%, 348 mg

1H NMR (300 MHz, CDCl3) δ (ppm) 7.40 (m, 2H), 7.32 (m, 3H), 7.22 (s, 4H), 4.06 (s, 2H), 2.44 (s, 3H).

13C NMR (75 MHz, CDCl3) δ (ppm) 141.51, 138.17, 135.60, 129.24, 128.96, 128.91, 128.51, 126.06, 41.62, 21.09.

MS : m/z (%) 167 (100), 182 (80), 152 (30), 91 (20), 77 (17), 104 (16), 51(10), 128 (8).

Data conforms to the literature.[1]

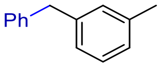

1-benzyl-3-methylbenzene (3b), Yield=84%, 306 mg

1H NMR (300 MHz, CDCl3) δ (ppm) 7.21 (m, 2H), 7.13 (m, 3H), 6.93 (m, 4H), 3.87 (s, 2H), 2.24 (s, 3H).

13C NMR (75 MHz, CDCl3) δ (ppm) 141.87, 141.51, 138.17, 135.60, 129.24, 128.96, 128.91, 128.54, 128.51, 128.42, 126.06, 126.01, 41.62, 21.09.

MS : m/z (%) 167 (100), 182 (60), 91 (25), 152 (25), 77 (22), 104 (21), 65 (20), 51 (15).

Data conforms to the literature.[1]

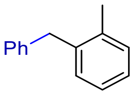

1-benzyl-2-methylbenzene (3c), Yield=74%, 270 mg

1H NMR (300 MHz, CDCl3) δ (ppm) 7.26 (m, 2H), 7.13 (m, 7H), 4.00 (s, 2H), 2.25 (s, 3H).

13C NMR (75 MHz, CDCl3) δ (ppm) 140.44, 138.97, 136.68, 130.33, 130.00, 128.79, 128.50, 128.43, 128.38, 126.50, 126.03, 125.96, 39.50, 19.72.

MS : m/z (%) 167(100), 182 (80), 165 (40), 152 (25), 104 (23), 91 (20), 65 (18), 128 (18), 51 (15).

Data conforms to the literature.[2]

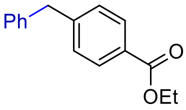

ethyl 4-benzylbenzoate (3d), Yield=87%, 417 mg

1H NMR (300 MHz, CDCl3) δ (ppm) 7.96 (d, 2H, J=8.3 Hz), 7.29 (m, 5H), 7.16 (d, J=8.3 Hz, 2H), 4.35 (q, J=7.12 Hz, 2H), 4.01 (s, 2H), 1.30 (t, J = 7.1 Hz, 3H).

13C NMR (75 MHz, CDCl3) δ (ppm) 166.5, 146.3, 140.1, 129.7, 128.9, 128.8, 128.5, 128.4, 126.3, 60.7, 41.8, 14.3.

MS : m/z (%) 167 (100), 195 (80), 240 (45), 152 (42), 91 (35), 212 (25), 115 (20), 65 (18).

Data conforms to the literature.[3]

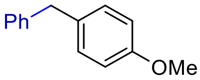

1-benzyl-4-methoxybenzene (3e), Yield=94%, 372 mg

1H NMR (300 MHz, CDCl3) δ (ppm) 7.16 (m, 2H), 7.07 (m, 3H), 6.98 (d, J=8.79 Hz, 2H), 6.71 (d, J=8.7 Hz, 2H), 3.80 (s, 2H), 3.63 (s, 3H).

13C NMR (75 MHz, CDCl3) δ (ppm) 158.10, 141.70, 133.37, 129.98, 128.93, 128.54, 126.09, 114.01, 55.31, 41.15.

MS : m/z (%) 198 (100), 167 (60), 121 (50), 153 (40), 91 (36), 77 (34), 183 (25), 128 (20), 51 (18).

Data conforms to the literature.[1]

<u>Gram-scale synthesis:</u>

1H NMR (300 MHz, CDCl3) δ (ppm) 7.17(m, 2H), 7.08(m, 3H), 7.00(d, J=8.82 Hz, 2H), 6.72(d, J=8.7 Hz, 2H), 3.82(s, 2H), 3.66(s, 3H).

13C NMR (75 MHz, CDCl3) δ (ppm) 158.06, 141.67, 133.33, 129.95, 128.90, 128.52, 126.06, 113.96, 55.30, 41.12.

MS : m/z (%) 198 (100), 167 (60), 121 (50), 153 (40), 91 (36), 77 (34), 183 (25), 128 (20), 51 (18).

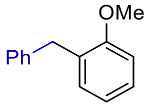

1-benzyl-2-methoxybenzene (3f), Yield=88%, 349 mg

1H NMR (300 MHz, CDCl3) δ (ppm) 7.12 (m, 6H), 6.96 (d, 1, J=7.3 Hz, 1H), 6.76 (t, J=9.0 Hz, 2H), 3.88 (s, 2H), 3.68 (s, 3H).

13C NMR (75 MHz, CDCl3) δ (8ppm) 157.3, 141.0, 130.3, 129.6, 128.9, 128.2, 127.4, 125.7, 120.4, 110.4, 55.3, 35.8.

MS : m/z (%) 198 (100), 165 (80), 91 (45), 183 (44), 152 (35), 77 (25), 115 (24), 128 (22), 139 (20).

Data conforms to the literature.[4]

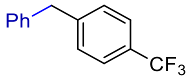

1-benzyl-4-(trifluoromethyl)benzene (3g), Yield=74%, 349 mg

1H NMR (300 MHz, CDCl3) δ (ppm) 7.45 (d, J = 8.0 Hz, 2H), 7.21 (m, 4H), 7.15 (m, 1H), 7.08 (m 2H), 3.94 (s, 2H).

13C NMR (75 MHz, CDCl3) δ (ppm) 145.25 (d, $^1J_{CF}$=5.13 Hz), 141.83, 140.01, 129.22, 128.97, 128.70, 128.49, 128.37, 128.30, 126.51, 126.15, 125.95, 125.45 (q, $^2J_{CF}$=15.06 Hz), 125.40, 122.55, 41.75.

19F NMR (376 MHz, CDCl3): δ (ppm) -63.00.

MS : m/z (%) 167 (100), 165 (49), 236 (43), 166 (20), 91 (19), 152 (16), 168 (15), 51 (11), 69 (10), 63 (9).

Data conforms to the literature.[1]

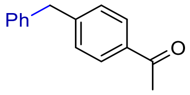

1-(4-benzylphenyl)ethan-1-one (3h), Yield=86%, 361 mg

1H NMR (300 MHz, CDCl3) δ (ppm) 7.80 (d, J = 8.4 Hz, 2H), 7.21 (m, 4H), 7.12 (m, 1H), 7.10 (m, 2H), 3.95 (s, 2H), 2.48 (s, 3H).

13C NMR (75 MHz, CDCl3) δ (ppm) 197.84, 146.87, 140.12, 135.34, 130.69, 129.14, 128.96, 128.67, 126.44, 41.93, 26.59.

MS : m/z (%) 195 (100), 165 (46), 210 (43), 91 (30), 65 (25), 115 (22), 139 (15).

Data conforms to the literature.[1]

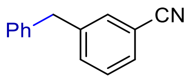

3-benzylbenzonitrile (3i), Yield=84%, 324 mg

1H NMR (300 MHz, CDCl3) δ (ppm) 7.50 (m, 2H), 7.45 (m, 1H), 7.42 (m, 1H), 7.34 (m, 2H), 7.28 (m, 1H), 7.19 (m, 2H), 4.03 (s, 2H).

13C NMR (75 MHz, CDCl3) δ (ppm) 142.6, 139.4, 133.4, 132.4, 129.9, 129.2, 128.9, 128.8, 126.7, 118.9, 112.5, 41.4.

MS : m/z (%) 193 (100), 165 (40), 91 (35), 191 (25), 63 (20), 178 (18), 51 (15).

Data conforms to the literature.[1]

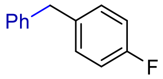

1-benzyl-4-fluorobenzene (3j), Yield=90%, 335 mg

1H NMR (300 MHz, CDCl3) δ (ppm) 7.32 (m, 2H), 7.24 (m, 1H), 7.17 (m, 4H), 6.99 (m, 2H), 3.97 (s, 2H).

13C NMR (75 MHz, CDCl3) δ (ppm) 161.44 (d, $^{1}J_{CF}$=243.8 Hz), 140.99, 136.79 (d, $^{4}J_{CF}$=3.4 Hz), 130.30 (d, $^{3}J_{CF}$=7.8 Hz), 128.85, 128.55, 126.22, 115.20 (d, $^{2}J_{CF}$=21.2 Hz), 41.10.

19F NMR (376 MHz, CDCl3): δ (ppm) -117.41.

MS : m/z (%) 186(100), 165 (60), 109 (30), 171 (24), 91 (22), 83 (20), 133 (15), 51 (15).

Data conforms to the literature.[1]

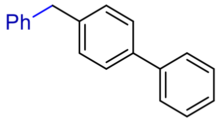

4-benzyl-1,1'-biphenyl (3k), Yield=75%, 366 mg

1H NMR (300 MHz, CDCl3) δ (ppm) 7.48 (m, 2H), 7.42 (m, 2H), 7.3 (t, J=7.68 Hz, 2H), 7.23 (m, 3H), 7.14 (m, 5H), 3.94 (s, 2H).

13C NMR (75 MHz, CDCl3) δ (ppm) 141.95, 141.05, 140.29, 139.09, 129.36, 129.01, 128.76, 128.56, 127.25, 127.11, 127.05, 126.19, 41.64.

MS : m/z (%) 244(100), 165 (60), 152 (35), 91 (34), 115 (28), 229 (25), 215 (20), 77 (18), 51 (15).

Data conforms to the literature.[5]

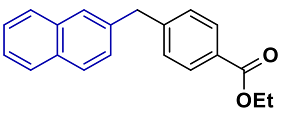 **ethyl 4-(naphthalen-2-ylmethyl)benzoate** (3l), Yield=64%, 333 mg

1H NMR (300 MHz, CDCl3) δ (ppm) 7.8 (m, 2H), 7.71(3H), 7.52 (s, 1H), 7.35 (m, 2H), 7.20 (m, 3H), 4.26 (q, J=7.11 Hz, 2H), 4.19 (s, 2H), 1.28 (t, J=7.11 Hz, 3H).

13C NMR (75 MHz, CDCl3) δ (ppm) 166.64, 137.68, 133.62, 132.20, 129.85, 129.05, 128.57, 128.30, 127.74, 127.68, 127.58, 127.46, 127.27, 126.16, 125.58, 60.88, 42.09, 14.37.

MS : m/z (%) 290 (100), 217 (82), 202 (45), 245 (42), 141 (22), 115 (16), 189 (14), 261 (12), 101 (8).

Data conforms to the literature.[6]

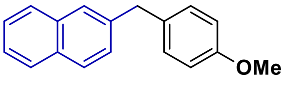 **2-(4-methoxybenzyl)naphthalene** (3m), Yield=75%, 372 mg

1H NMR (300 MHz, CDCl3) δ (ppm) 7.66 (m, 3H), 7.60 (s, 1H), 7.40 (m, 2H), 7.22 (d, J=7.6 Hz, 1H), 7.04 (d, J=8.73 Hz, 2H), 6.73 (d, J=8.7 Hz, 2H), 3.98 (s, 2H), 3.67 (s, 3H).

13C NMR (75 MHz, CDCl3) δ (ppm) 158.10, 139.14, 133.69, 133.16, 132.13, 130.03, 128.10, 127.65, 127.61, 126.97, 126.01, 125.36, 125.22, 113.99, 55.30, 41.27.

MS : m/z (%) 248 (100), 217 (55), 202 (49), 121 (24), 141 (22), 233 (18), 101 (16), 77 (14), 189 (12), 178 (11).

Data conforms to the literature.[7]

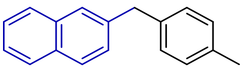 **2-(4-methylbenzyl)naphthalene** (3n), Yield=86%, 399mg

1H NMR (300 MHz, CDCl3) δ (ppm) 7.66 (m, 3H), 7.52 (s, 1H), 7.31 (m, 2H), 7.20 (dd, J = 8.46, 1.68 Hz, 1H), 7.01 (m, 4H), 3.99 (s, 2H), 2.22 (s, 3H).

13C NMR (75 MHz, CDCl3) δ (ppm) 138.98, 138.01, 135.71, 133.70, 132.15, 129.27, 128.99, 128.12, 127.71, 127.70, 127.63, 127.08, 126.01, 125.37, 41.76, 21.11.

MS : m/z (%) 232 (100), 217 (86), 115 (60), 202 (36), 141 (33), 77 (28), 91(27), 63 (22), 51 (20), 127 (20).

Data conforms to the literature.[8]

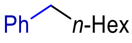 **heptylbenzene** (3o), Yield=58%, 204 mg

1H NMR (300 MHz, CDCl3) δ (ppm) 7.18 (t, J=8.2 Hz, 2H), 7.10 (d, J=7.2 Hz, 3H), 2.51 (t, J = 7.53 Hz, 2H), 1.54 (t, J=7.5 Hz, 2H), 1.21 (m, 8H), 0.81 (t, J=7.1 Hz, 3H).

13C NMR (75 MHz, CDCl3) δ (ppm) 143.00, 128.45, 128.26, 125.59, 36.07, 31.90, 31.60, 29.38, 29.26, 22.74, 14.15.

MS : m/z (%) 91 (100), 65(27), 182 (25), 51 (10), 104 (8), 77 (6), 165 (4).

Data conforms to the literature.[9]

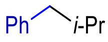 **isobutylbenzene** (3p), not isolated

MS : m/z (%) 91 (100), 134 (40), 65 (20), 115 (10), 51 (6), 89 (6), 103 (4).

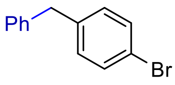 **1-benzyl-4-bromobenzene**, (3q), Yield=81%, 403 mg

1H NMR (300 MHz, CDCl3) δ (ppm) 7.32 (m, 2H),7.21 (m, 2H), 7.14 (m, 1H), 7.08 (m, 2H), 6.97 (m, 2H), 3.85 (s, 2H).

13C NMR (75 MHz, CDCl3) δ (ppm) 140.47, 140.13, 131.54, 130.69, 128.89, 128.60, 126.33, 119.97, 41.33.

MS : m/z (%) 91 (100), 165 (91), 167 (67), 195 (51), 104 (44), 152 (36), 89 (34), 51 (31), 240 (30).

Data conforms to the literature.[10]

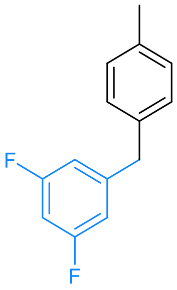 **1,3-difluoro-5-(4-methylbenzyl)benzene**, (3r), X=I: Yield=87%, 189 mg; X=Br: Yield=64%, 140 mg

1H NMR (300 MHz, CDCl3) δ (ppm) 7.10(m, 4H), 6.65(m, 3H), 3.91(s, 2H), 2.34(s, 3H).

13C NMR (75 MHz, CDCl3) δ (ppm) 163.06 (dd, $^1J_{CF}$=248.0 Hz, $^3J_{CF}$=12.9 Hz), 145.42 (t, $^3J_{CF}$=8.8 Hz), 136.40, 136.23, 129.41, 128.83, 111.59 (dd, $^2J_{CF}$=16.8 Hz, $^4J_{CF}$=7.6 Hz), 101.50 (t, $^2J_{CF}$=25.4 Hz), 41.21, 21.04.

19F NMR (376 MHz, CDCl3): δ (ppm) -109,03, -109,88.

MS : m/z (%) 203 (100), 218 (86), 183 (43), 201 (36), 202 (21), 91 (19), 105 (16).

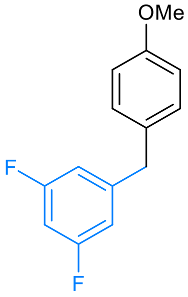 **1,3-difluoro-5-(4-methoxybenzyl)benzene**, (3s), Yield=90%, 210 mg

1H NMR (300 MHz, CDCl3) δ (ppm) 7.09(m, 2H), 6.86(m, 2H), 6.66(m, 3H), 3.89(s, 2H), 3.80(s, 3H).

13C NMR (75 MHz, CDCl3) δ (ppm) 163.06 (dd, $^1J_{CF}$=248.0 Hz, $^3J_{CF}$=12.9 Hz), 158.37, 145.63 (t, $^3J_{CF}$=8.8 Hz), 131.51, 129.95, 114.12, 111.52 (dd, $^2J_{CF}$=16.8 Hz, $^4J_{CF}$=7.6 Hz), 101.48 (t, $^2J_{CF}$=25.4 Hz), 55.27, 40.52.

19F NMR (376 MHz, CDCl3): δ (ppm) -110.45.

MS : m/z (%) 234 (100), 121 (64), 233 (31), 170 (24), 77 (24), 201 (23), 63 (23), 203 (22).

HRMS (APCI): found: 247.0941, calc. for $C_{15}H_{13}OF_2$: 247.0934, diff. 2.8 ppm.

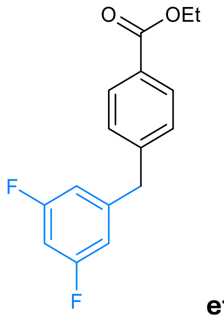

ethyl 4-(3,5-difluorobenzyl)benzoate, (3t), X=I: Yield=73%, 202 mg; X=Br: Yield=71%, 195 mg

1H NMR (300 MHz, CDCl3) δ (ppm) 7.92(m, 2H), 7.16(m, 2H), 6.60(m, 3H), 4.30(q, J=7.11 Hz, 2H), 3.92(s, 2H), 1.31(t, J=7.14 Hz, 3H).

13C NMR (75 MHz, CDCl3) δ (ppm) 166.40, 163.12 (dd, $^{1}J_{CF}$=248.6 Hz, $^{3}J_{CF}$=12.8 Hz), 144.53, 130.02, 128.93, 111.71 (dd, $^{2}J_{CF}$=17.7 Hz, $^{4}J_{CF}$=7.6 Hz), 101.94(t, $^{2}J_{CF}$=25.3 Hz), 60.96, 41.50, 14.35.

19F NMR (376 MHz, CDCl3): δ (ppm) -109,94.

MS : m/z (%) 231 (100), 183 (53), 201 (39), 203 (37), 276 (31), 248 (22), 232 (19), 127 (12).

HRMS (APCI): found: 277.1045, calc. for $C_{15}H_{13}O_2F_2$: 277.1040, diff. 1.8 ppm.

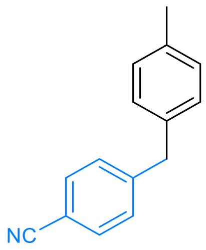

4-(4-methylbenzyl)benzonitrile, (3u), Yield=78%, 162 mg

1H NMR (300 MHz, CDCl3) δ (ppm) 7.48(d, J=8.52 Hz, 2H), 7.20(d, J=8.67 Hz, 2H), 7.04(d, J=8.22, 2H), 6.96(d, J=7.92 Hz, 2H), 3.92(s, 2H), 2.25(s, 3H).

13C NMR (75 MHz, CDCl3) δ (ppm) 147.08, 136.29, 132.29, 129.59, 129.46, 128.85, 126.82, 119.05, 109.95, 41.58, 21.03.

MS : m/z (%) 192 (100), 91 (63), 63 (50), 89 (47), 190 (43), 65 (43), 165 (31), 191 (29).

Data conforms to the literature.[11]

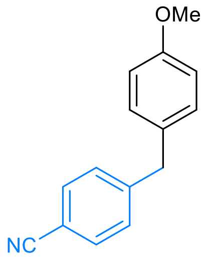

4-(4-methoxybenzyl)benzonitrile, (3v), Yield=77%, 171 mg

1H NMR (300 MHz, CDCl3) δ (ppm) 7.54(m, 2H), 7.24(m, 2H), 7.06(m, 2H), 6.83(m, 2H), 3.95(s, 2H), 3.77(s, 3H).

13C NMR (75 MHz, CDCl3) δ (ppm) 158.38, 147.27, 132.29, 131.41, 129.97, 129.53, 127.74, 114.18, 109.93, 55.30, 41.11.

MS : m/z (%) 223 (100), 121 (65), 77 (35), 222 (32), 208 (29), 180 (28), 51 (25), 190 (23).

Data conforms to the literature.[12]

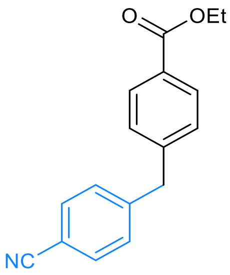

ethyl 4-(4-cyanobenzyl)benzoate, (3w), X=I: Yield=68%, 180 mg ; X=Br: Yield=56%, 148 mg

1H NMR (300 MHz, CDCl3) δ (ppm) 7.92(m, 2H), 7.51(m, 2H), 7.17(m, 4H), 4.30(q, J=7.1 Hz, 2H), 4.01(s, 2H), 1.31(t, J=7.1 Hz, 3H).

13C NMR (75 MHz, CDCl3) δ (ppm) 166.15, 145.90, 144.39, 132.45, 132.30, 130.07, 129.67, 129.04, 128.96, 110.21, 60.79, 41.76, 14.26.

MS : m/z (%) 220 (100), 190 (43), 165 (39), 191 (29), 220 (23), 264 (22), 237 (22).

Data conforms to the literature.[13]

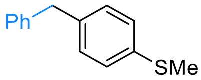

(4-benzylphenyl)(methyl)sulfane, (3y), Yield=79%, 169 mg

1H NMR (300 MHz, CDCl3) δ (ppm) 7.18 (m, 2H), 7.09 (m, 5H), 7.01 (m, 2H), 3.84 (s, 2H), 2.35 (s, 3H).

13C NMR (75 MHz, CDCl3) δ (ppm) 141.04, 138.26, 135.81, 129.52, 128.93, 128.55, 127.18, 126.19, 41.43, 16.26.

MS : m/z (%) 167 (100), 214 (83), 165 (79), 166 (35), 152 (30), 91 (24), 137 (18).

Data conforms to the literature.[14]

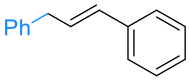

(*E*)-prop-1-ene-1,3-diyldibenzene, (3x), Yield=87%, 169 mg

1H NMR (300 MHz, CDCl3) δ (ppm) 7.16(m, 5H), 7.06(m, 5H), 6.23(m, 2H), 3.42(d, J=6.27 Hz, 2H).

13C NMR (75 MHz, CDCl3) δ (ppm) 139.53, 136.83, 130.43, 128.59, 128.04, 127.86, 126.47, 125.55, 125.49, 38.72.

MS : m/z (%) 115 (100), 91 (60), 194 (46), 77(42), 51 (40), 65 (38), 179 (36), 103 (34).

Data conforms to the literature.[15]

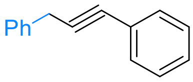

prop-1-yne-1,3-diyldibenzene, (3z), Yield=67%, 128 mg

1H NMR (300 MHz, CDCl3) δ (ppm) 7.44(m, 4H), 7.30(m, 6H), 3.84(s, 2H).

13C NMR (75 MHz, CDCl3) δ (ppm) 136.78, 131.66, 128.57, 128.25, 127.98, 127.84, 126.66, 123.70, 87.53, 82.68, 25.77.

MS: m/z (%) 192 (100), 91 (67), 115 (52), 77 (45), 89 (41), 63 (28), 103 (26), 65 (23).

Data conforms to the literature.[16]

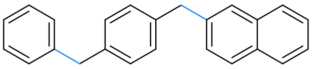 **2-(4-benzylbenzyl)naphthalene**, (3aa), Yield=78%, 240 mg

1H NMR (300 MHz, CDCl3) δ (ppm) 7.67(m, 4H), 7.35(m, 5H), 7.10(m, 12H), 4.00(s, 2H), 3.85(s, 2H).

13C NMR (75 MHz, CDCl3) 138.77, 138.75, 132.12, 129.34, 129.13, 129.09, 129.02, 128.97, 128.57, 128.50, 128.11, 127.70, 127.67, 127.60, 127.11, 126.09, 126.01, 125.37, 41.77, 41.60.

MS : m/z (%) 90 (100), 139 (78), 162 (74), 139 (70), 113 (68), 126 (56), 76 (42), 212 (34).

HRMS (APCI): found: 308.1564 , calc. for $C_{24}H_{20}$: 308.1565 , diff. 0.3 ppm.

3a

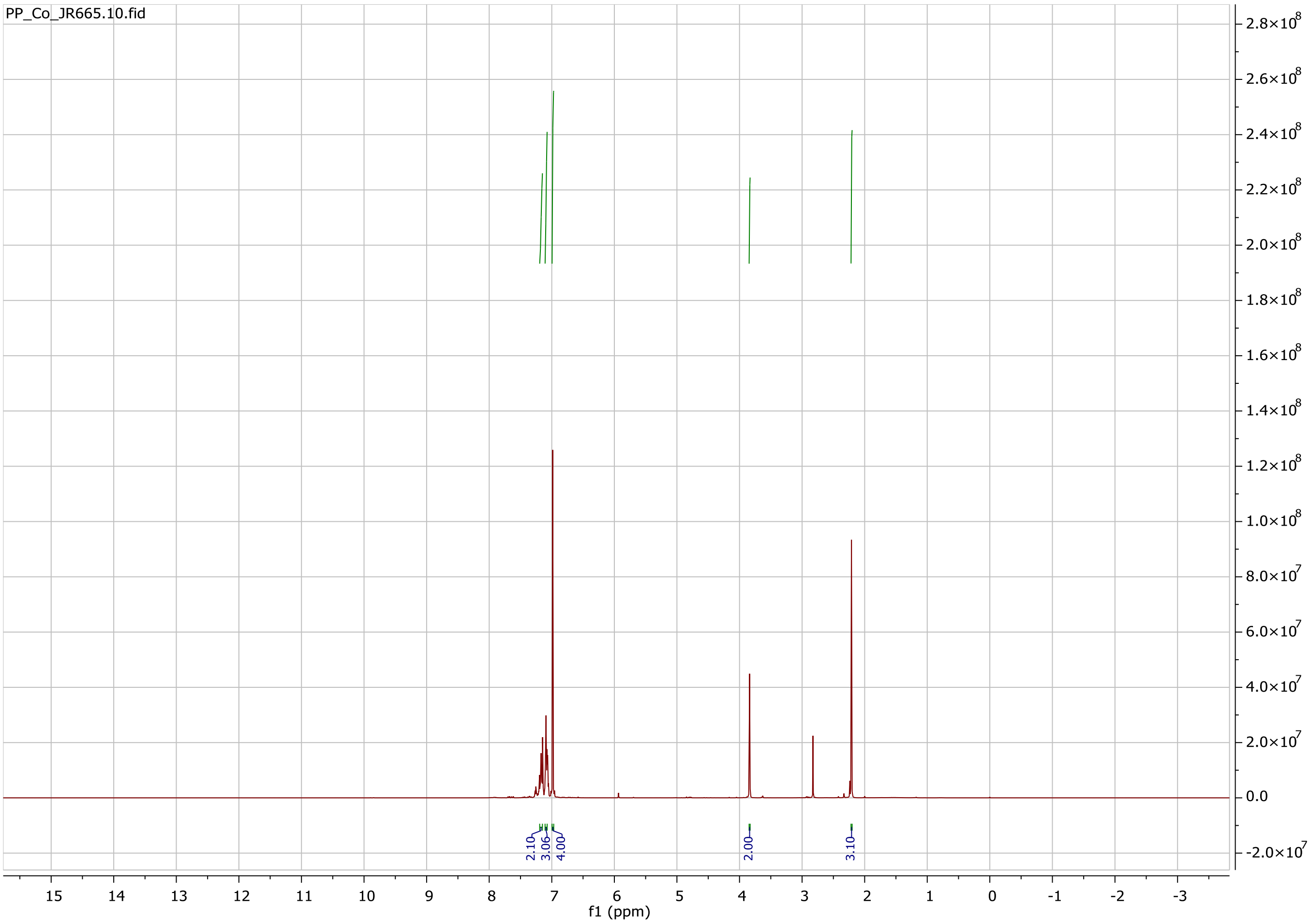

PP_Co_JR665.10.fid
2.10
3.06
4.00
2.00
3.10
f1 (ppm)

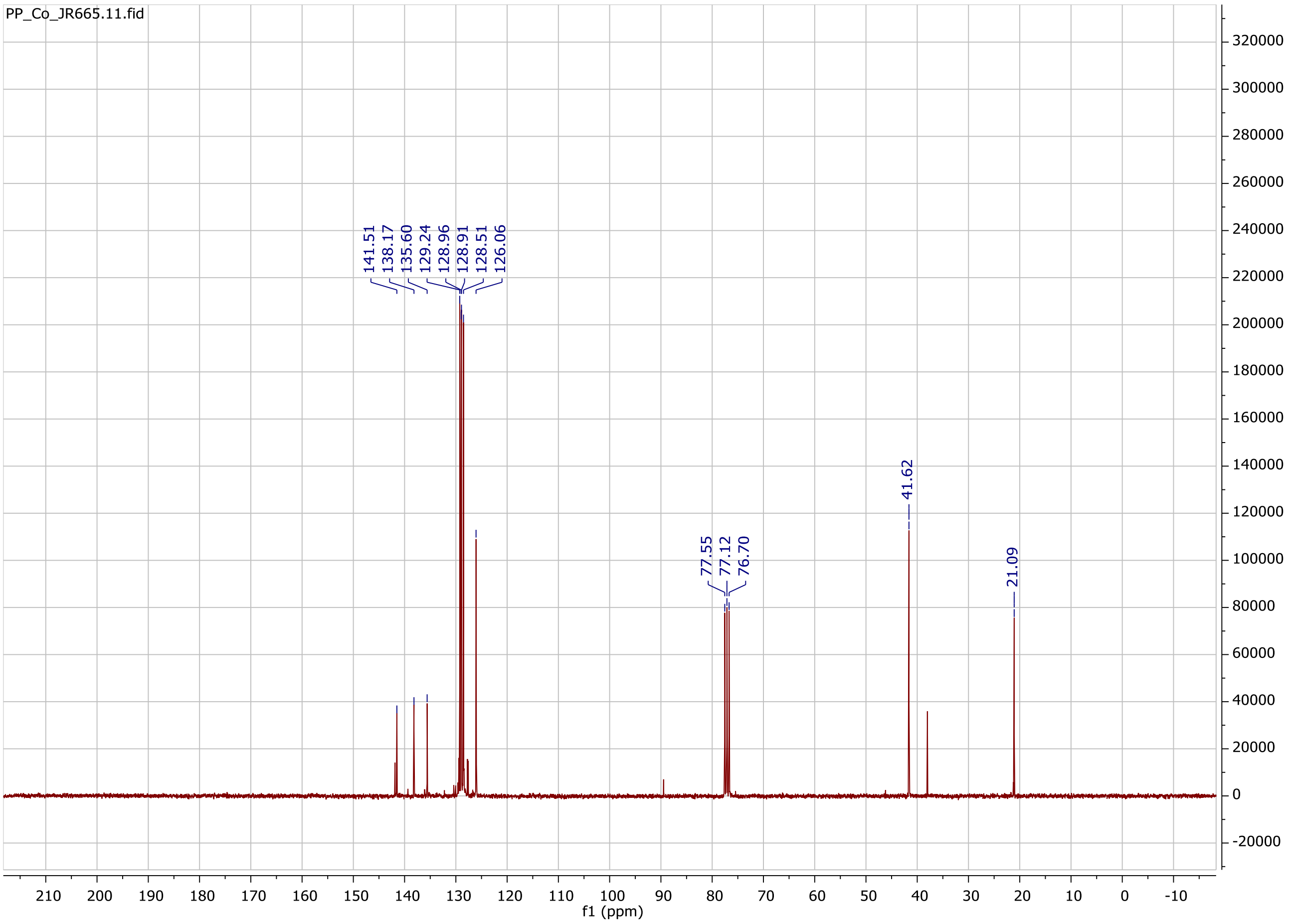

PP_Co_JR665.11.fid
141.51
138.17
135.60
129.24
128.96
128.91
128.51
126.06
77.55
77.12
76.70
41.62
21.09
f1 (ppm)

3b

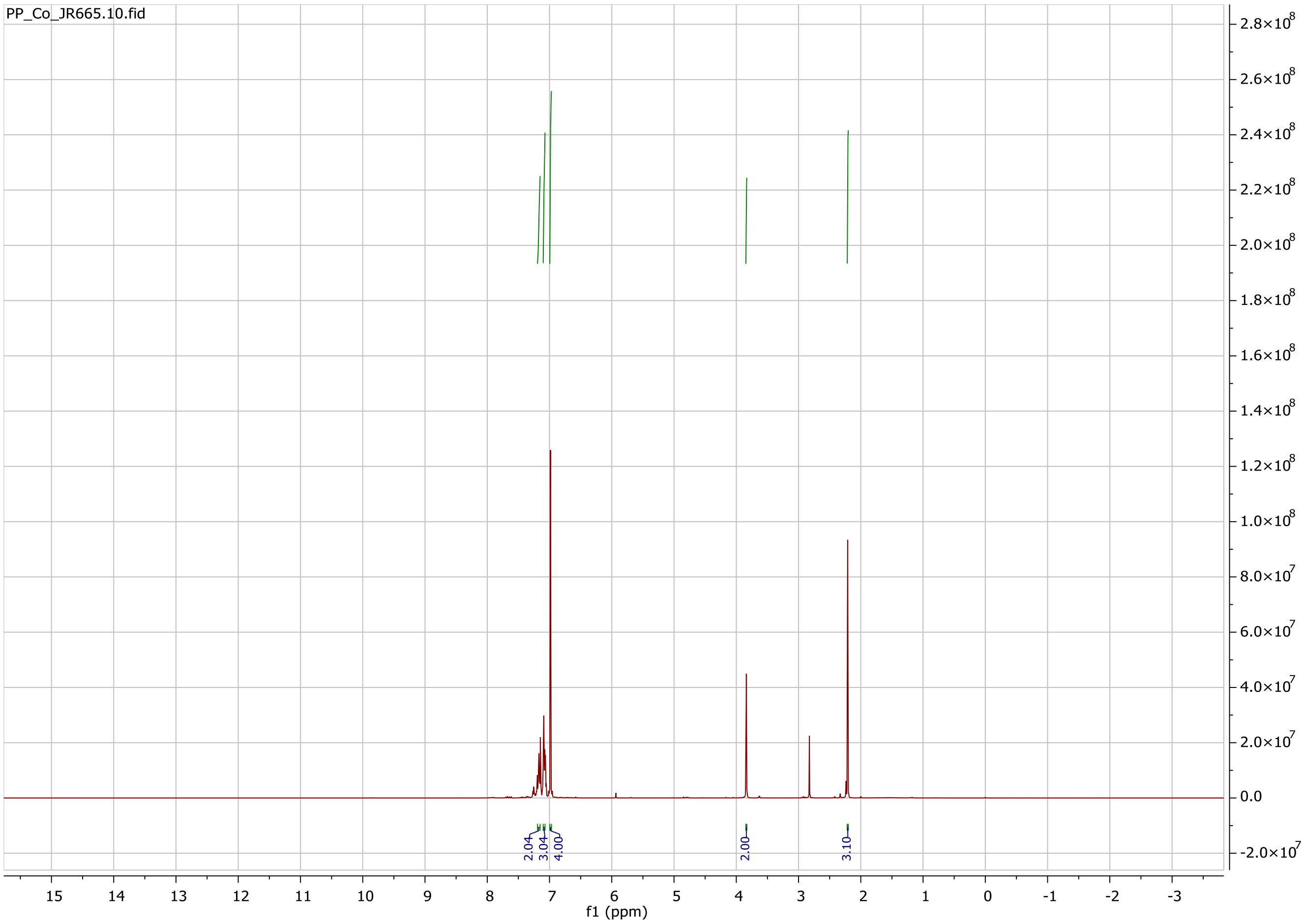

PP_Co_JR665.10.fid
2.04
3.04
4.00
2.00
3.10
f1 (ppm)

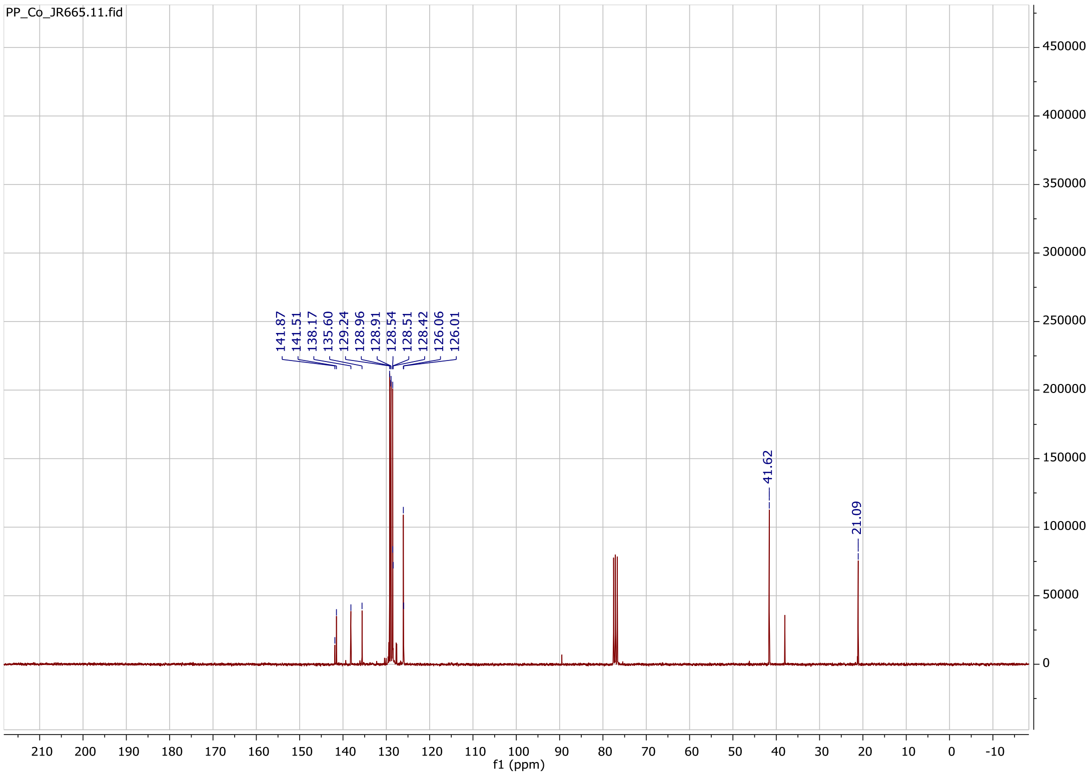

PP_Co_JR665.11.fid
141.87
141.51
138.17
135.60
129.24
128.96
128.91
128.54
128.51
128.42
126.06
126.01
41.62
21.09
f1 (ppm)

3c

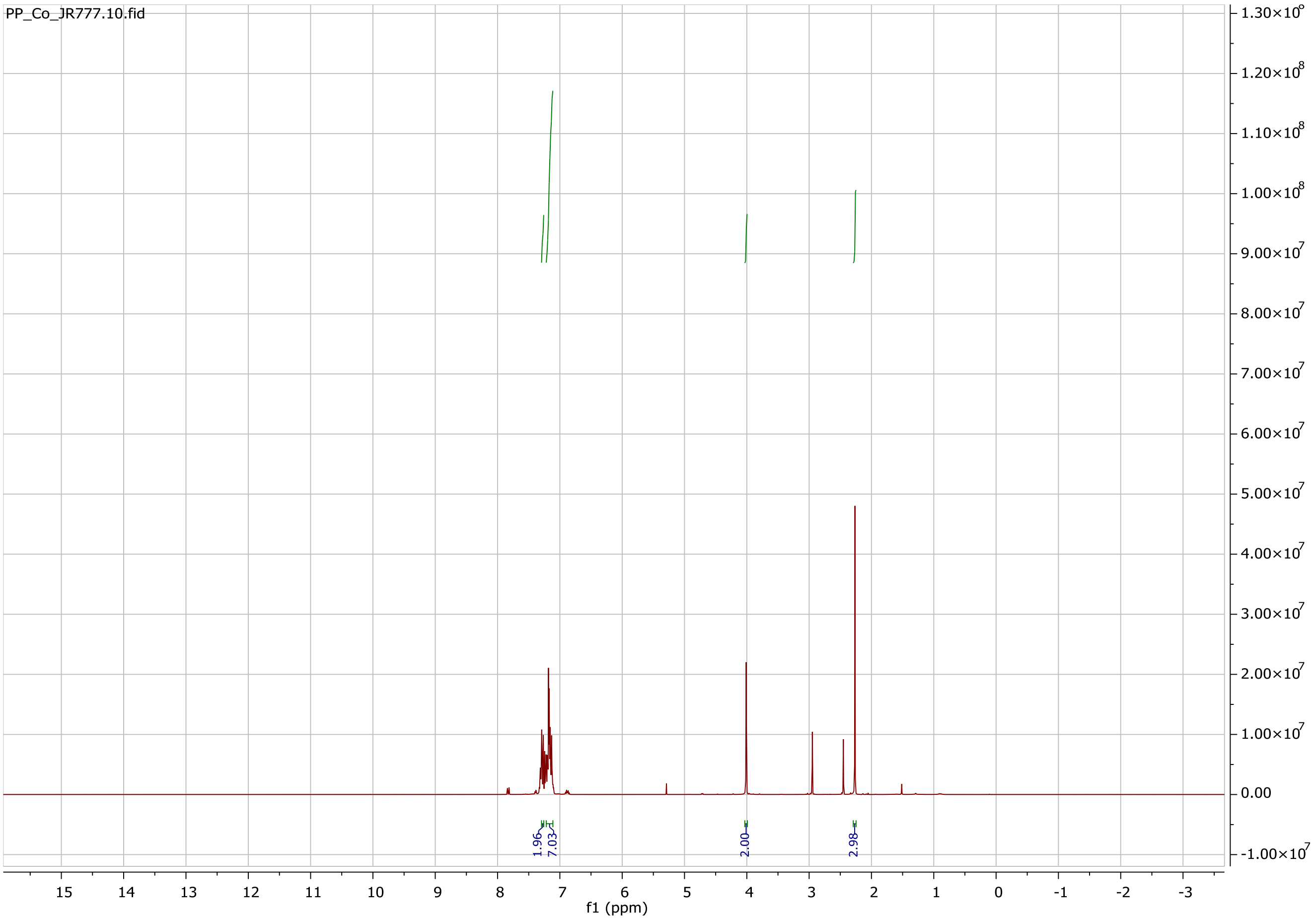
PP_Co_JR777.10.fid
1.96
7.03
2.00
2.98
f1 (ppm)

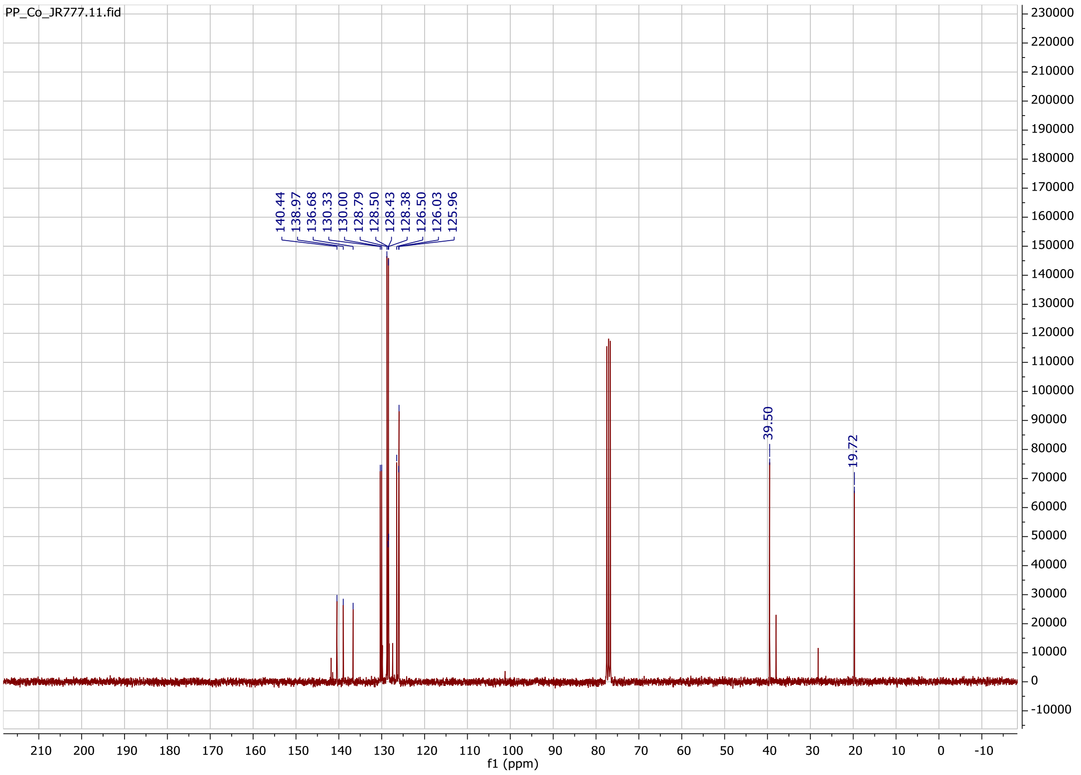
PP_Co_JR777.11.fid
140.44
138.97
136.68
130.33
130.00
128.79
128.50
128.43
128.38
126.50
126.03
125.96
39.50
19.72
f1 (ppm)

3d

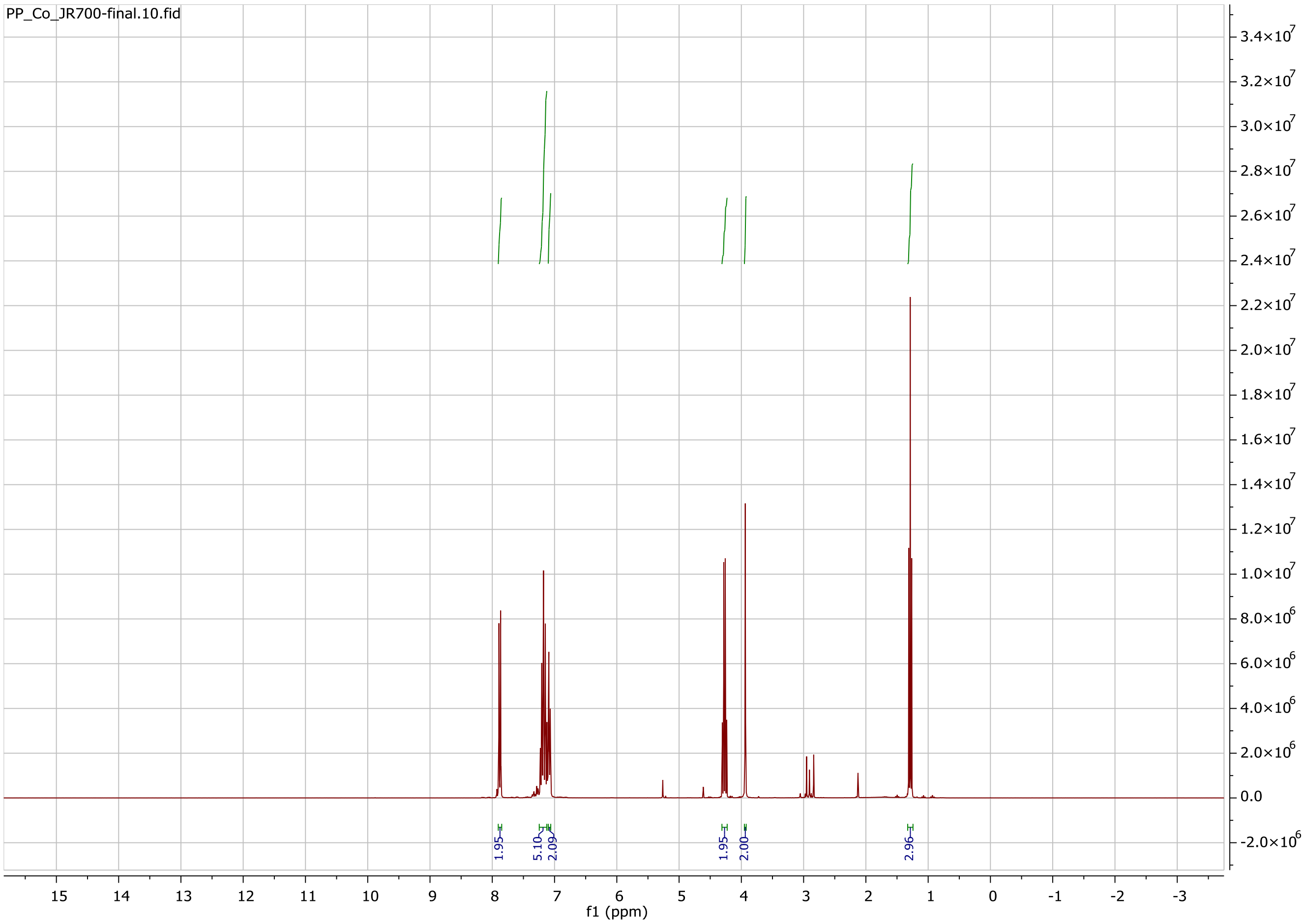
PP_Co_JR700-final.10.fid
1.95
5.10
2.09
1.95
2.00
2.96
f1 (ppm)

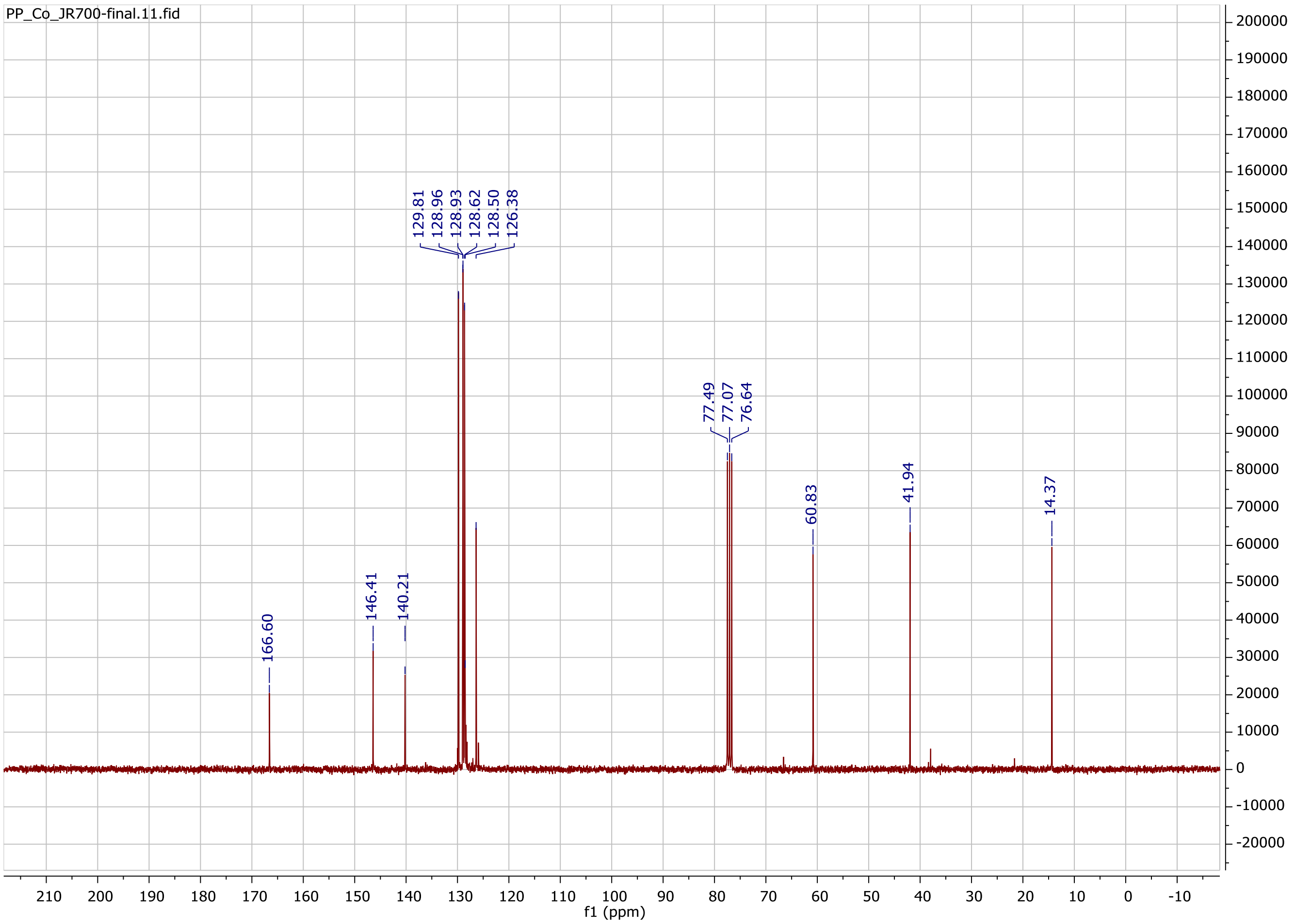
PP_Co_JR700-final.11.fid
129.81
128.96
128.93
128.62
128.50
126.38
77.49
77.07
76.64
166.60
146.41
140.21
60.83
41.94
14.37
f1 (ppm)

3e

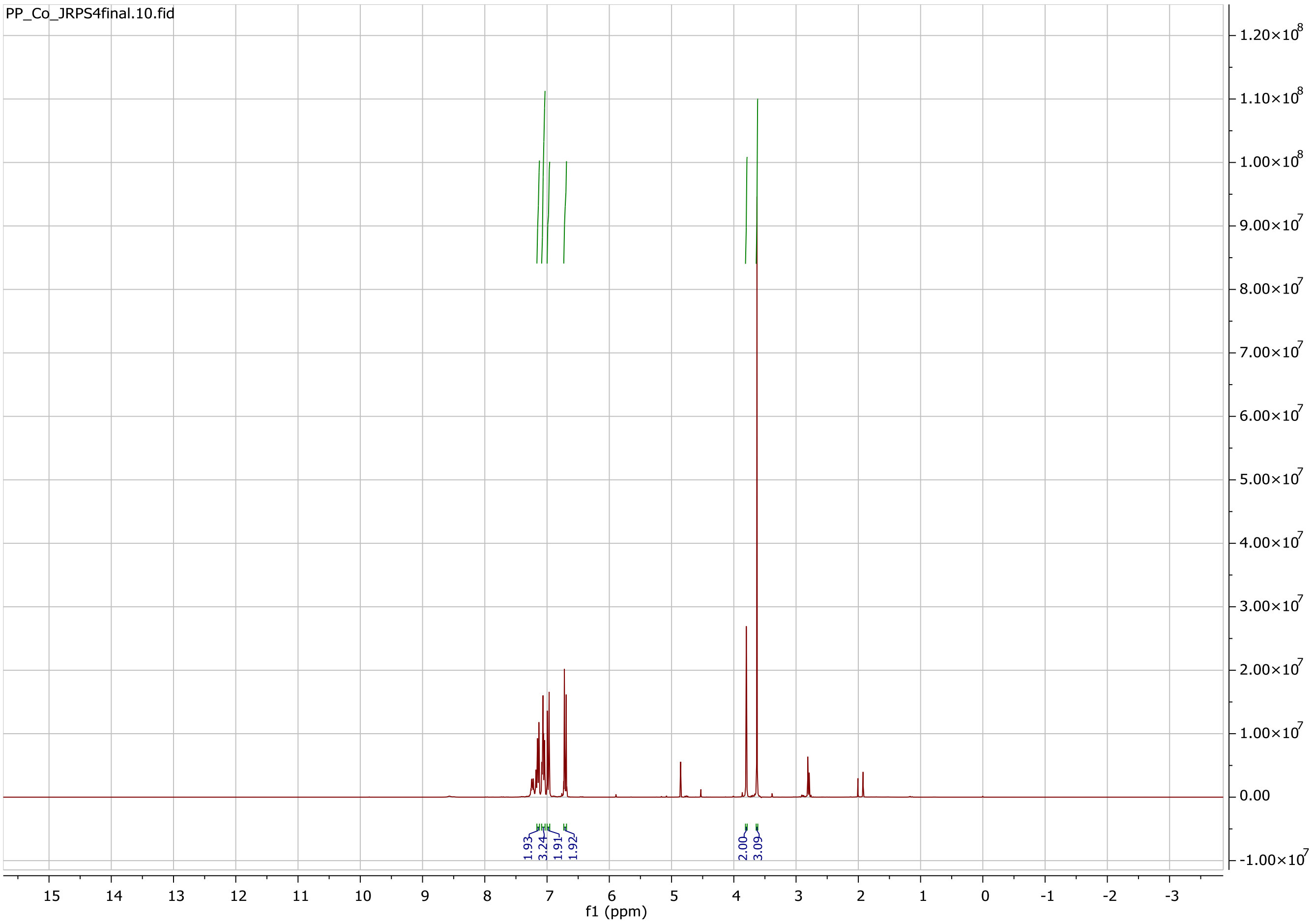
PP_Co_JRPS4final.10.fid
1.93
3.24
1.91
1.92
2.00
3.09
f1 (ppm)

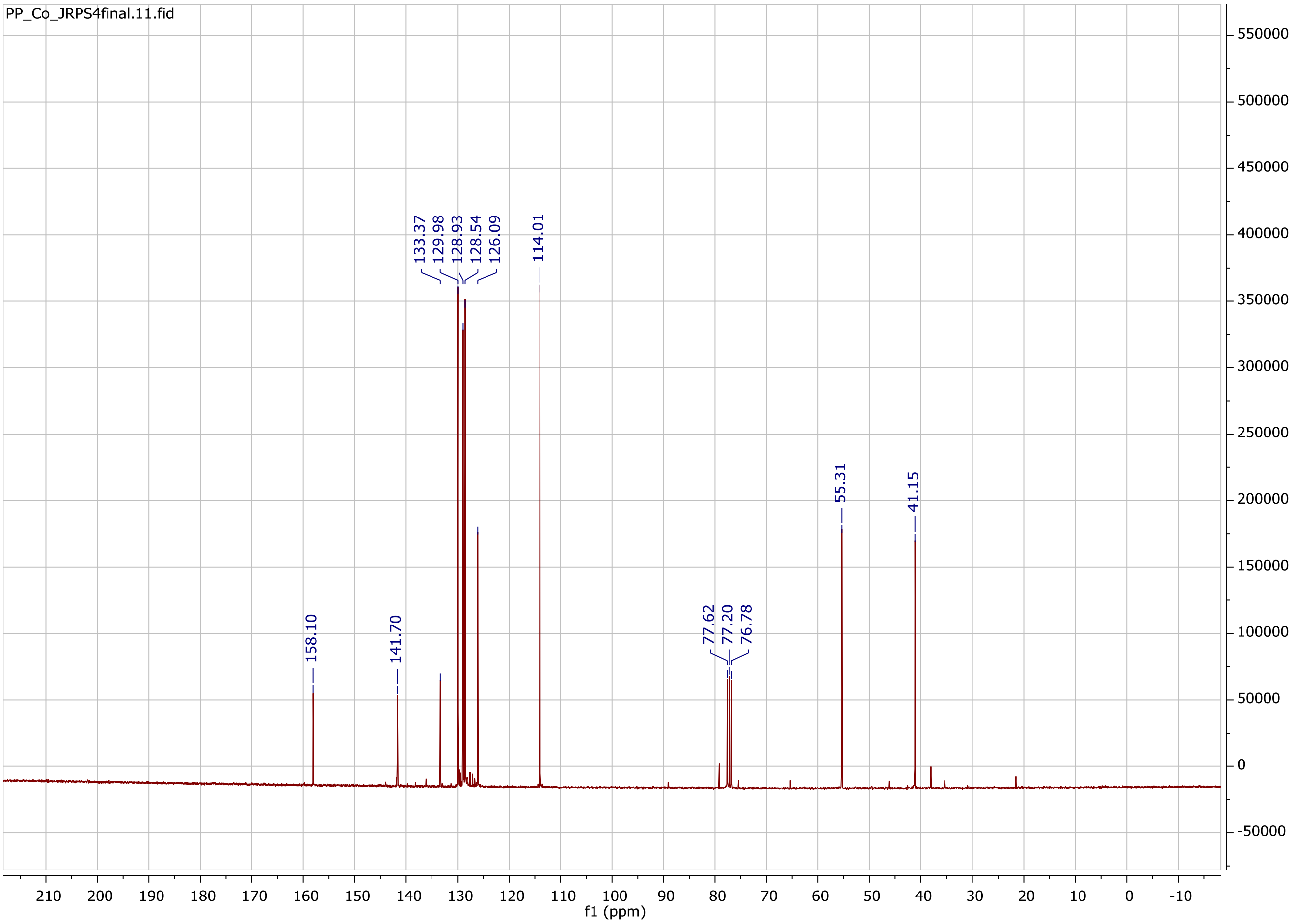
PP_Co_JRPS4final.11.fid
133.37
129.98
128.93
128.54
126.09
114.01
158.10
141.70
77.62
77.20
76.78
55.31
41.15
f1 (ppm)

3e GRAM-SCALE

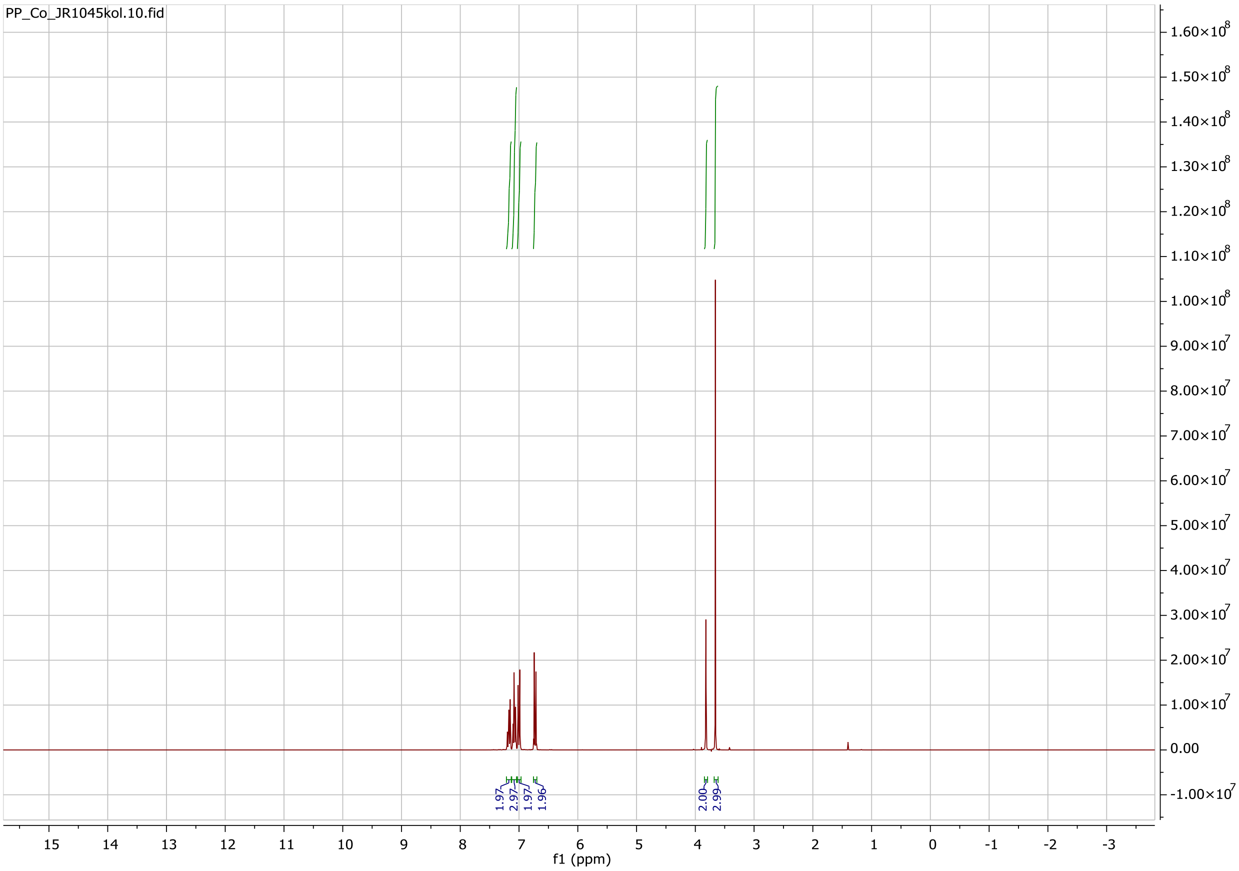

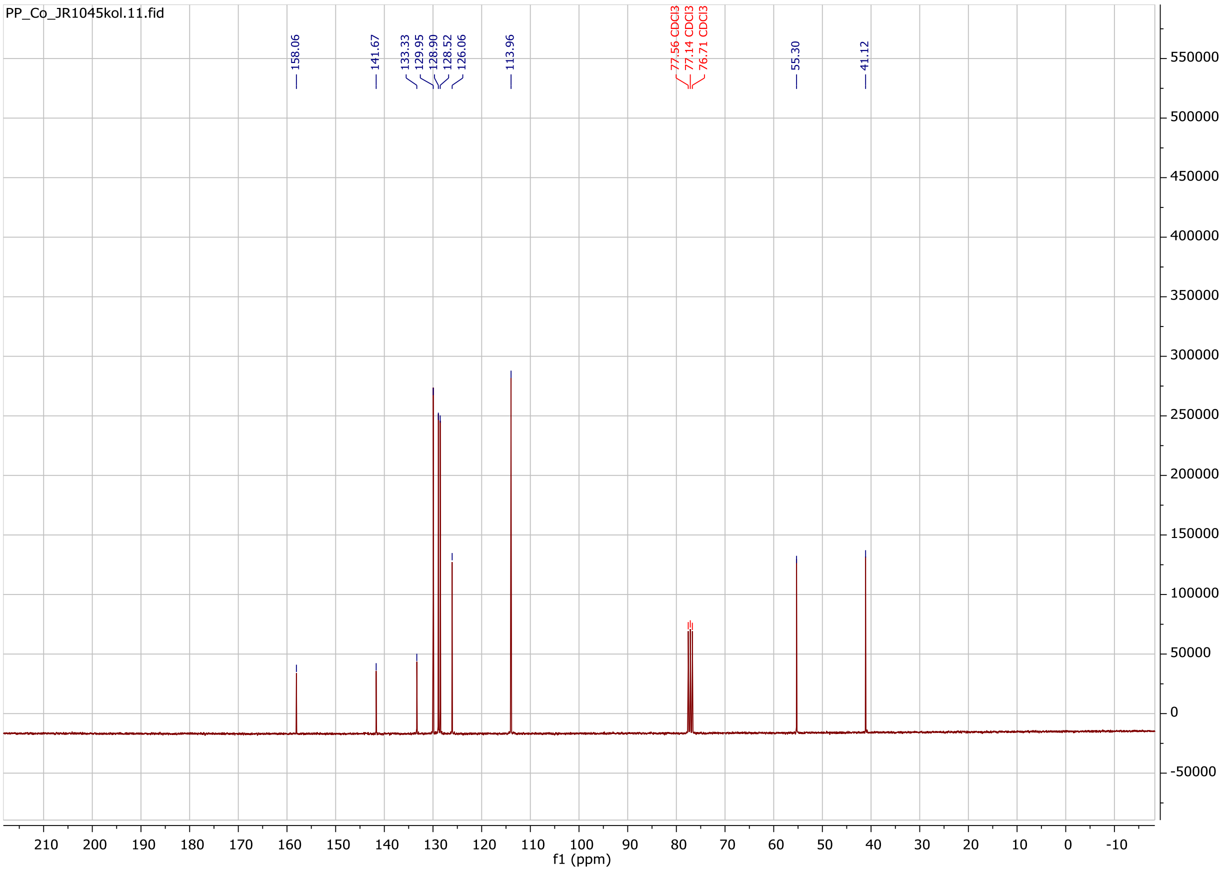

3f

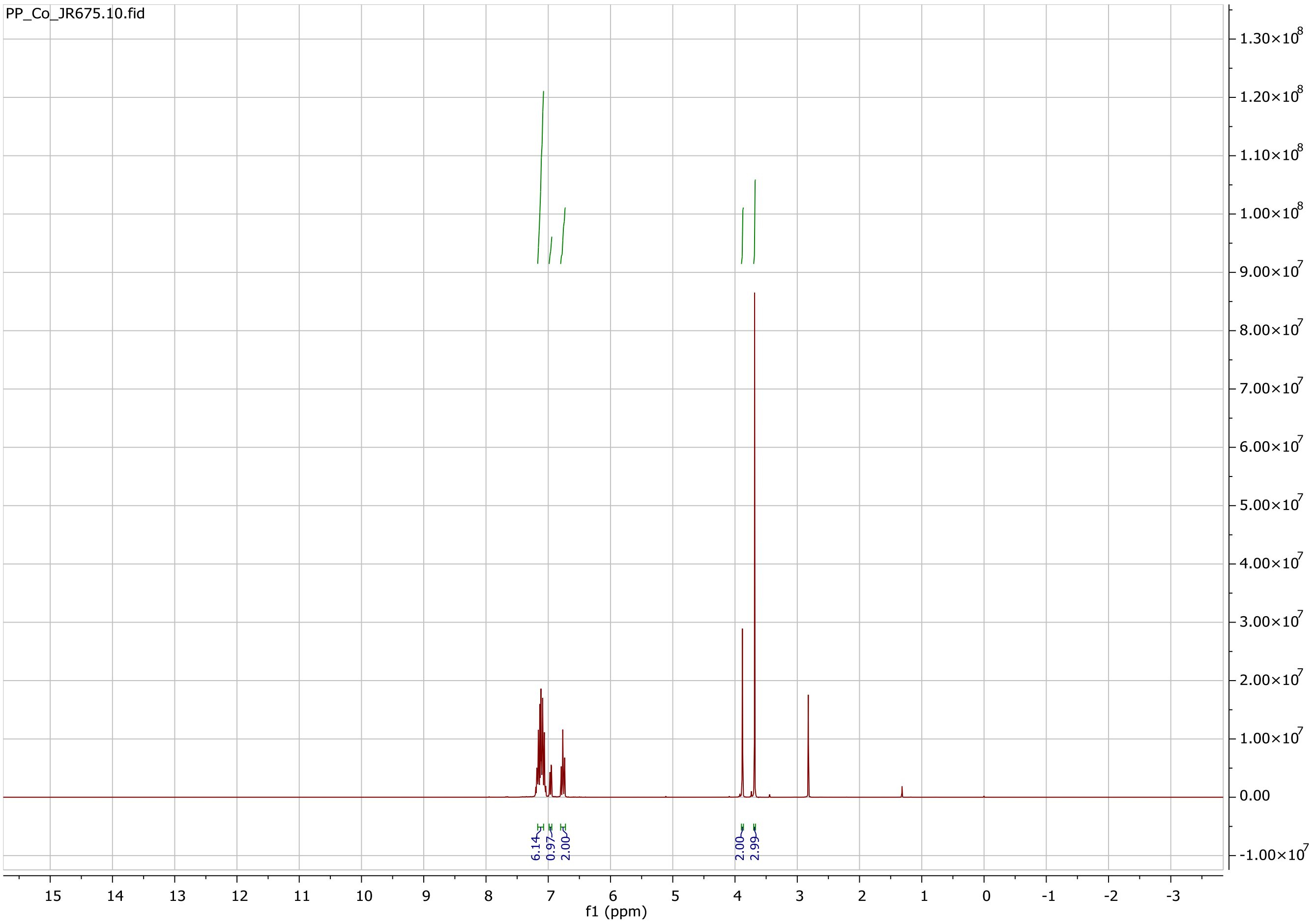
PP_Co_JR675.10.fid
6.14
0.97
2.00
2.00
2.99
f1 (ppm)

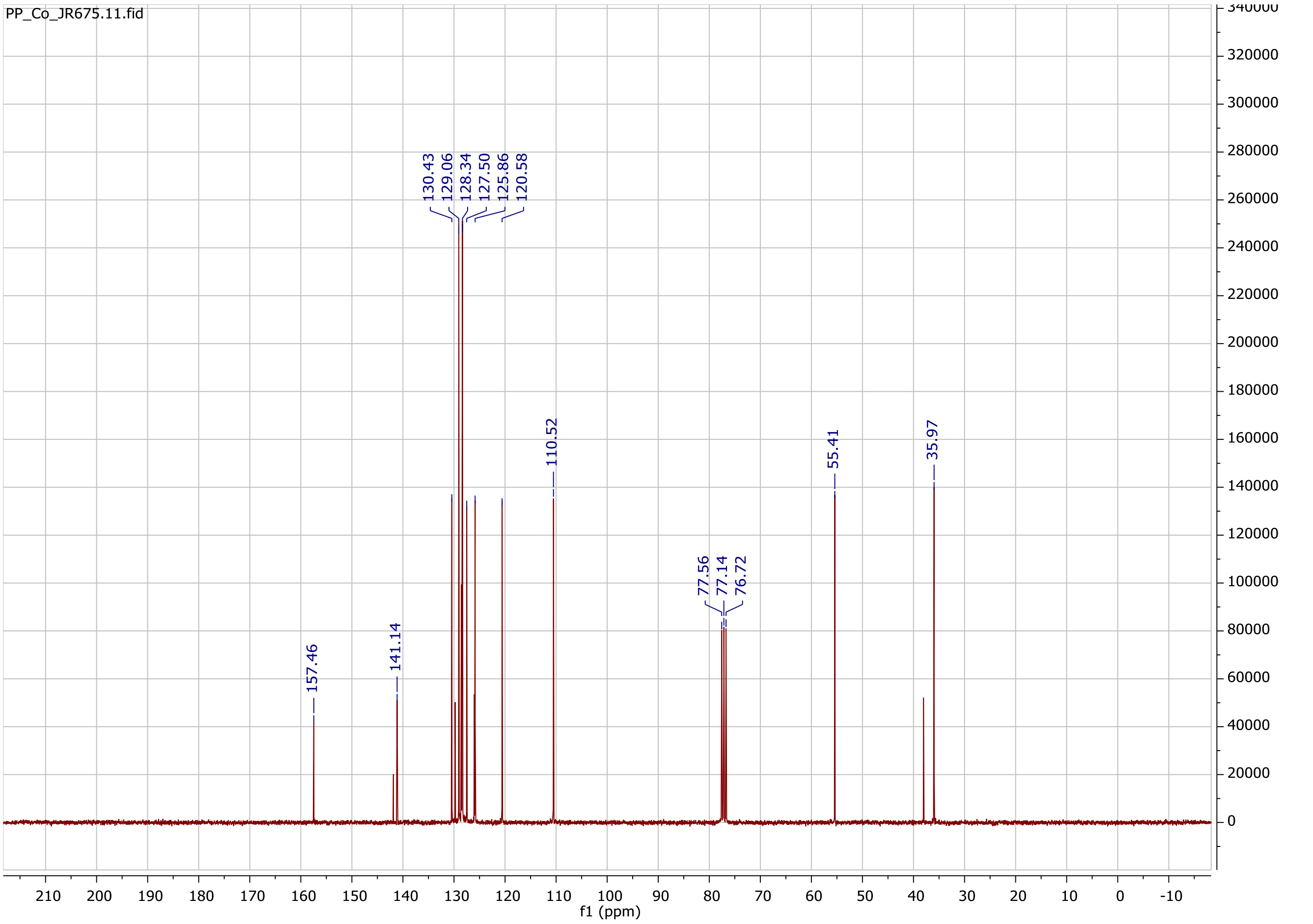
PP_Co_JR675.11.fid
130.43
129.06
128.34
127.50
125.86
120.58
110.52
157.46
141.14
77.56
77.14
76.72
55.41
35.97
f1 (ppm)

3g

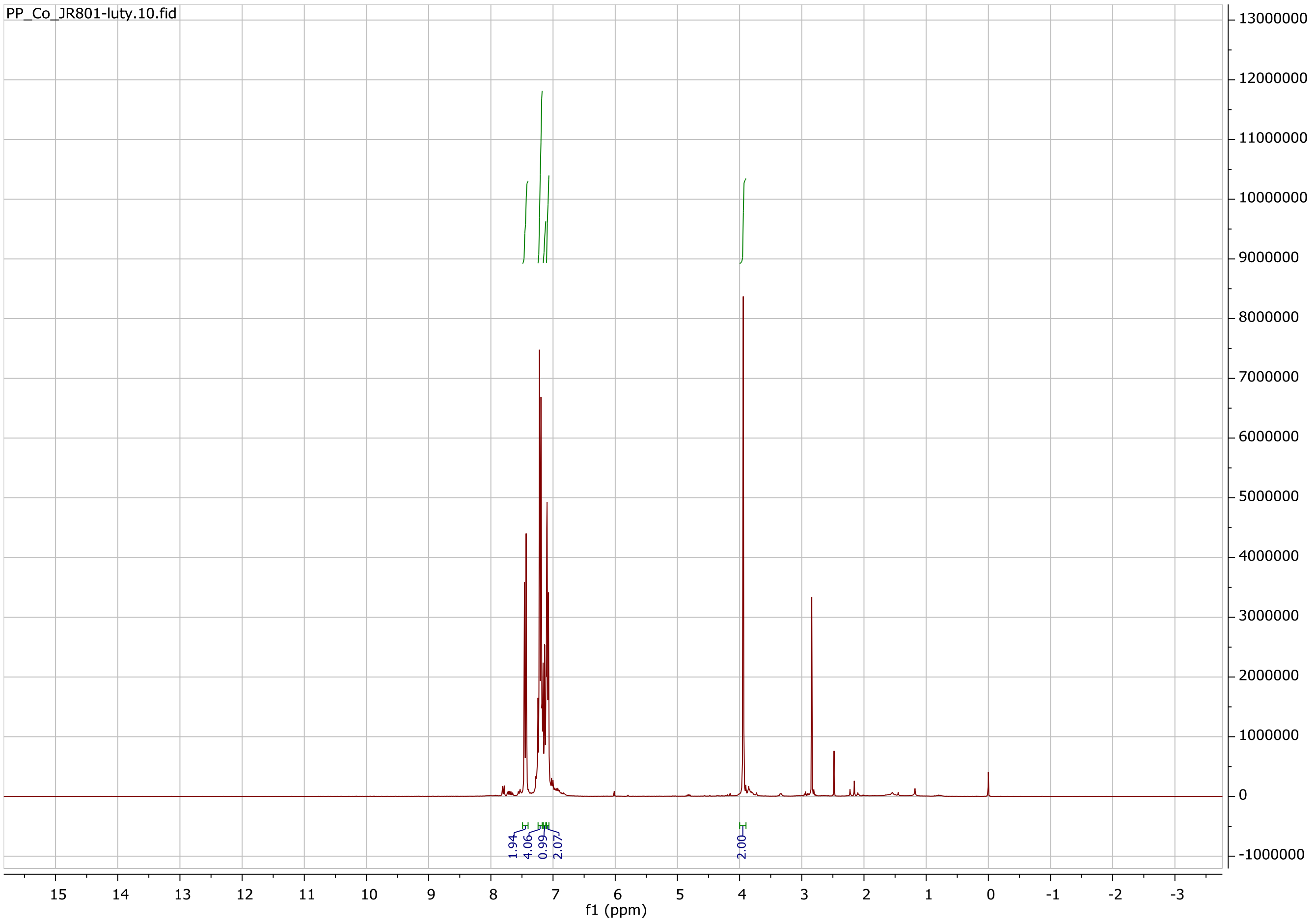
PP_Co_JR801-luty.10.fid
1.94
4.06
0.99
2.07
2.00
f1 (ppm)

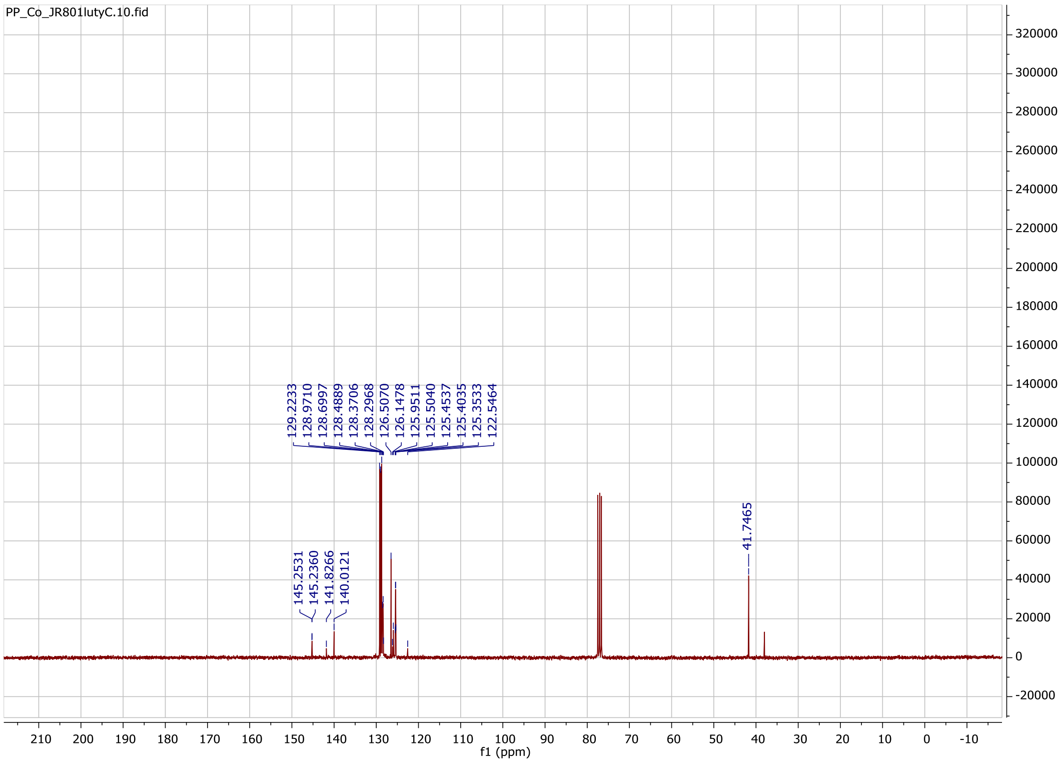
PP_Co_JR801lutyC.10.fid
129.2233
128.9710
128.6997
128.4889
128.3706
128.2968
126.5070
126.1478
125.9511
125.5040
125.4537
125.4035
125.3533
122.5464
145.2531
145.2360
141.8266
140.0121
41.7465
f1 (ppm)

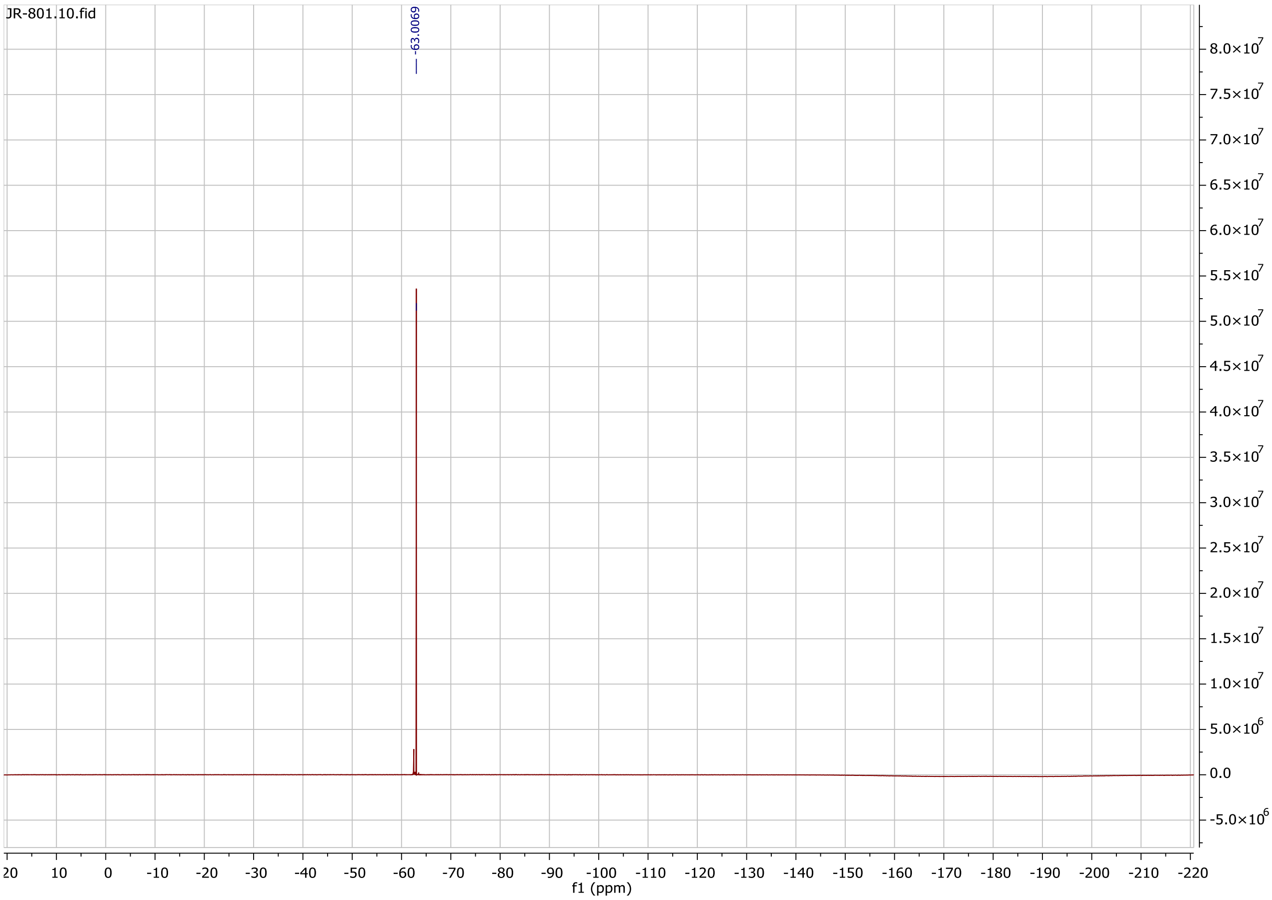
JR-801.10.fid
-63.0069
f1 (ppm)

3h

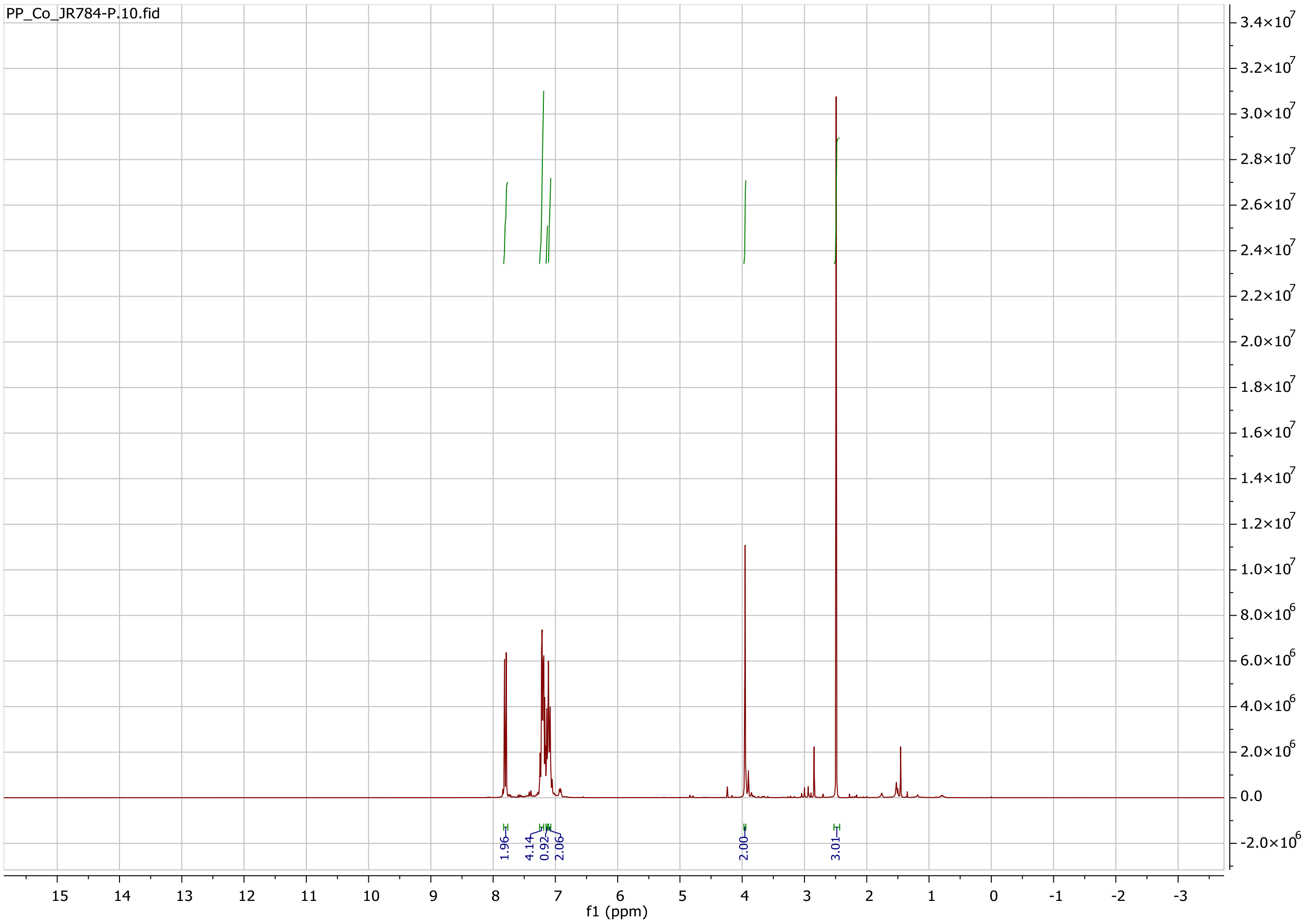
PP_Co_JR784-P.10.fid
1.96
4.14
0.92
2.06
2.00
3.01
f1 (ppm)

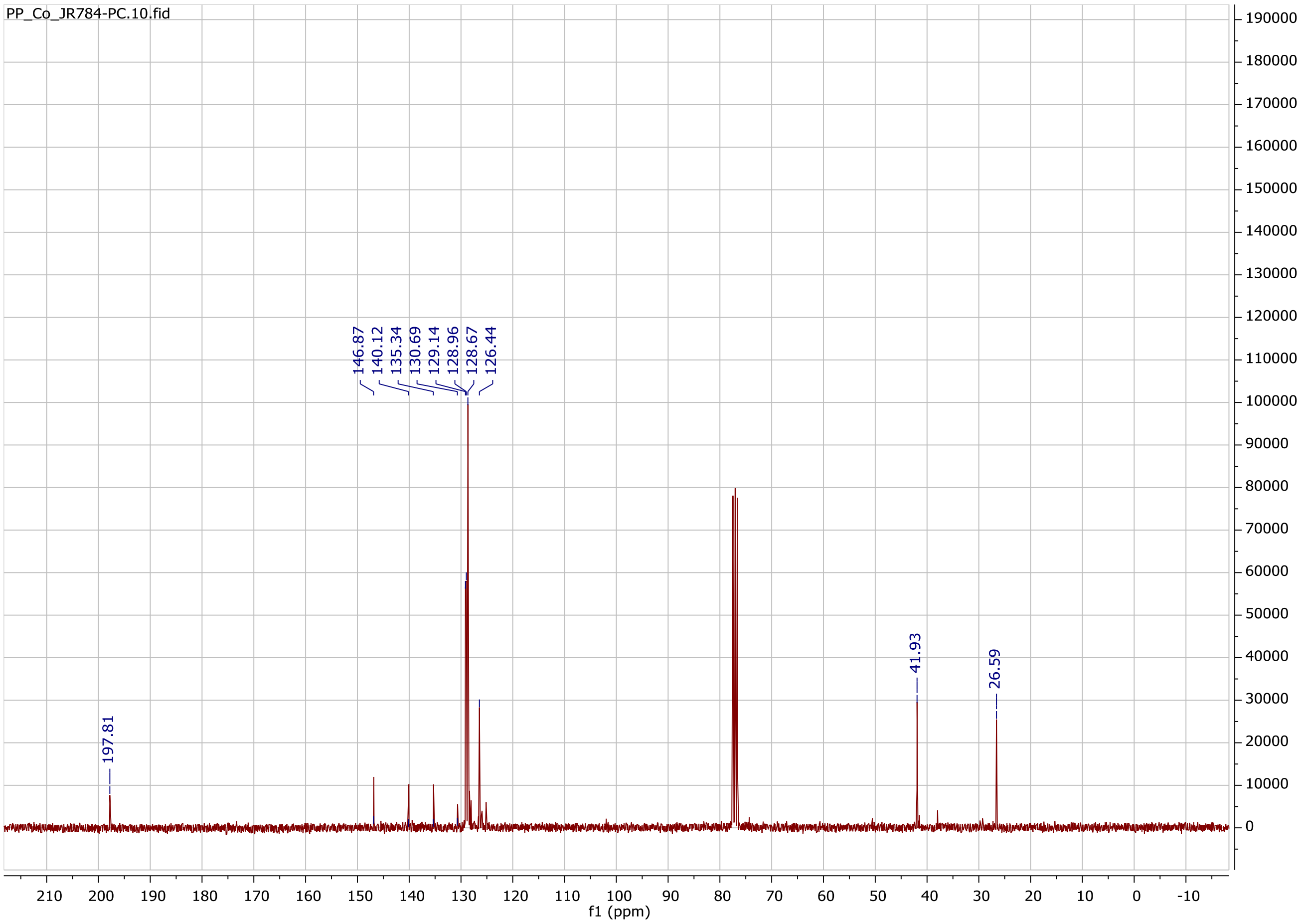
PP_Co_JR784-PC.10.fid
146.87
140.12
135.34
130.69
129.14
128.96
128.67
126.44
197.81
41.93
26.59
f1 (ppm)

3i

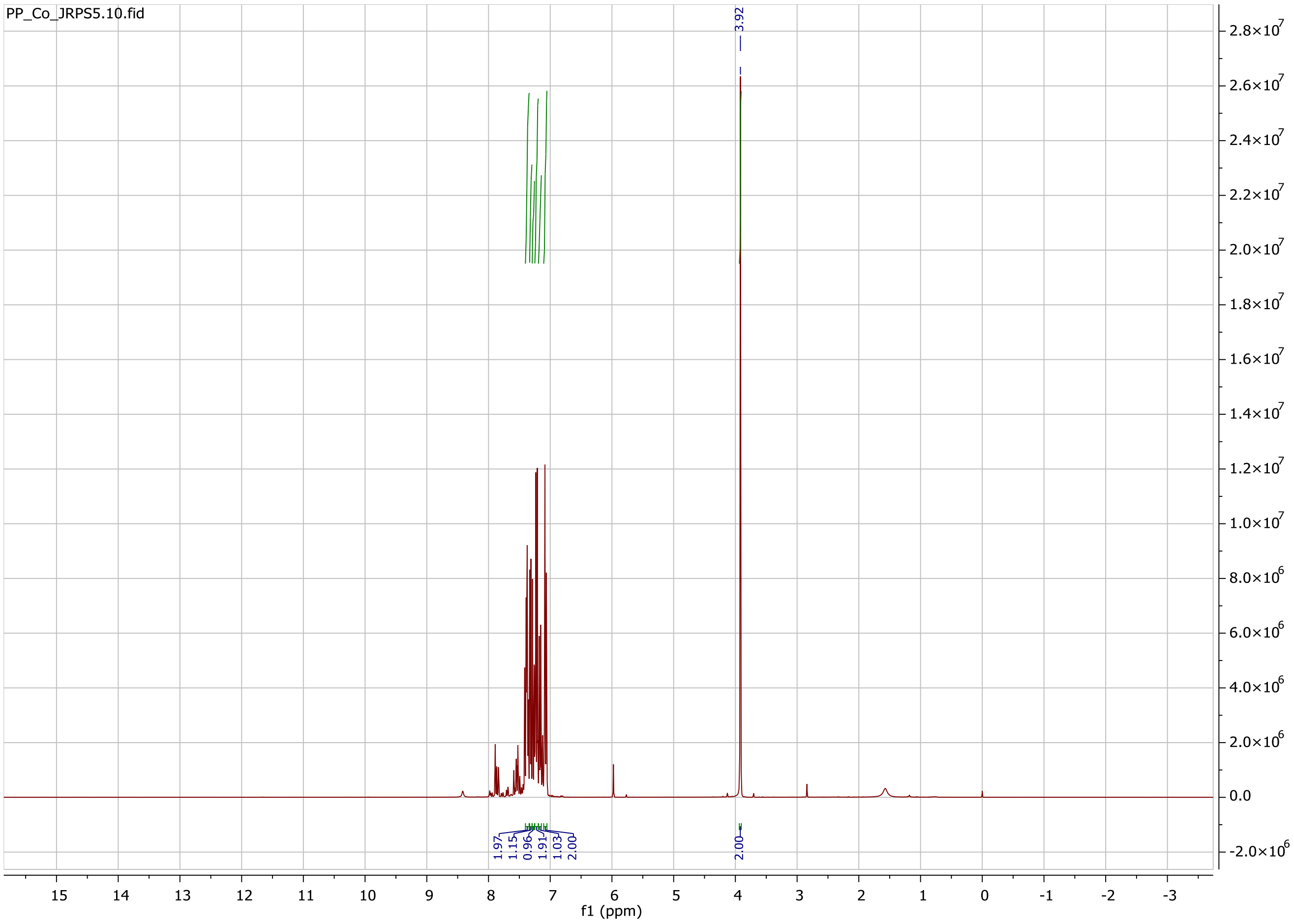
PP_Co_JRPS5.10.fid
3.92
1.97
1.15
0.96
1.91
1.03
2.00
2.00
f1 (ppm)

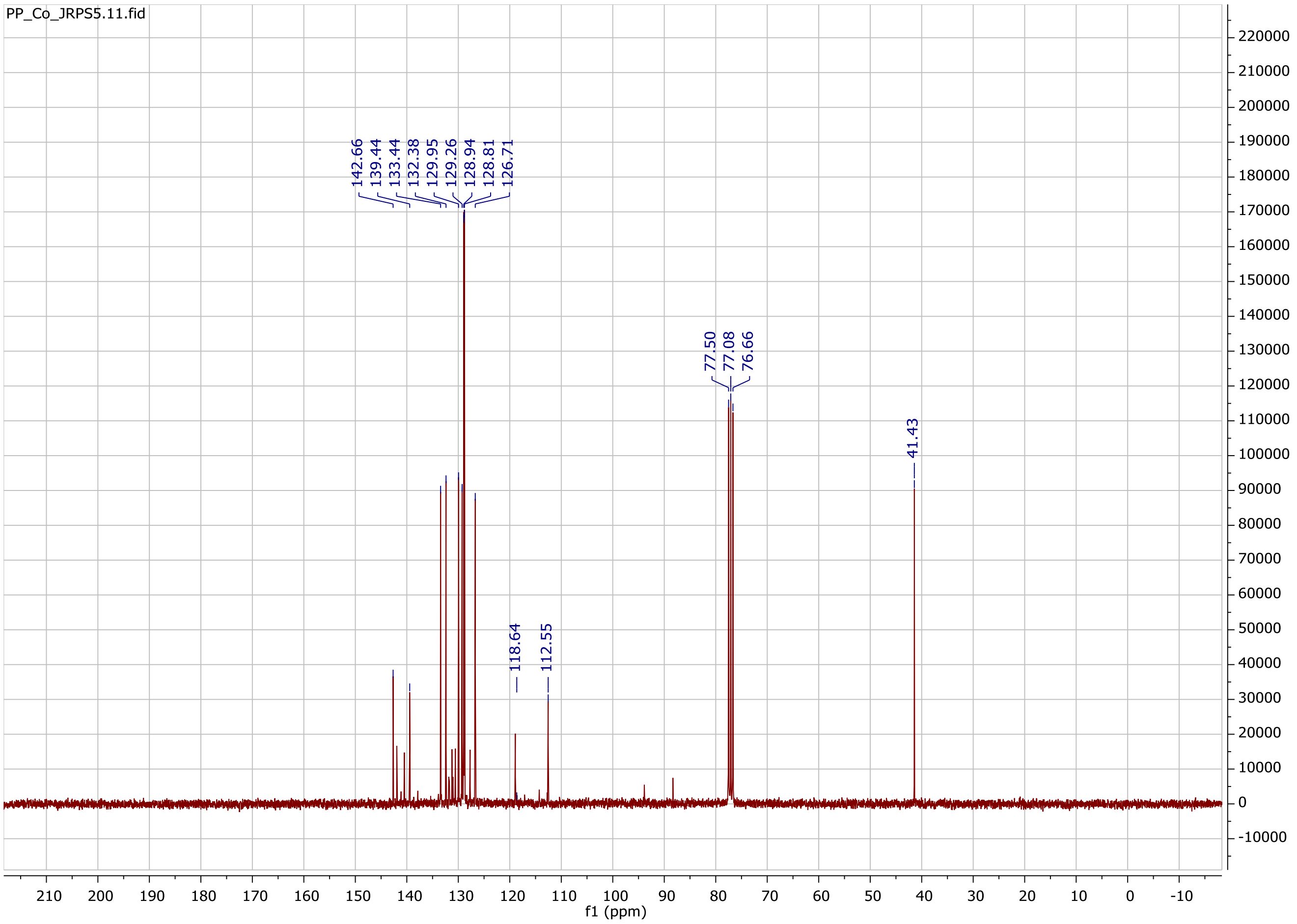
PP_Co_JRPS5.11.fid
142.66
139.44
133.44
132.38
129.95
129.26
128.94
128.81
126.71
77.50
77.08
76.66
41.43
118.64
112.55
f1 (ppm)

3j

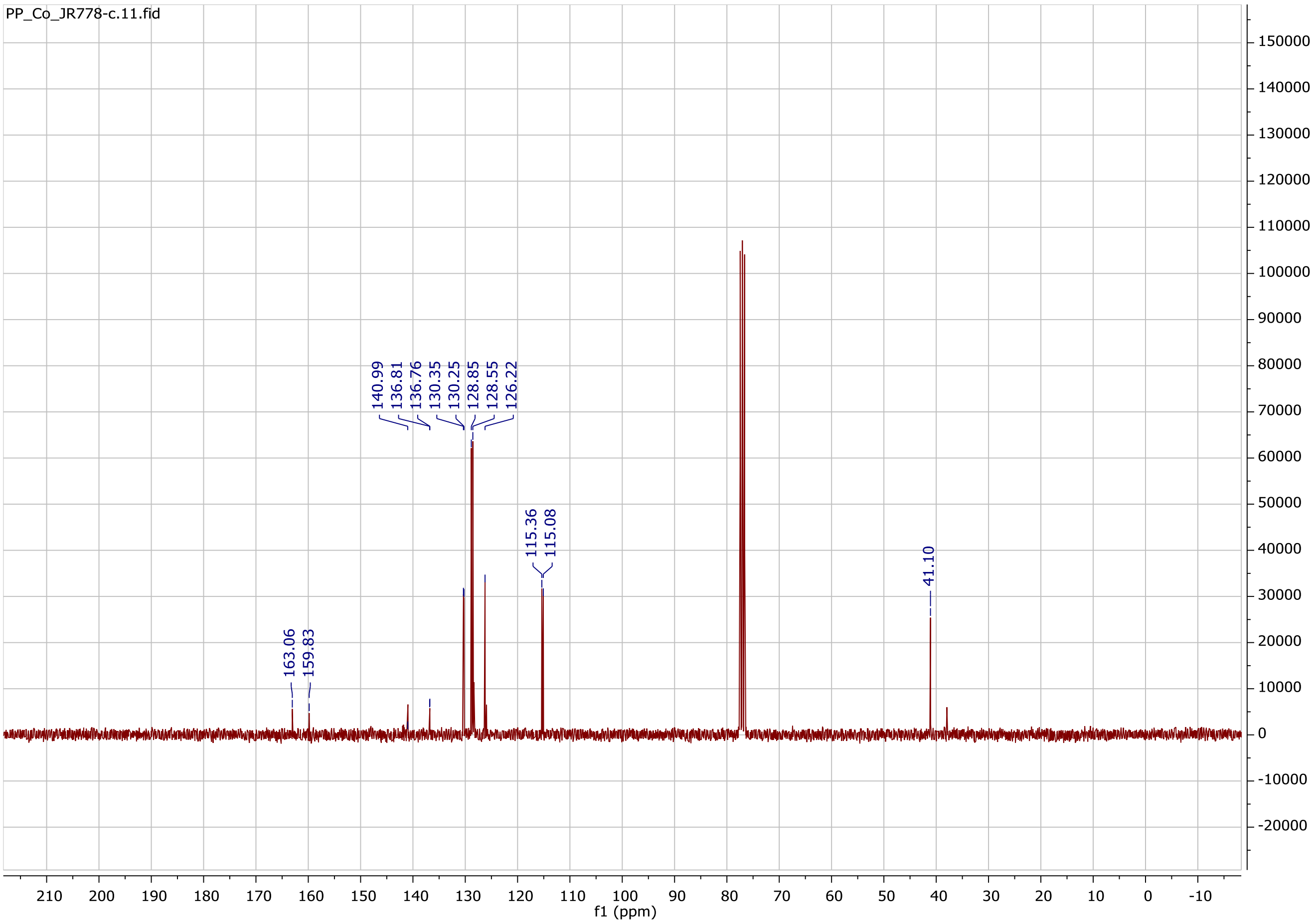
PP_Co_JR778-c.11.fid
140.99
136.81
136.76
130.35
130.25
128.85
128.55
126.22
115.36
115.08
163.06
159.83
41.10
f1 (ppm)

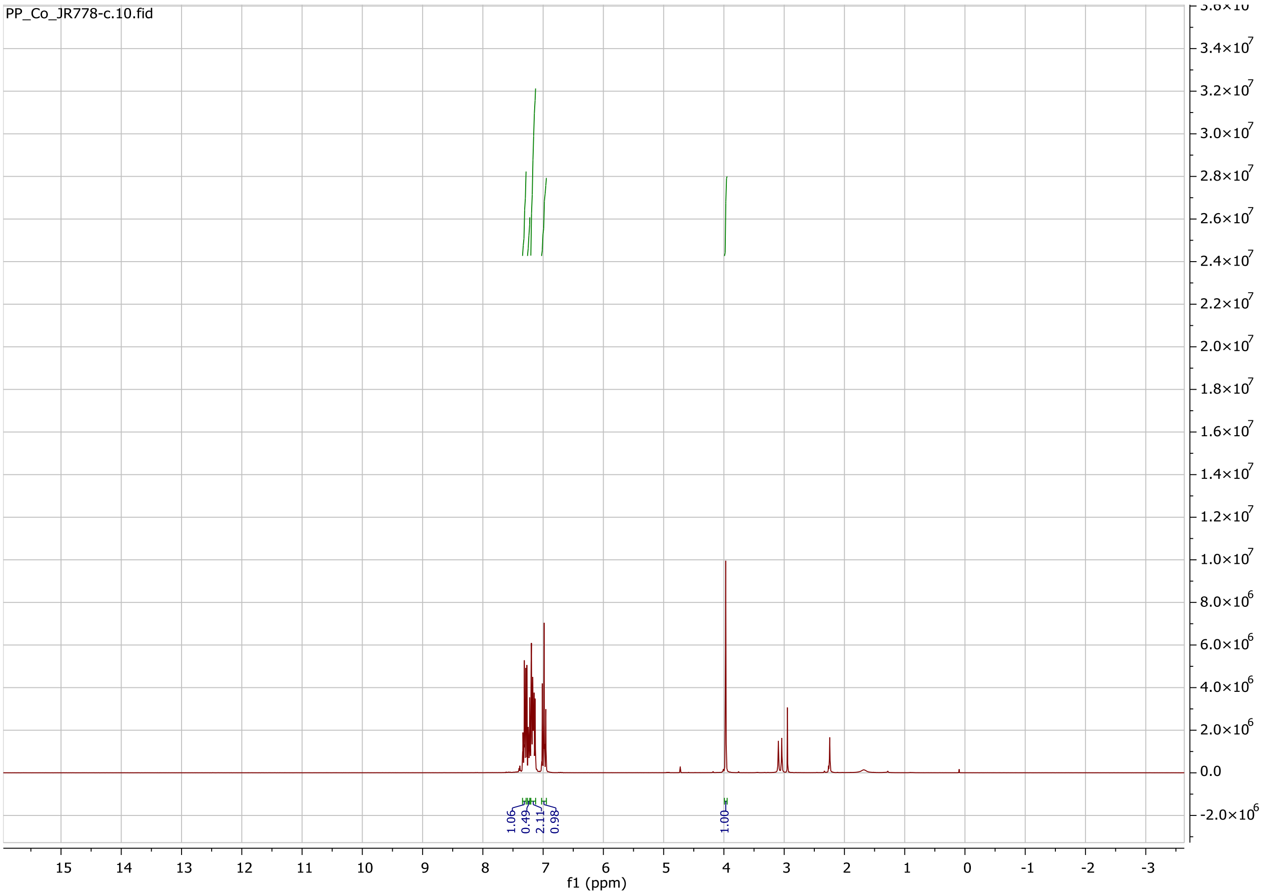
PP_Co_JR778-c.10.fid
1.06
0.49
2.11
0.98
1.00
f1 (ppm)

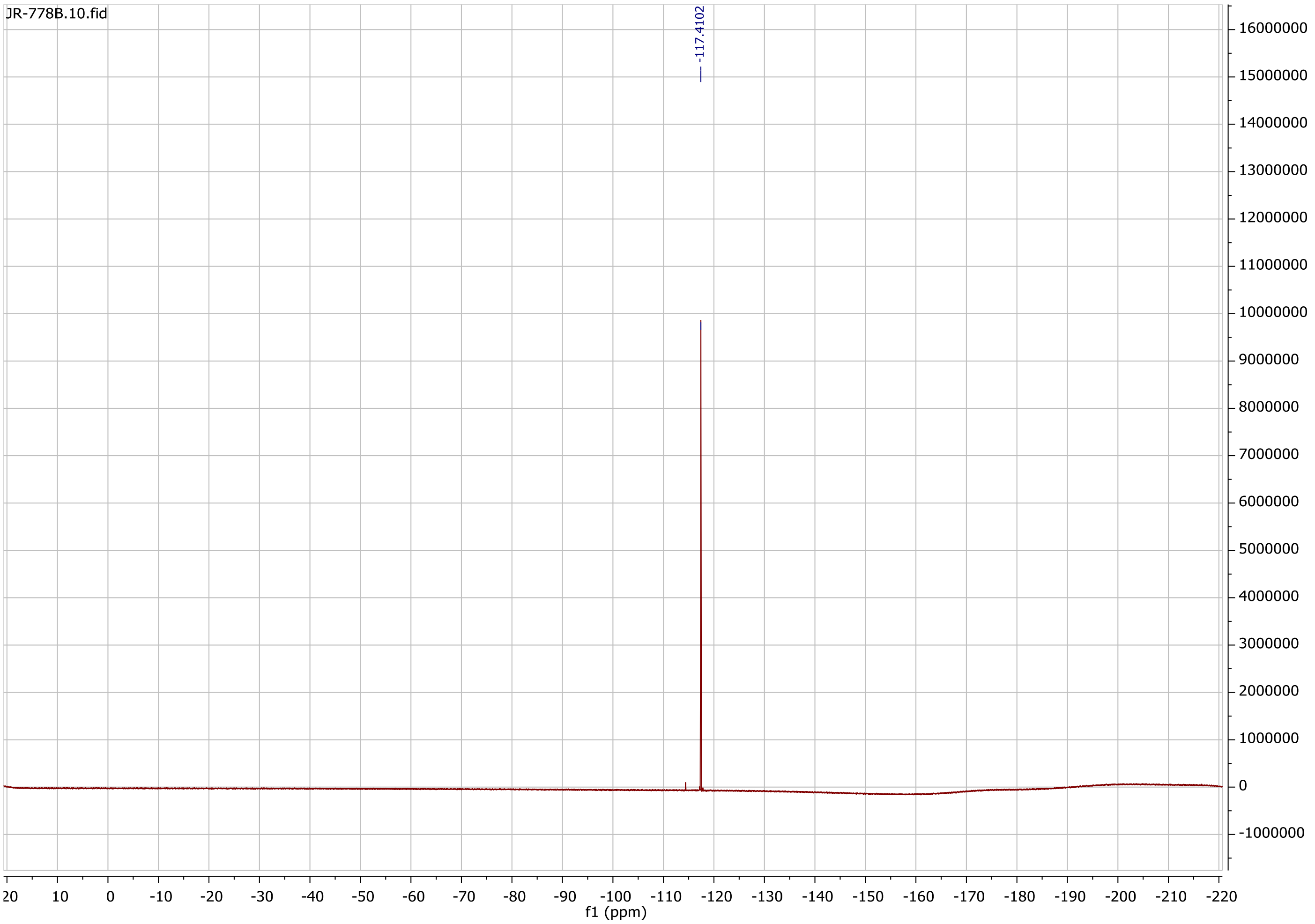
JR-778B.10.fid
-117.4102
f1 (ppm)

3k

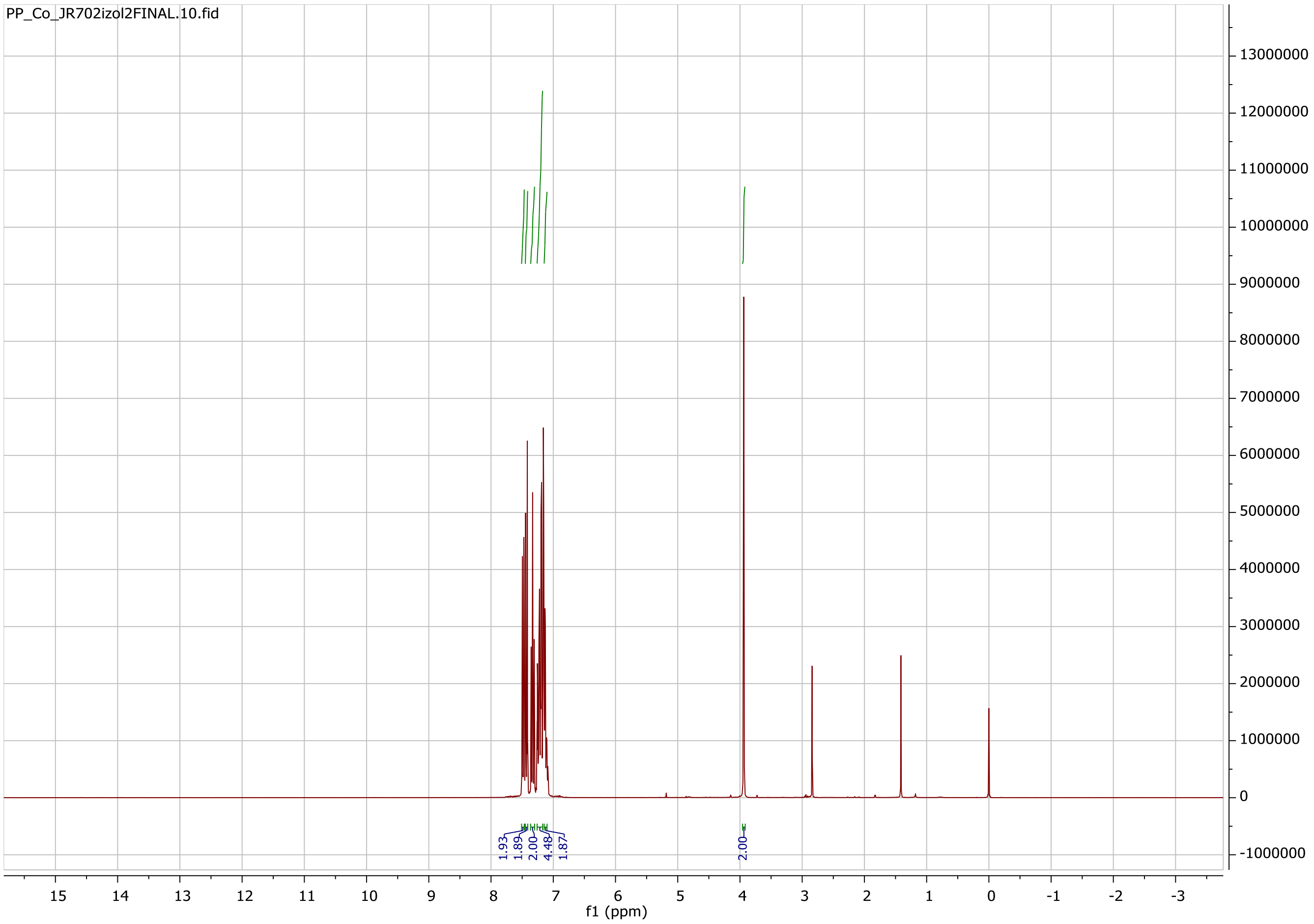

PP_Co_JR702izol2FINAL.10.fid
1.93
1.89
2.00
4.48
1.87
2.00
f1 (ppm)

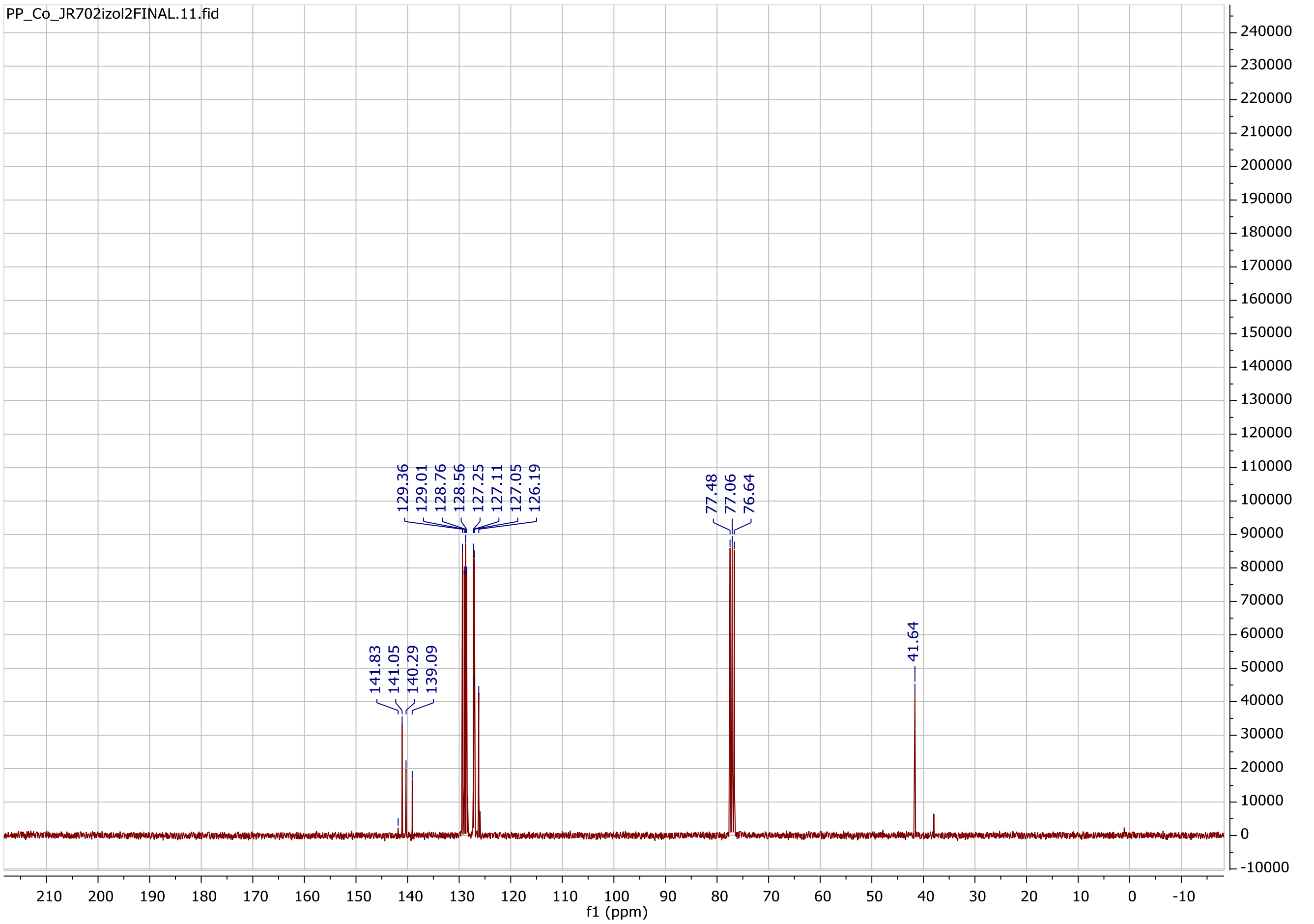

PP_Co_JR702izol2FINAL.11.fid
129.36
129.01
128.76
128.56
127.25
127.11
127.05
126.19
77.48
77.06
76.64
141.83
141.05
140.29
139.09
41.64
f1 (ppm)

3l

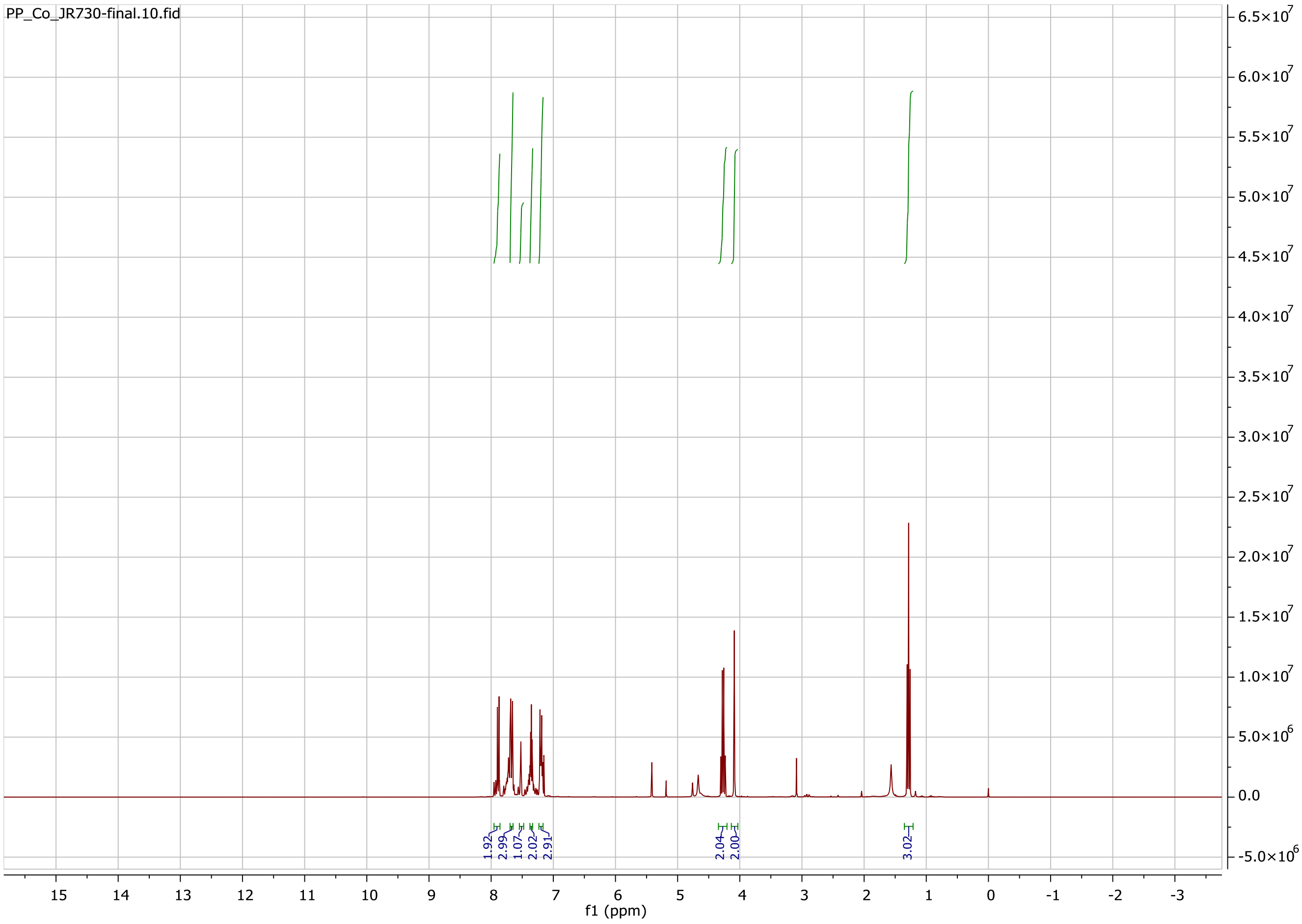
PP_Co_JR730-final.10.fid
1.92
2.99
1.07
2.02
2.91
2.04
2.00
3.02
f1 (ppm)

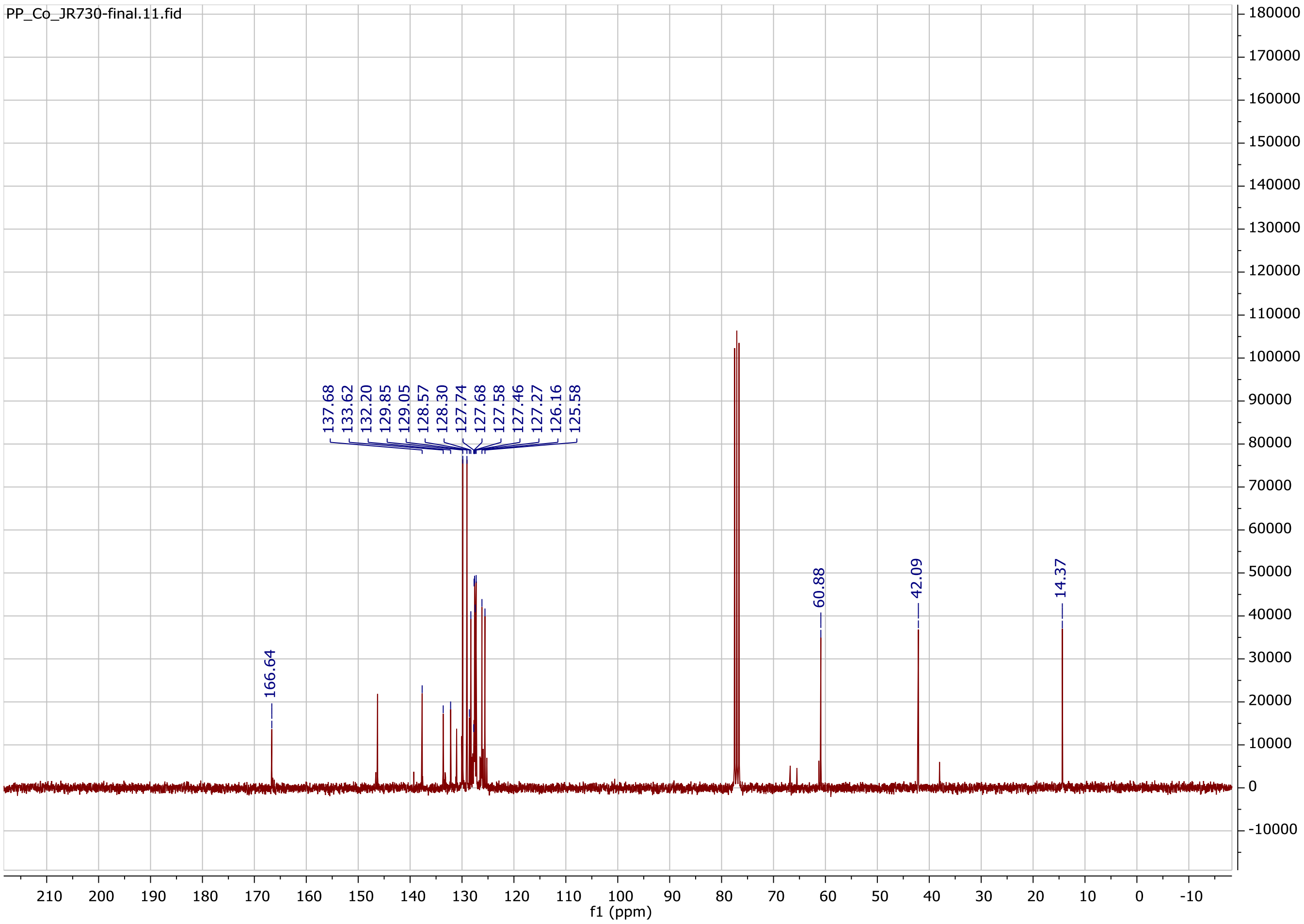
PP_Co_JR730-final.11.fid
137.68
133.62
132.20
129.85
129.05
128.57
128.30
127.74
127.68
127.58
127.46
127.27
126.16
125.58
166.64
60.88
42.09
14.37
f1 (ppm)

3m

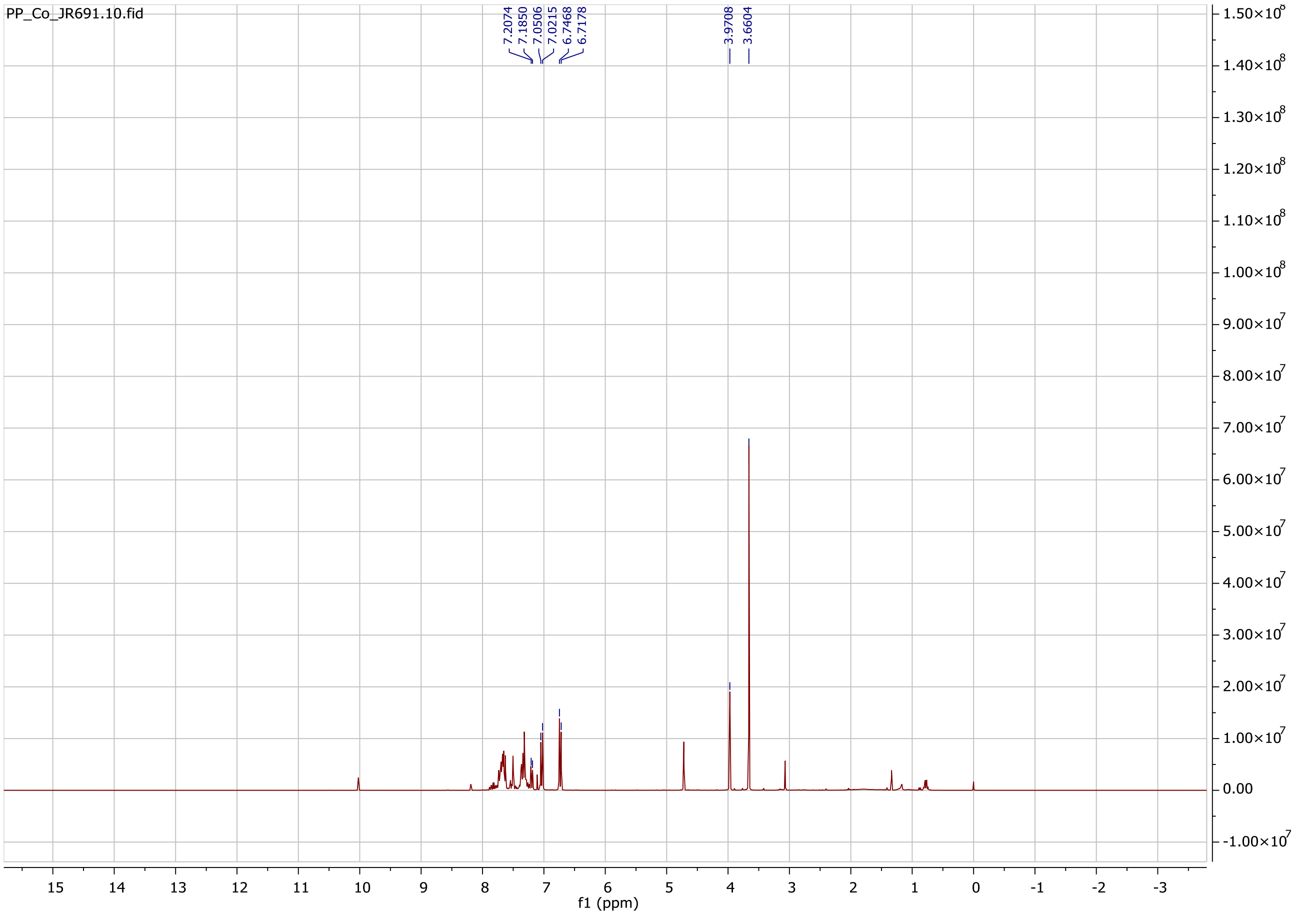
PP_Co_JR691.10.fid
7.2074
7.1850
7.0506
7.0215
6.7468
6.7178
3.9708
3.6604
f1 (ppm)

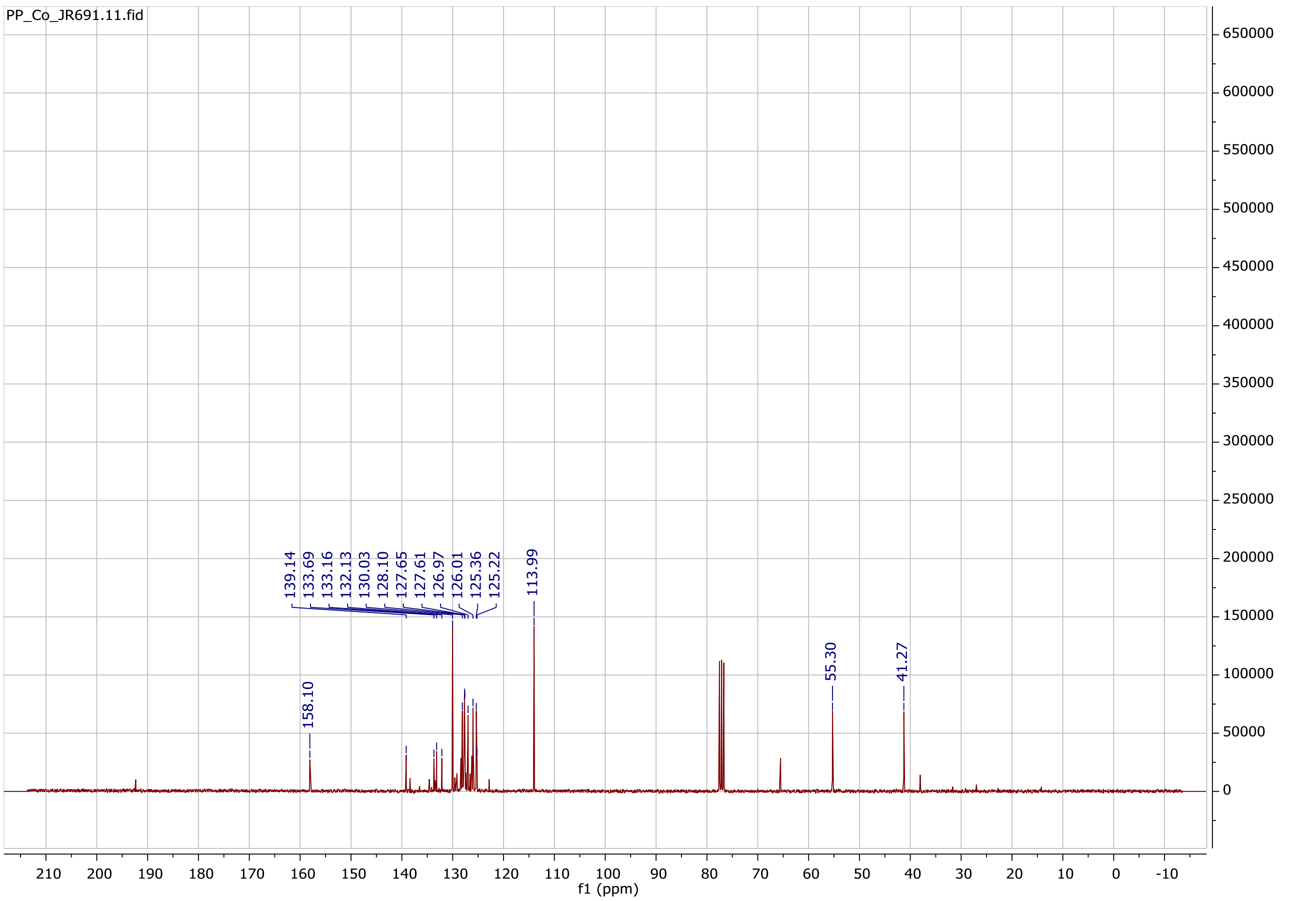
PP_Co_JR691.11.fid
158.10
139.14
133.69
133.16
132.13
130.03
128.10
127.65
127.61
126.97
126.01
125.36
125.22
113.99
55.30
41.27
f1 (ppm)

3n

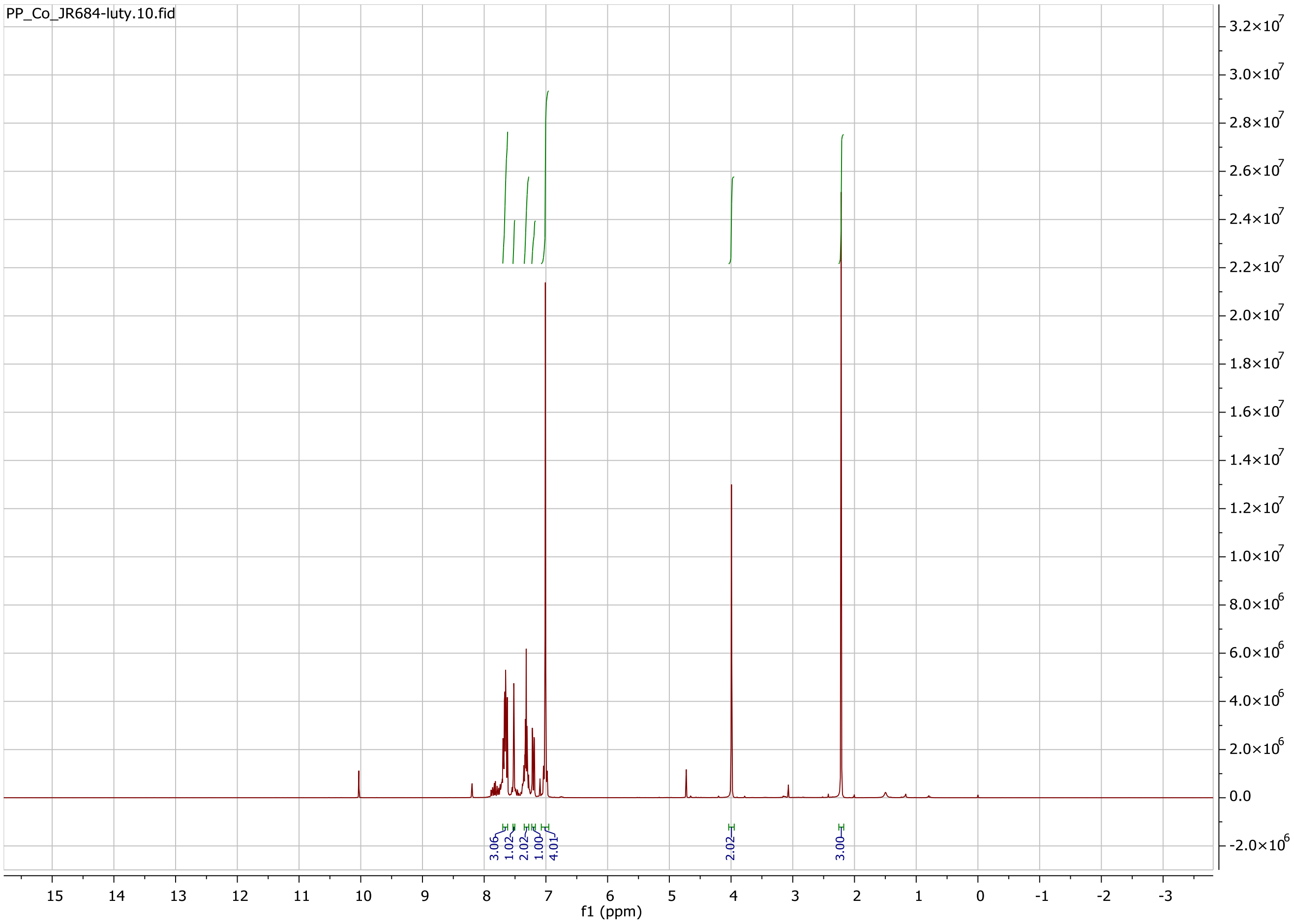
PP_Co_JR684-luty.10.fid
3.06
1.02
2.02
1.00
4.01
2.02
3.00
f1 (ppm)

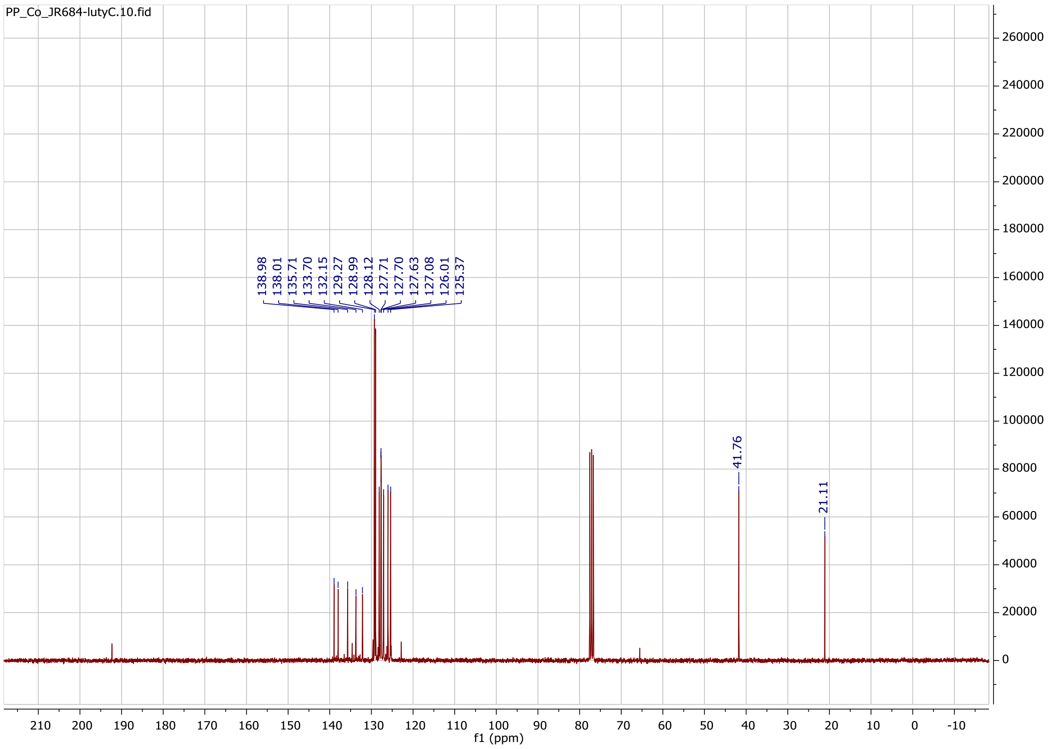
PP_Co_JR684-lutyC.10.fid
138.98
138.01
135.71
133.70
132.15
129.27
128.99
128.12
127.71
127.70
127.63
127.08
126.01
125.37
41.76
21.11
f1 (ppm)

3o

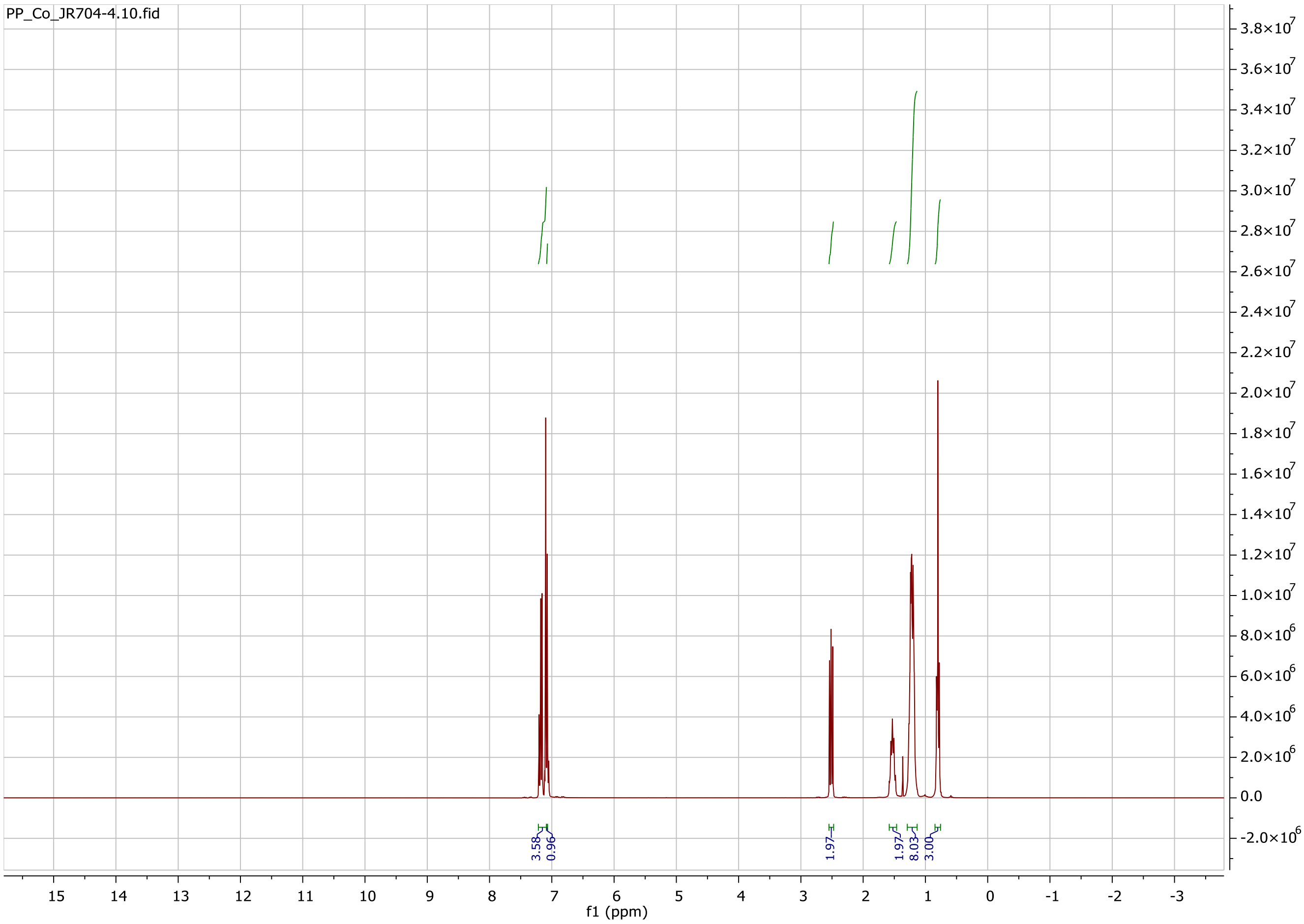
PP_Co_JR704-4.10.fid
f1 (ppm)
3.58
0.96
1.97
1.97
8.03
3.00

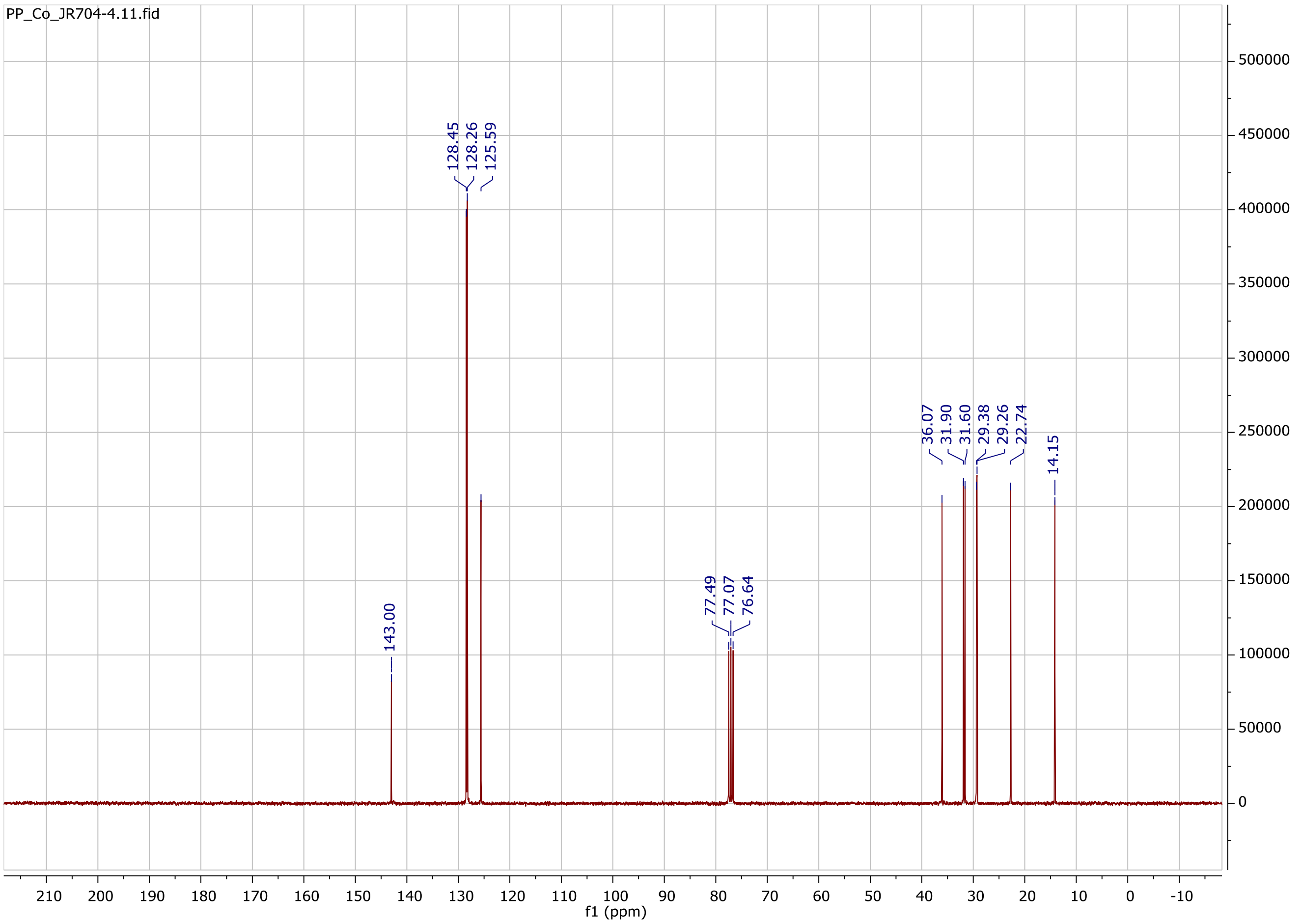
PP_Co_JR704-4.11.fid
f1 (ppm)
143.00
128.45
128.26
125.59
77.49
77.07
76.64
36.07
31.90
31.60
29.38
29.26
22.74
14.15

3q

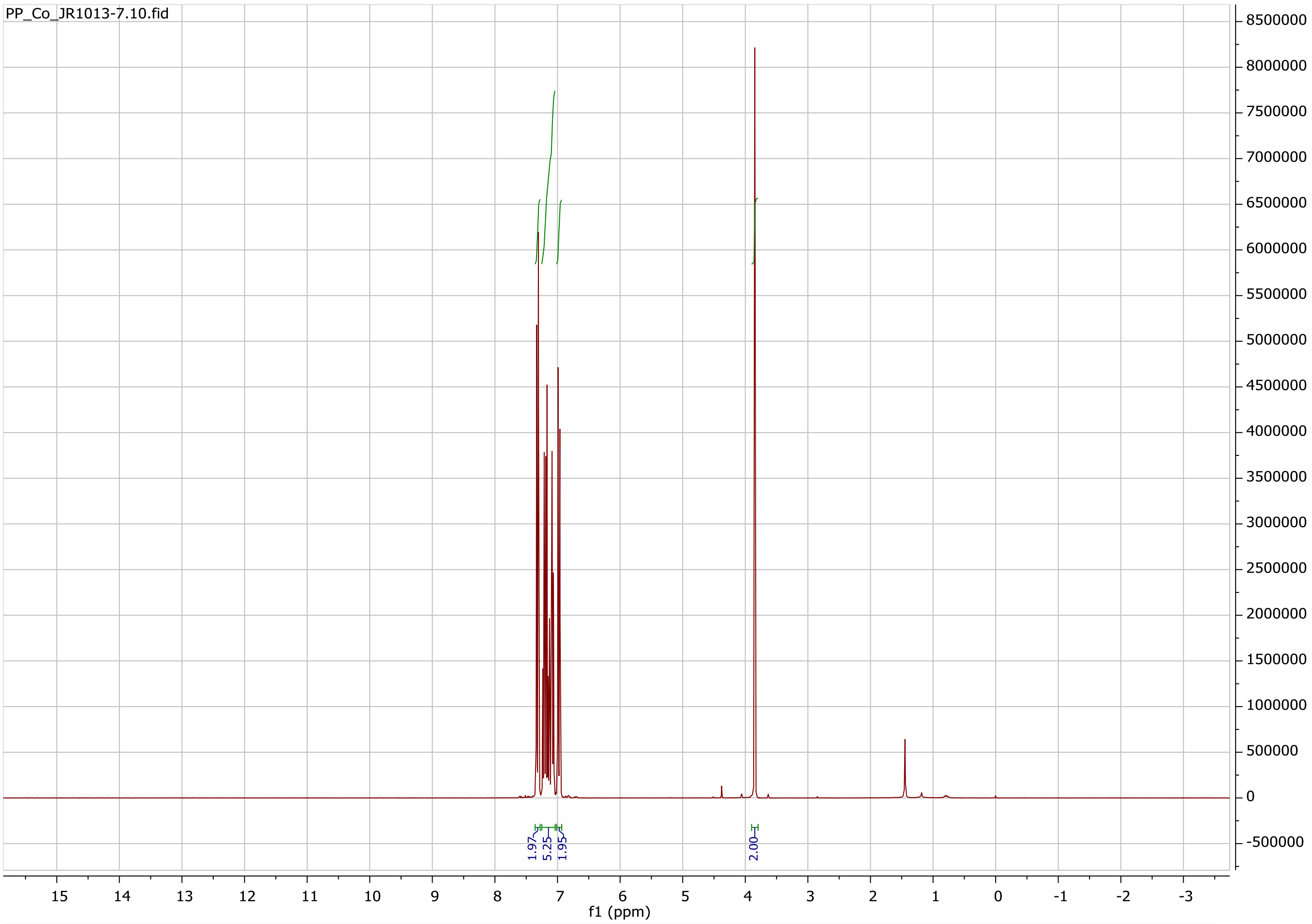
PP_Co_JR1013-7.10.fid
1.97
5.25
1.95
2.00
f1 (ppm)

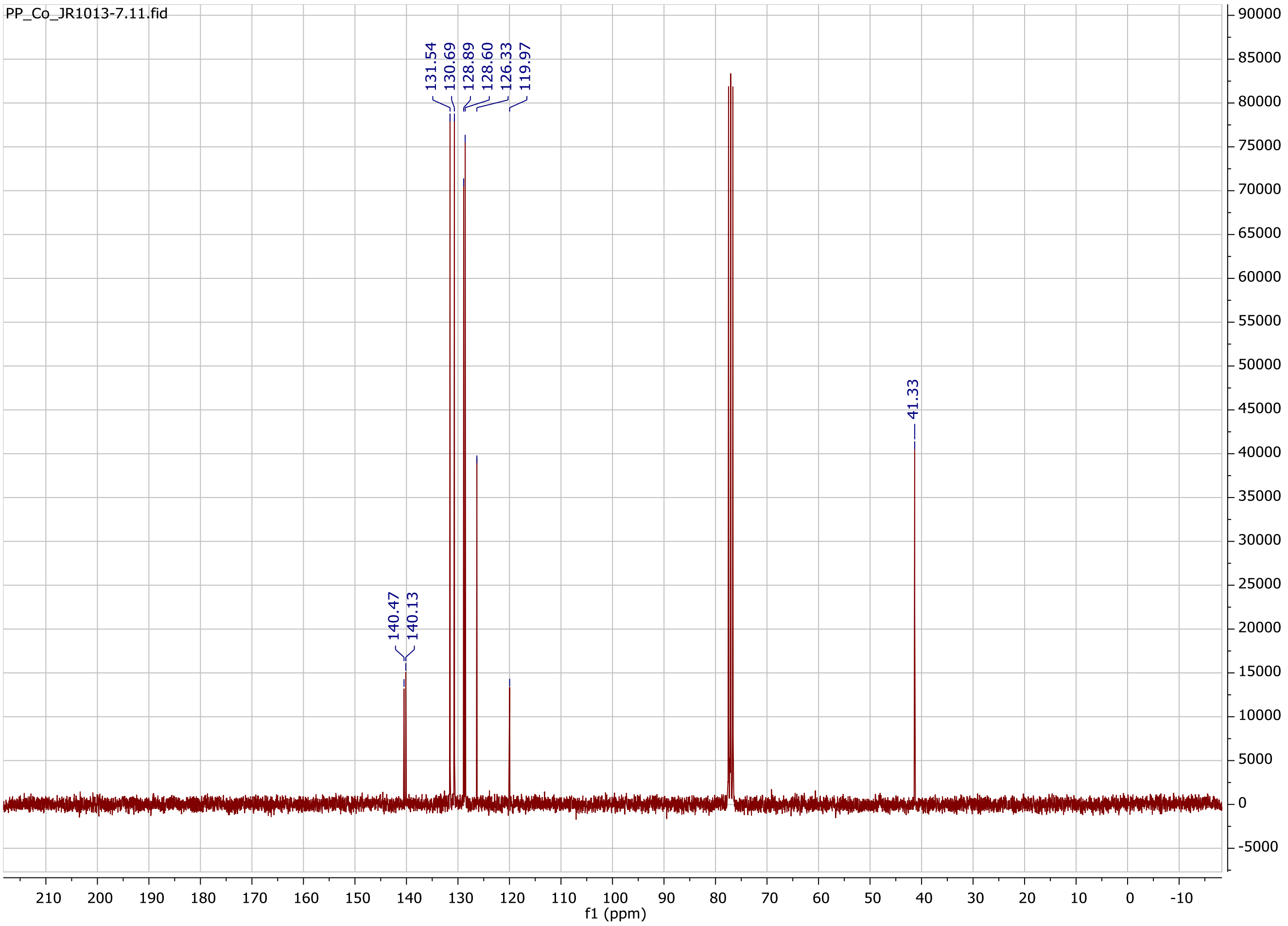
PP_Co_JR1013-7.11.fid
131.54
130.69
128.89
128.60
126.33
119.97
140.47
140.13
41.33
f1 (ppm)

3r

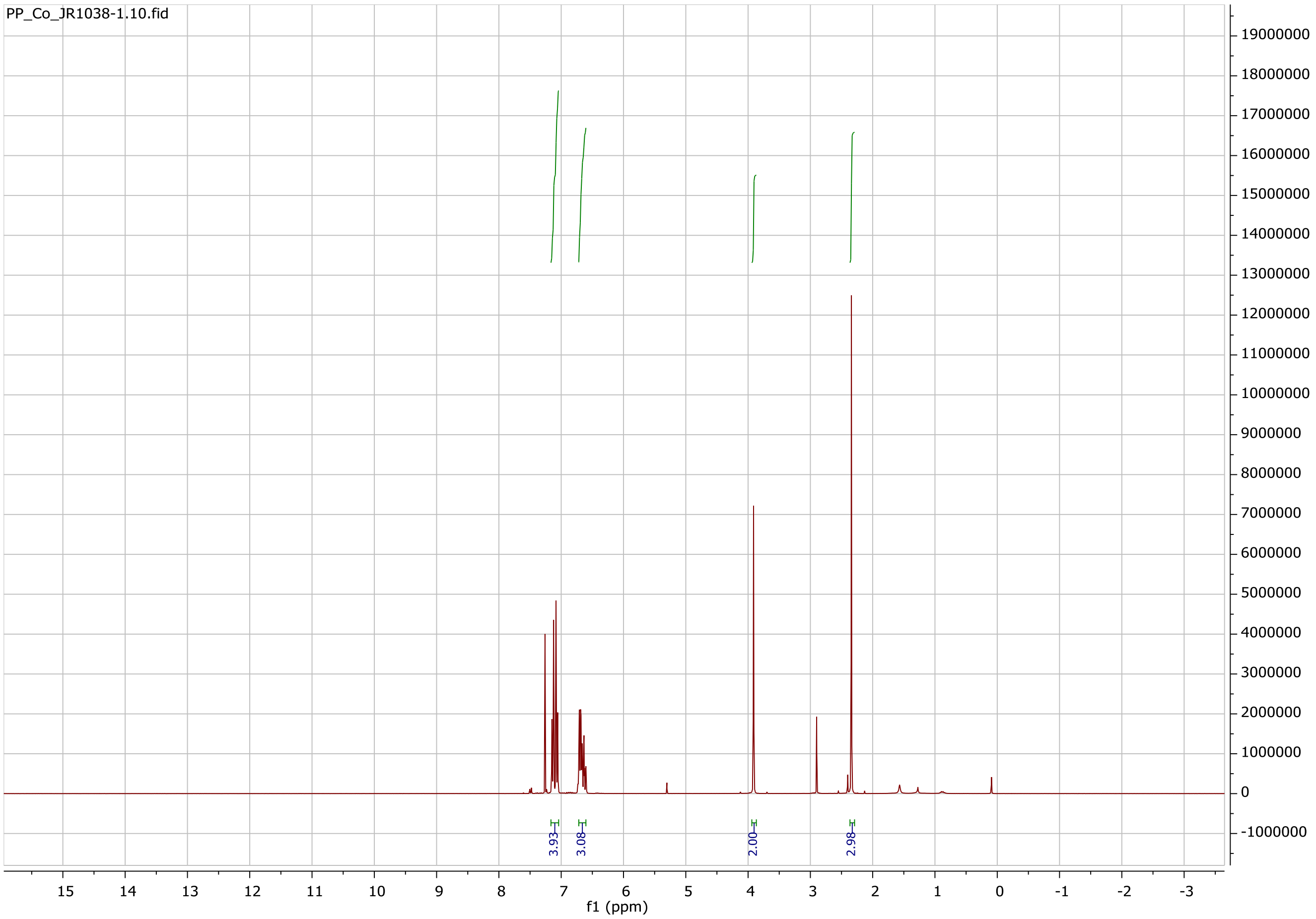
PP_Co_JR1038-1.10.fid
3.93
3.08
2.00
2.98
f1 (ppm)

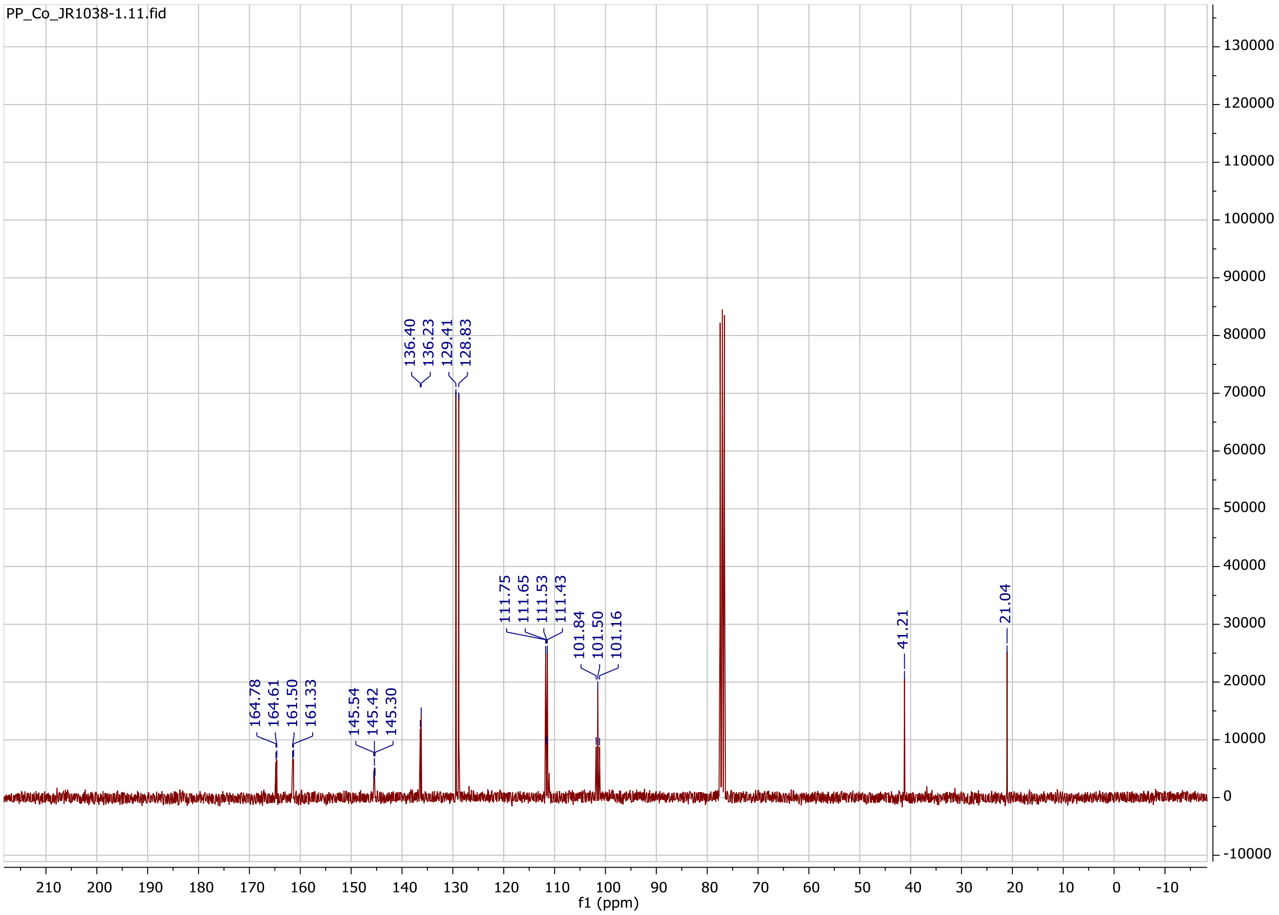
PP_Co_JR1038-1.11.fid
164.78
164.61
161.50
161.33
145.54
145.42
145.30
136.40
136.23
129.41
128.83
111.75
111.65
111.53
111.43
101.84
101.50
101.16
41.21
21.04
f1 (ppm)

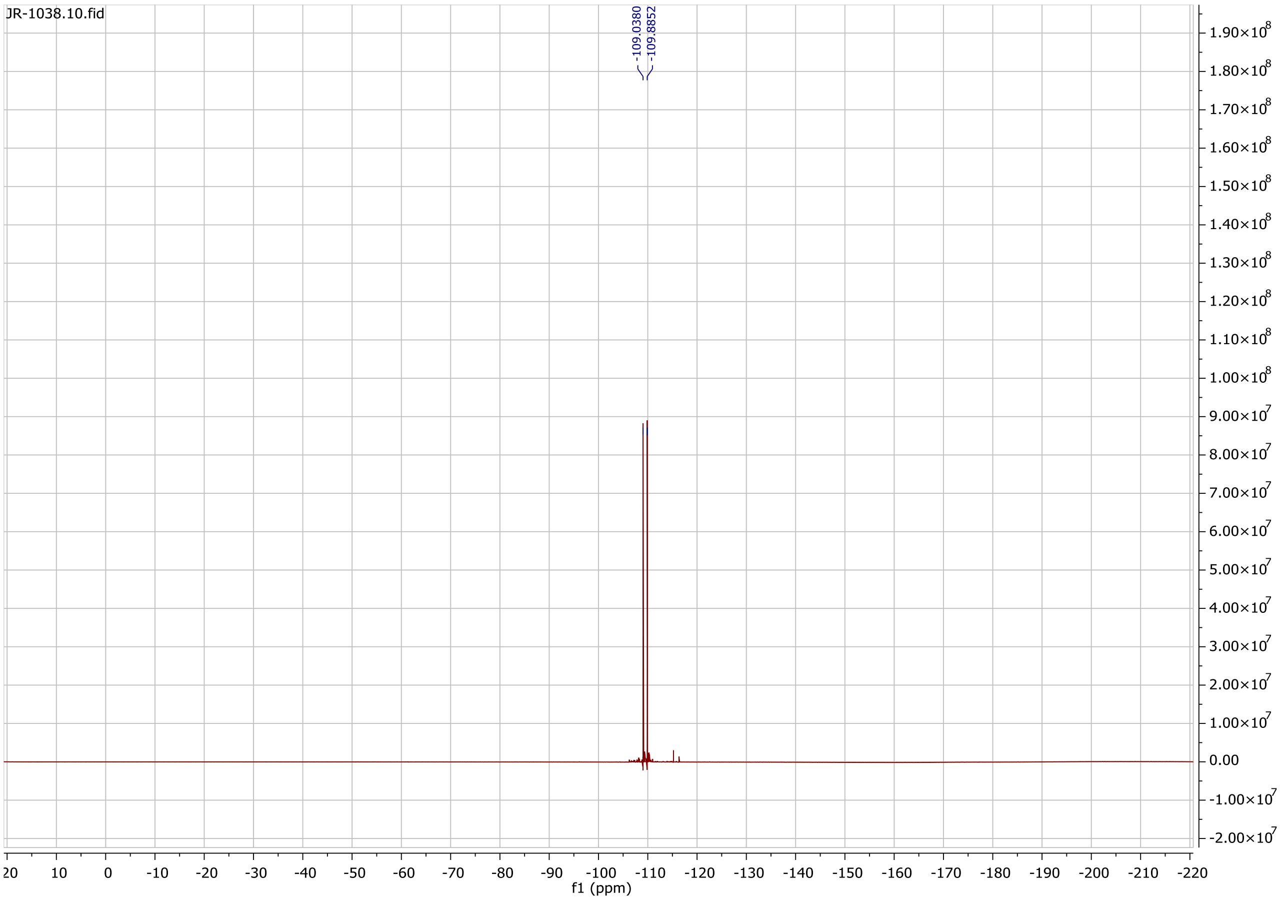
JR-1038.10.fid
-109.0380
-109.8852
f1 (ppm)

3s

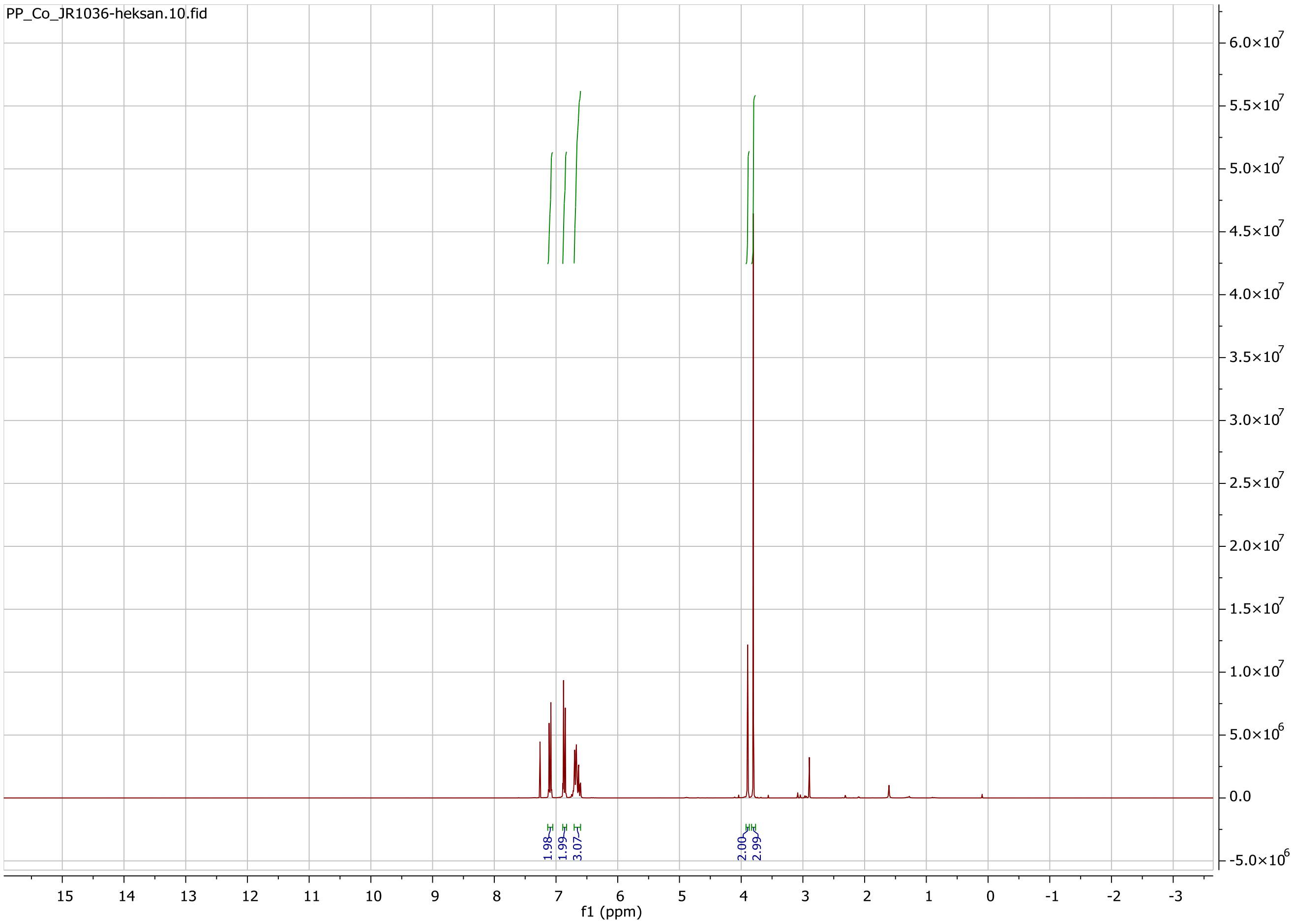
PP_Co_JR1036-heksan.10.fid
1.98
1.99
3.07
2.00
2.99
f1 (ppm)

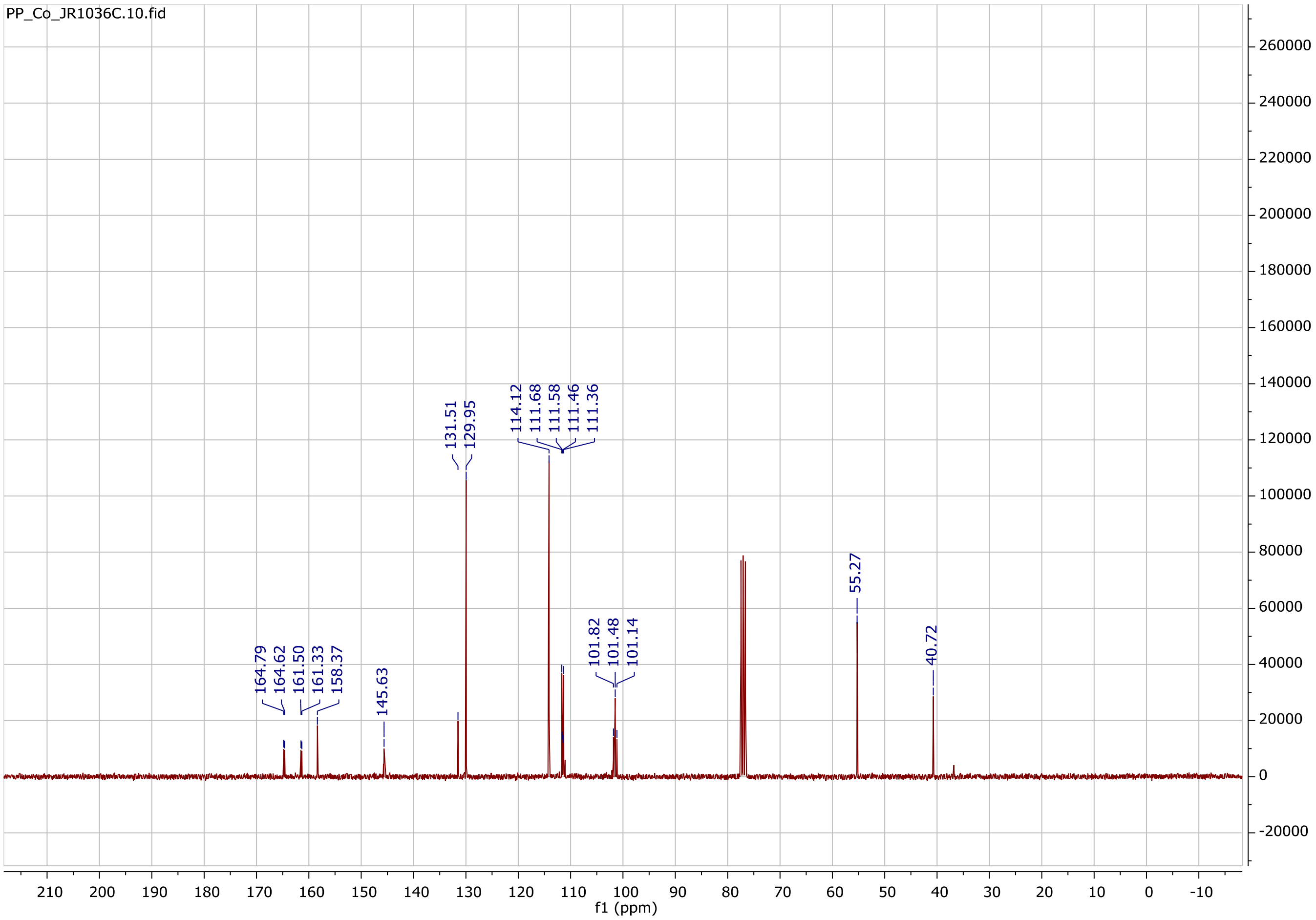
PP_Co_JR1036C.10.fid
164.79
164.62
161.50
161.33
158.37
145.63
131.51
129.95
114.12
111.68
111.58
111.46
111.36
101.82
101.48
101.14
55.27
40.72
f1 (ppm)

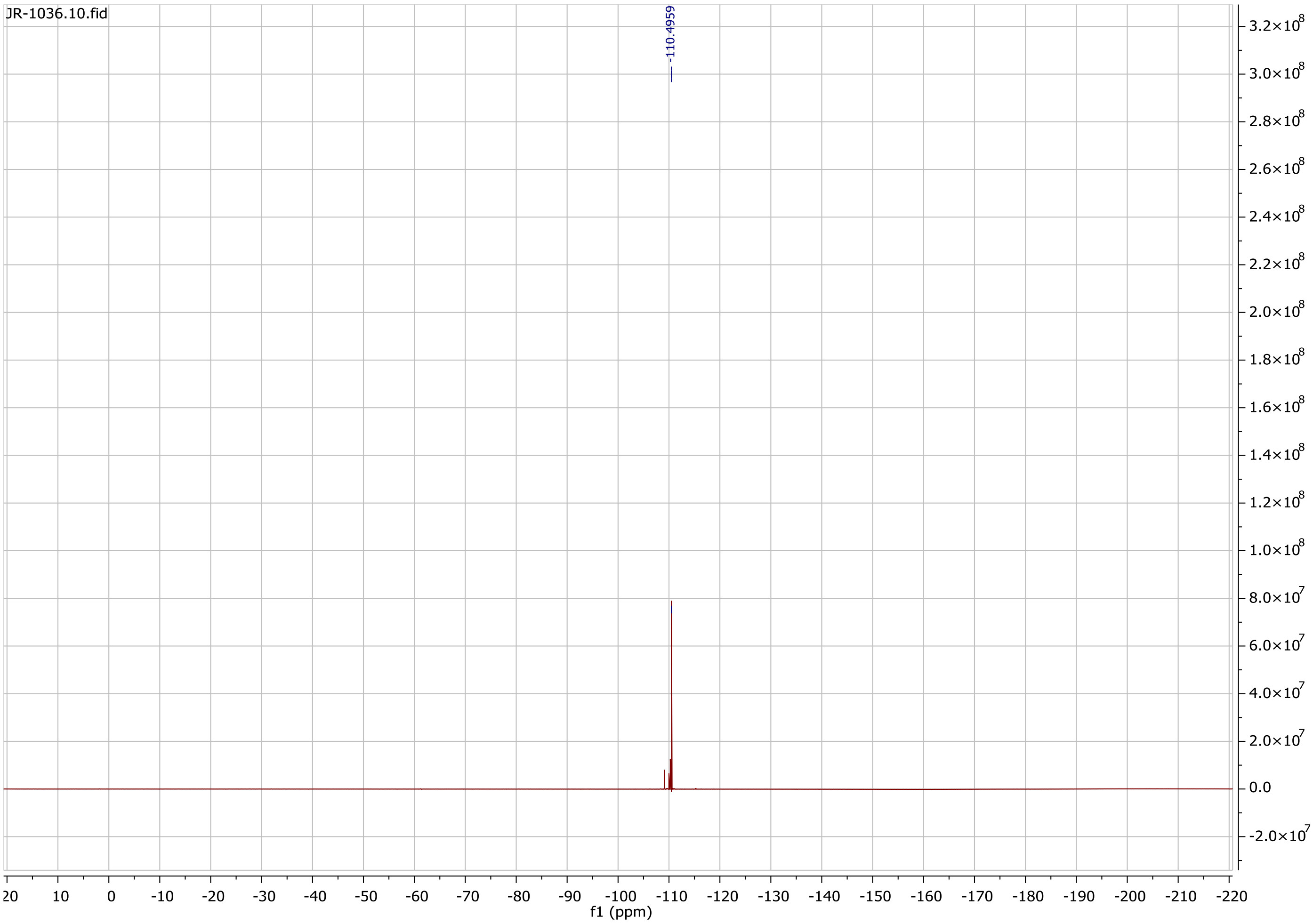
JR-1036.10.fid
-110.4959
f1 (ppm)

3t

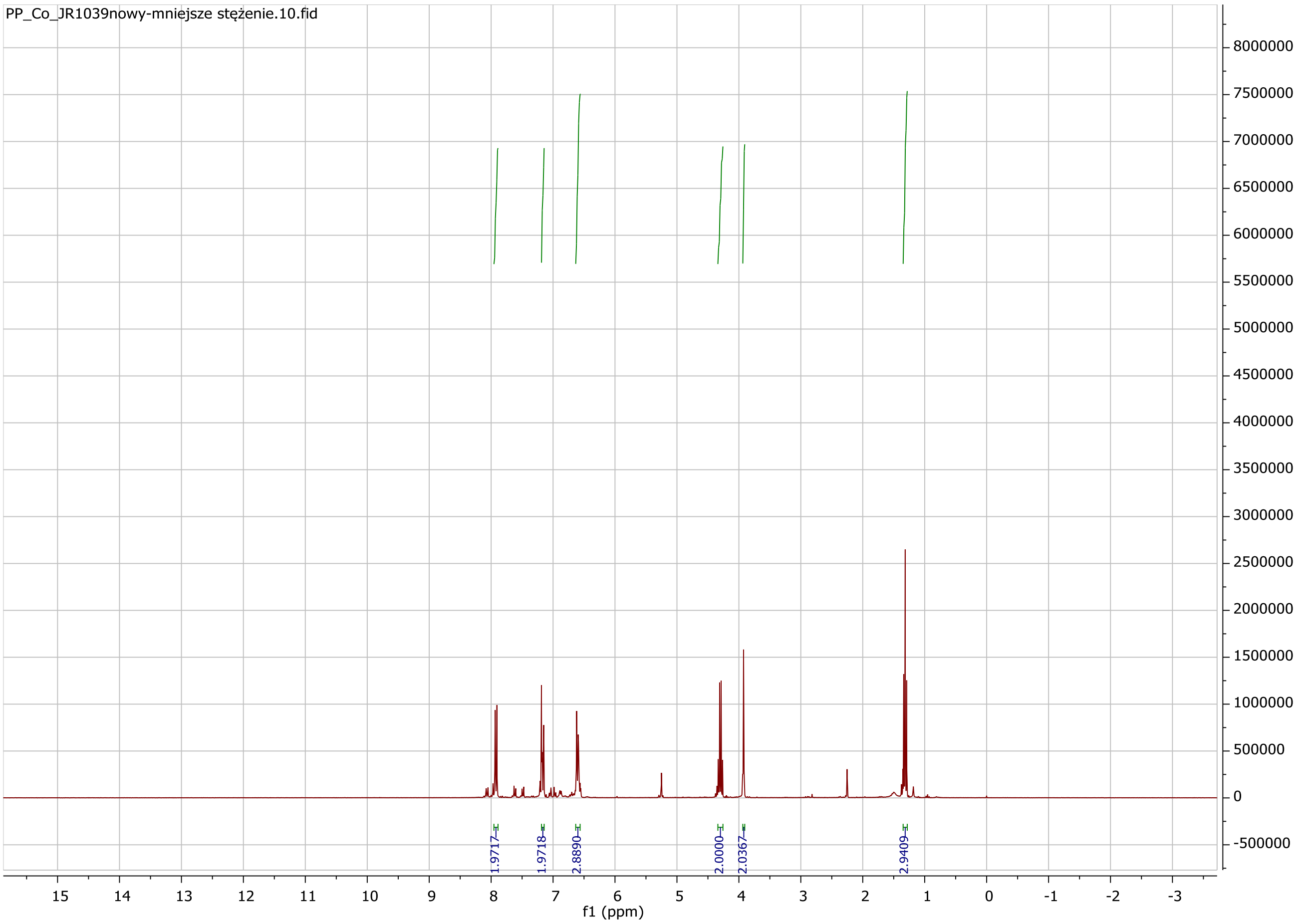

PP_Co_JR1039nowy-mniejsze stężenie.10.fid
1.9717
1.9718
2.8890
2.0000
2.0367
2.9409
f1 (ppm)

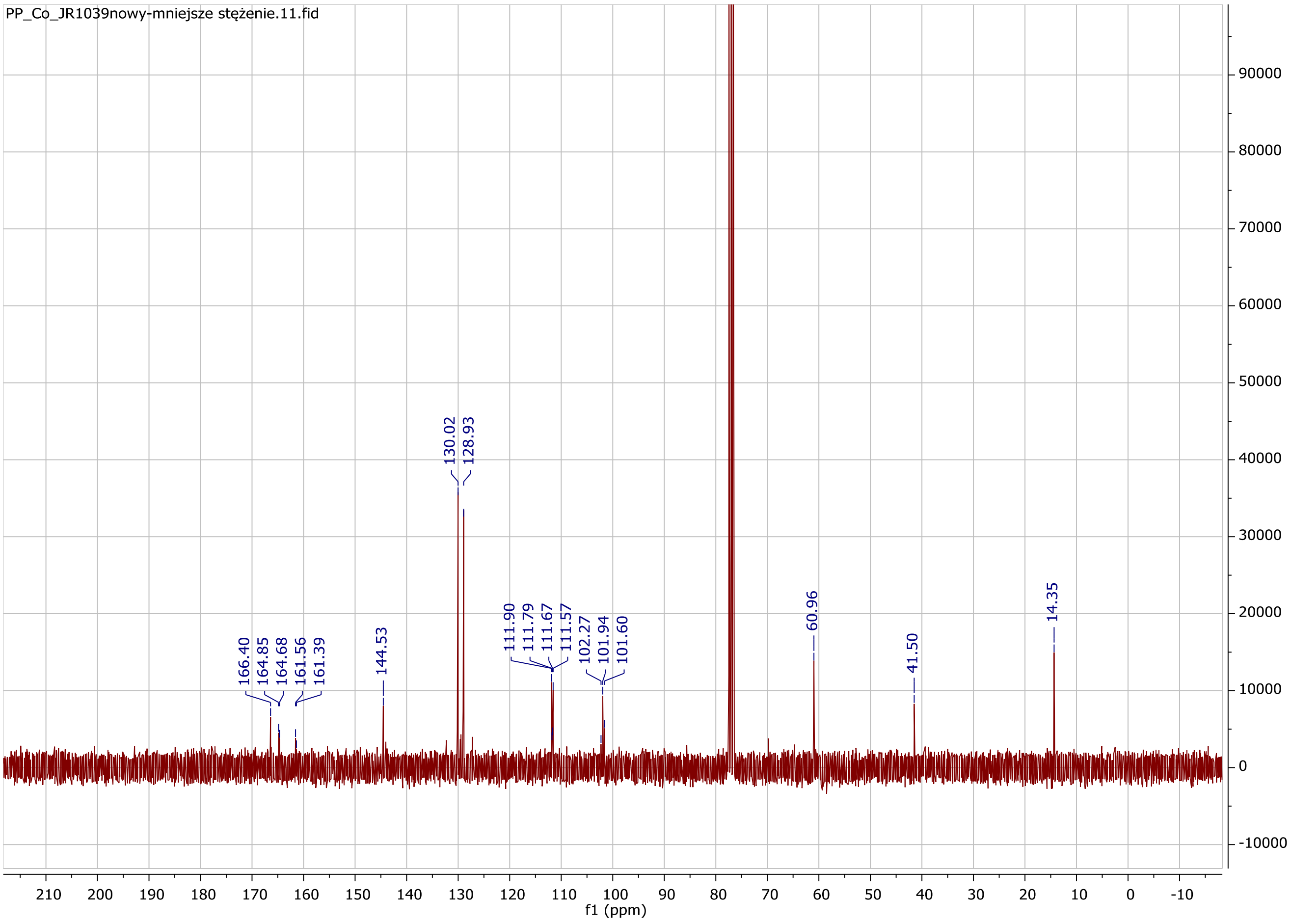

PP_Co_JR1039nowy-mniejsze stężenie.11.fid
166.40
164.85
164.68
161.56
161.39
144.53
130.02
128.93
111.90
111.79
111.67
111.57
102.27
101.94
101.60
60.96
41.50
14.35
f1 (ppm)

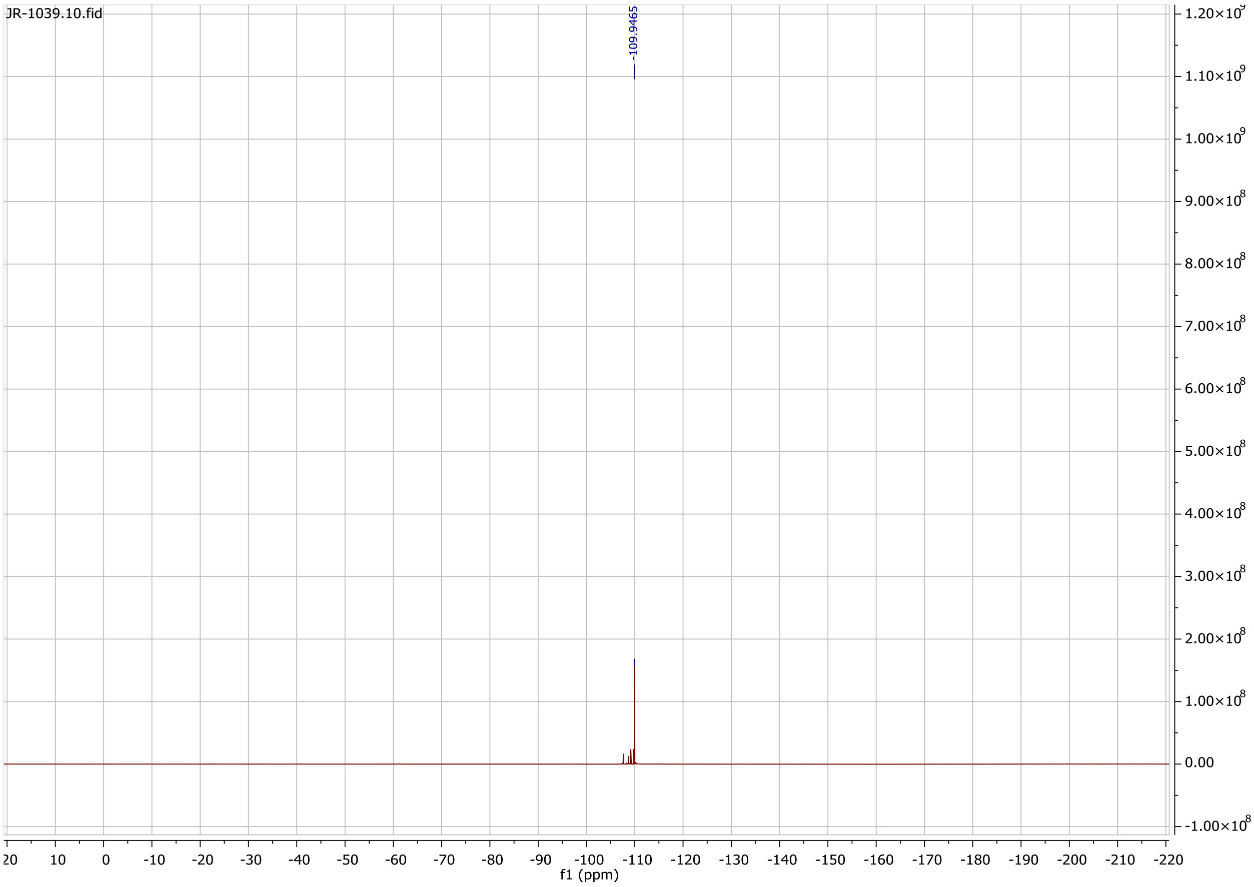
JR-1039.10.fid
-109.9465
1.20×10^9
1.10×10^9
1.00×10^9
9.00×10^8
8.00×10^8
7.00×10^8
6.00×10^8
5.00×10^8
4.00×10^8
3.00×10^8
2.00×10^8
1.00×10^8
0.00
-1.00×10^8
20 10 0 -10 -20 -30 -40 -50 -60 -70 -80 -90 -100 -110 -120 -130 -140 -150 -160 -170 -180 -190 -200 -210 -220
f1 (ppm)

3u

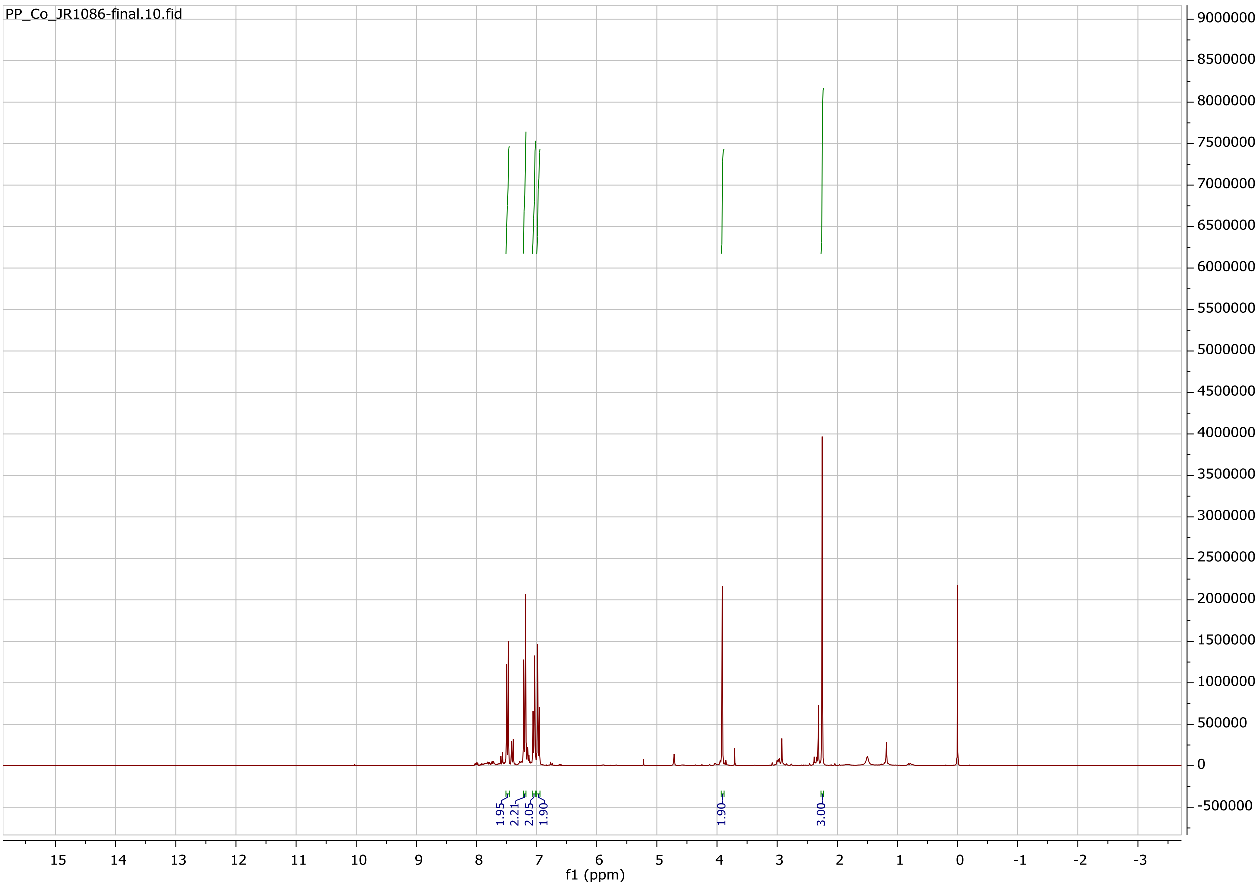
PP_Co_JR1086-final.10.fid
1.95
2.21
2.05
1.90
1.90
3.00
f1 (ppm)

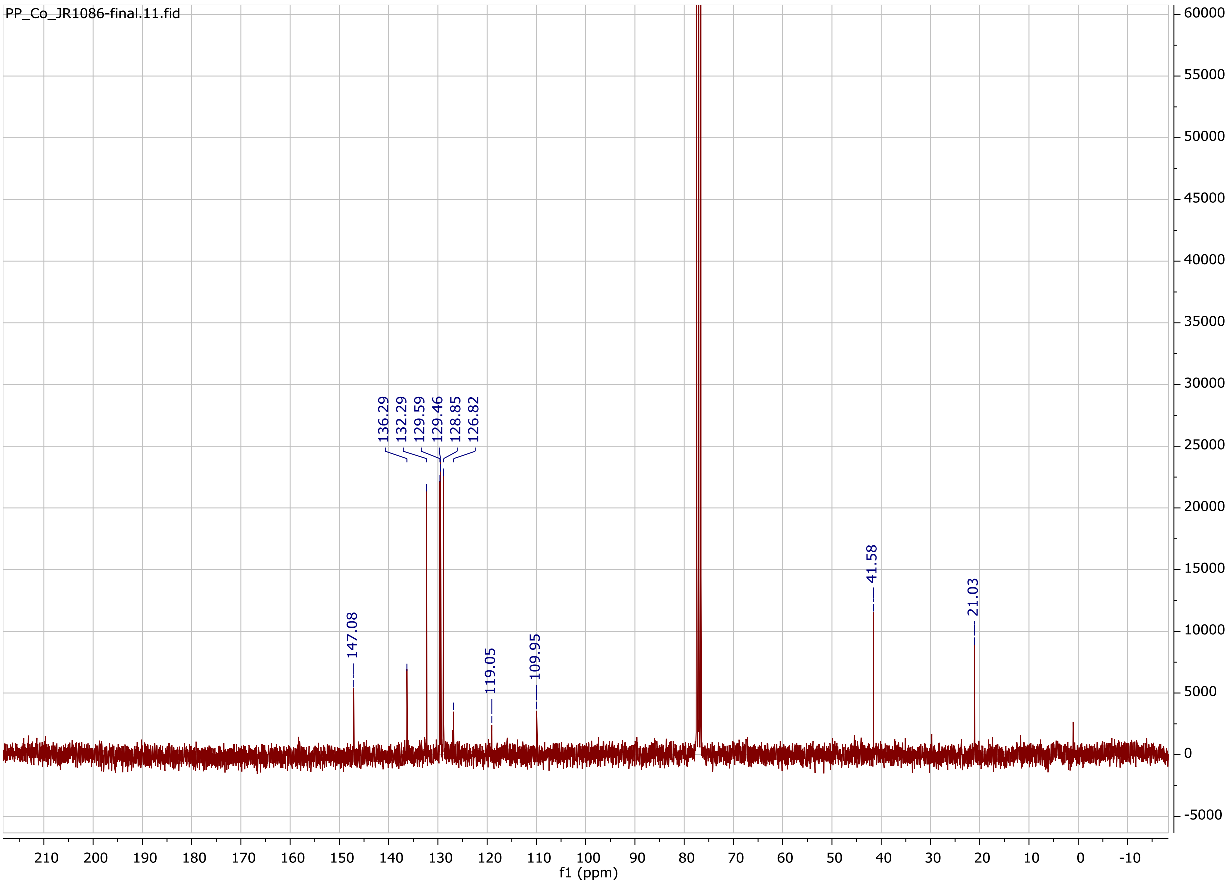
PP_Co_JR1086-final.11.fid
136.29
132.29
129.59
129.46
128.85
126.82
147.08
119.05
109.95
41.58
21.03
f1 (ppm)

3v

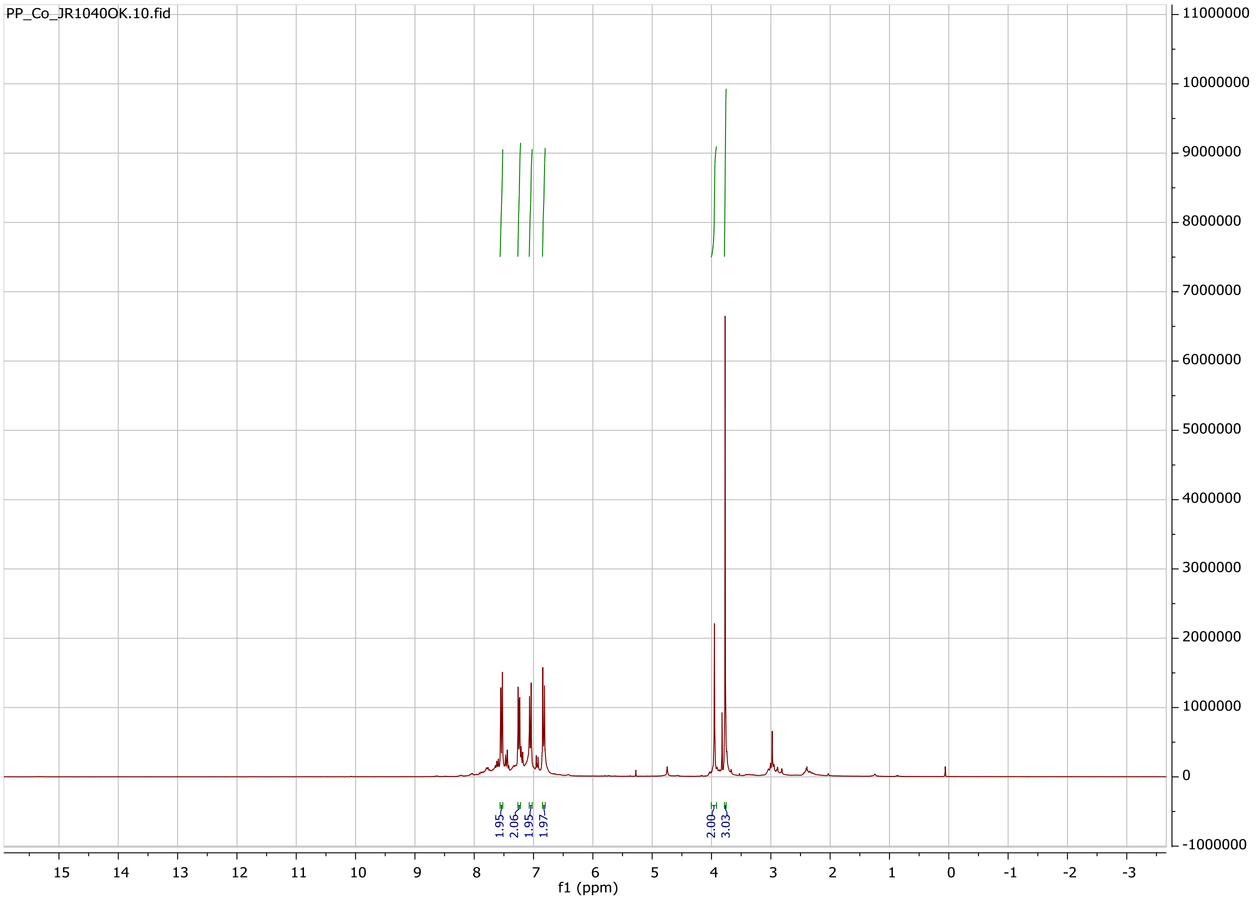
PP_Co_JR1040OK.10.fid
1.95
2.06
1.95
1.97
2.00
3.03
f1 (ppm)

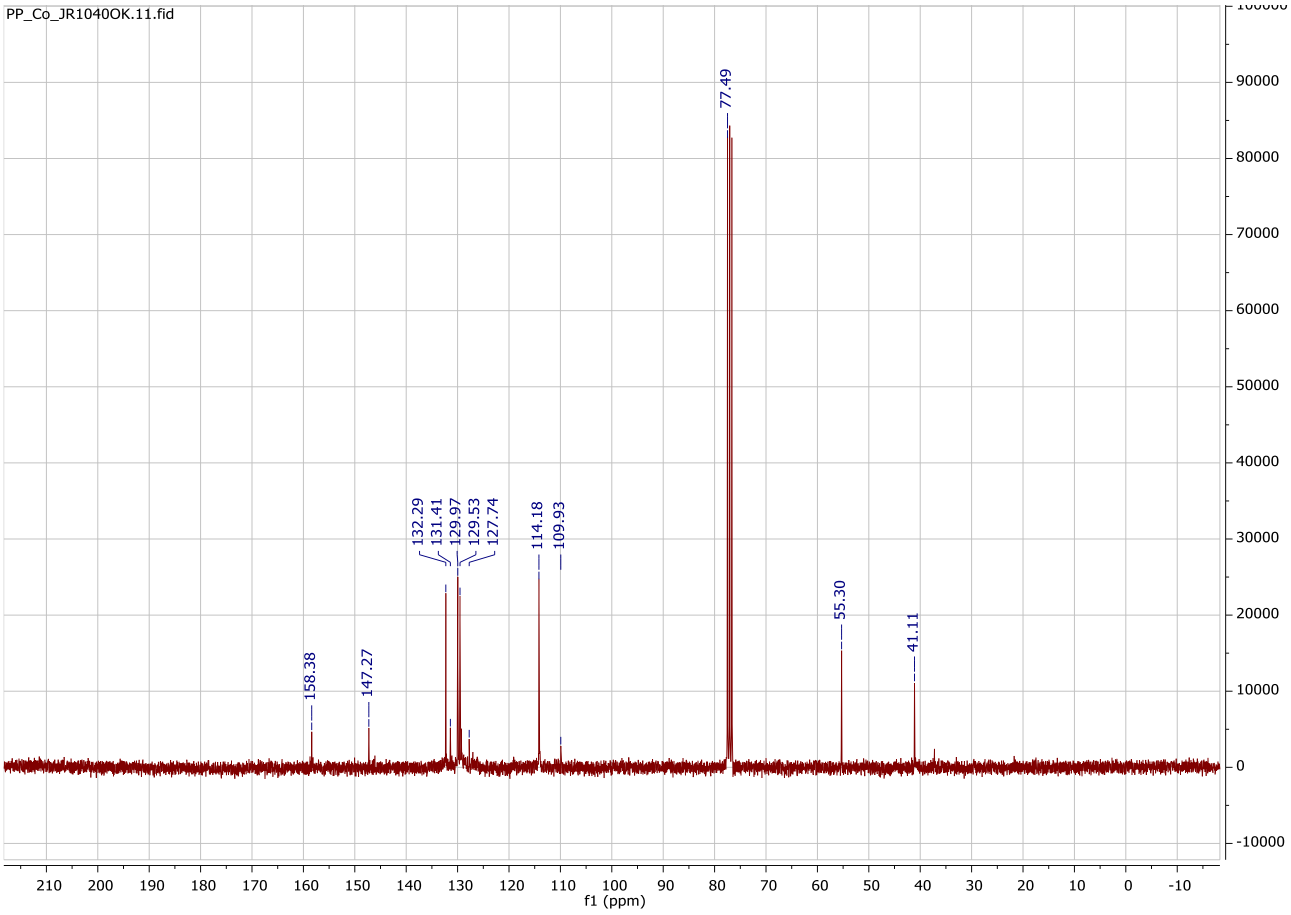
PP_Co_JR1040OK.11.fid
77.49
132.29
131.41
129.97
129.53
127.74
114.18
109.93
158.38
147.27
55.30
41.11
f1 (ppm)

3w

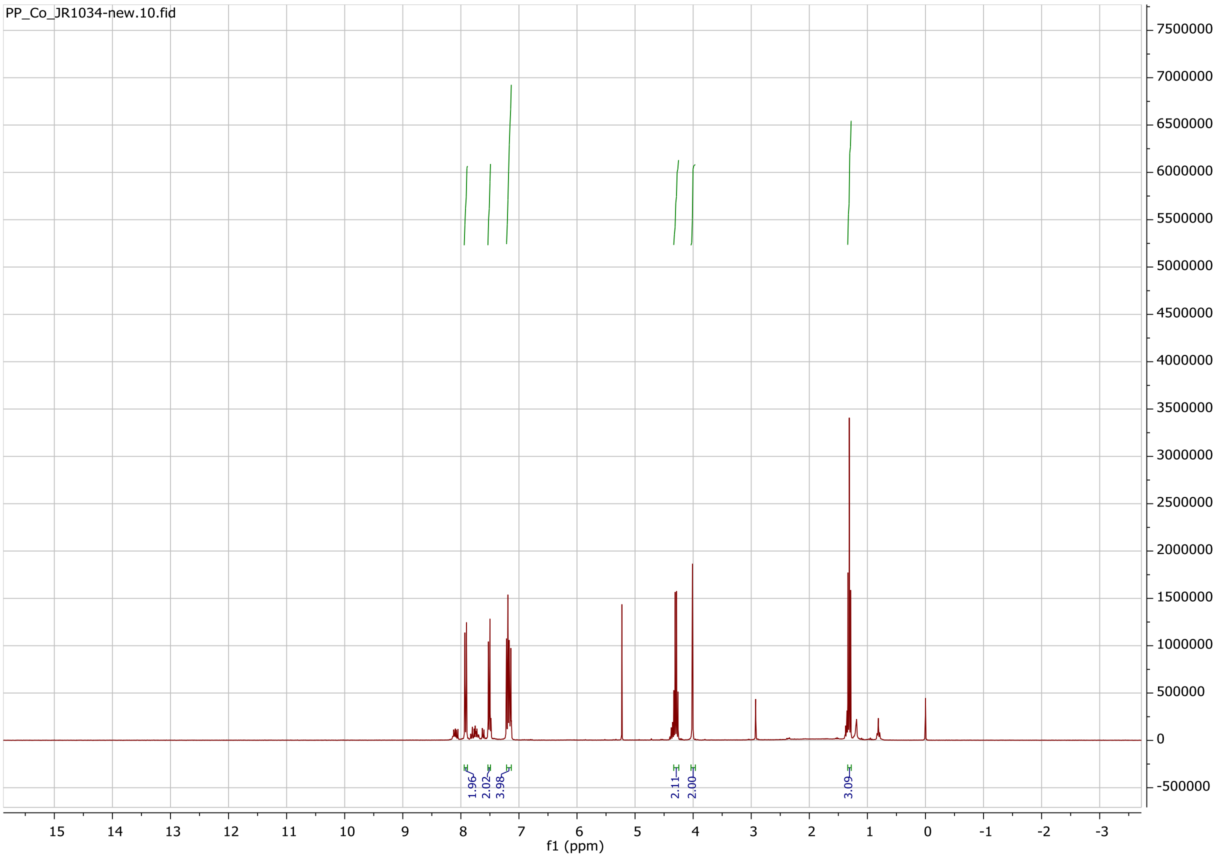
PP_Co_JR1034-new.10.fid
1.96
2.02
3.98
2.11
2.00
3.09
f1 (ppm)

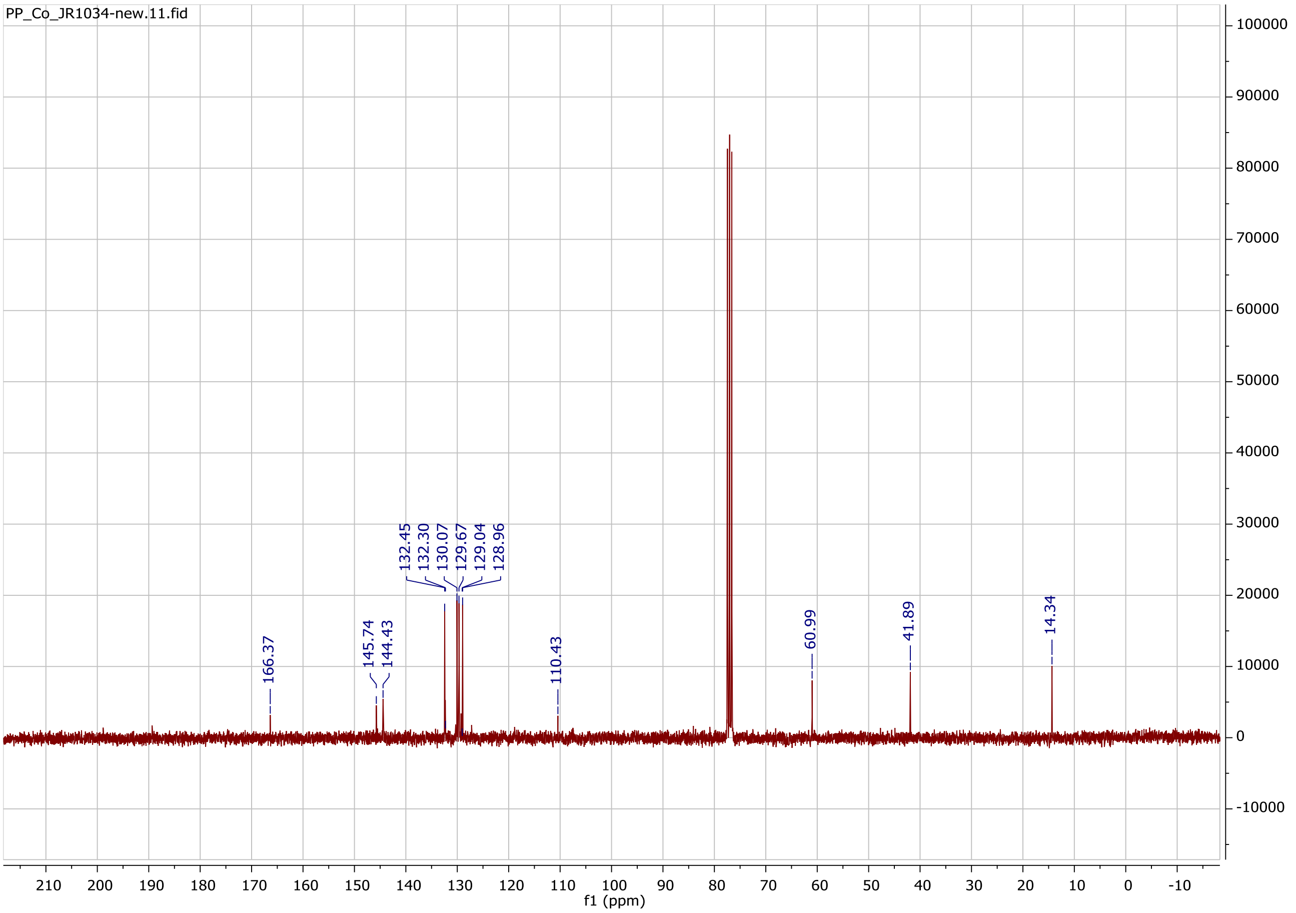
PP_Co_JR1034-new.11.fid
166.37
145.74
144.43
132.45
132.30
130.07
129.67
129.04
128.96
110.43
60.99
41.89
14.34
f1 (ppm)

3y

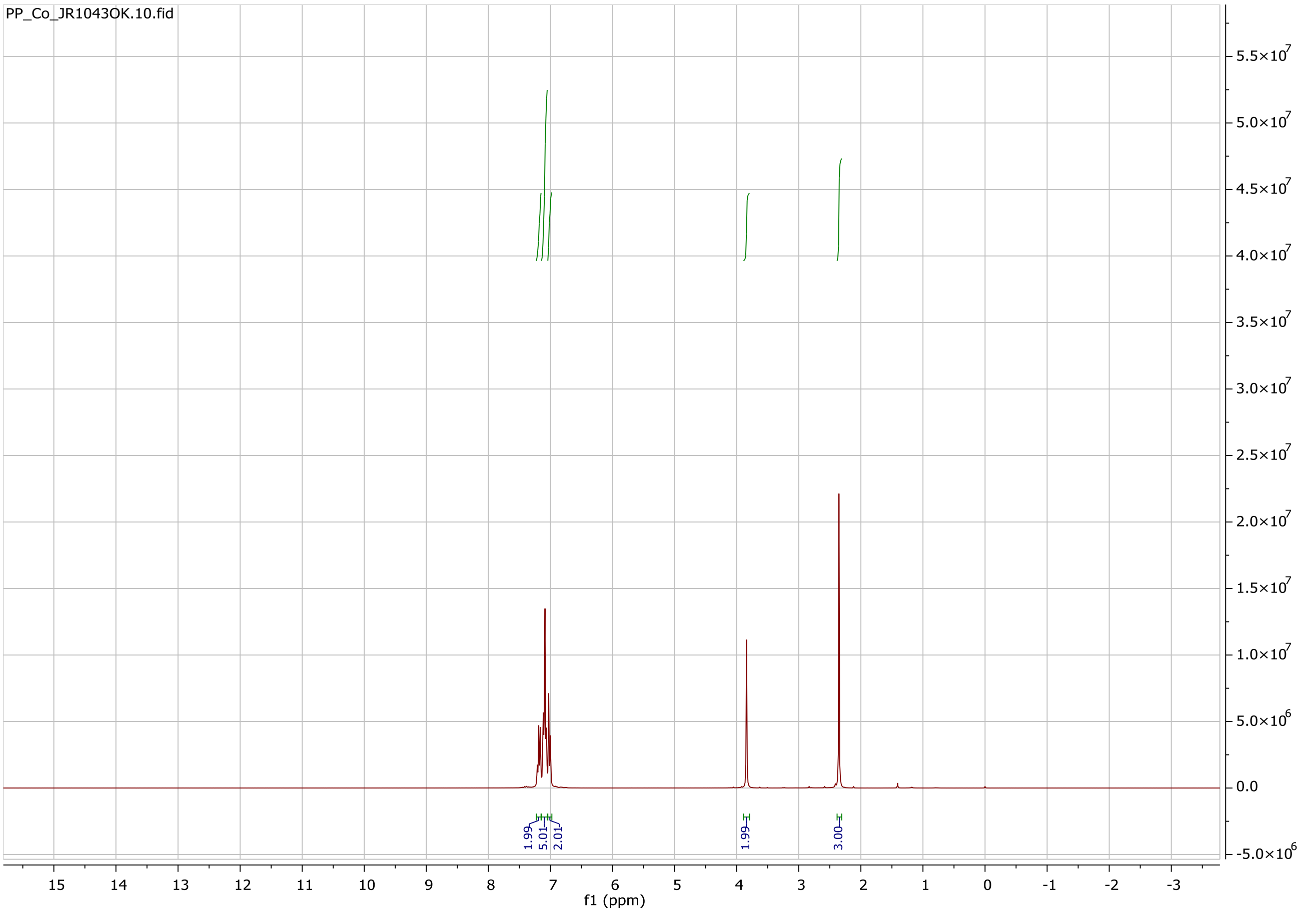
PP_Co_JR1043OK.10.fid
1.99
5.01
2.01
1.99
3.00
f1 (ppm)

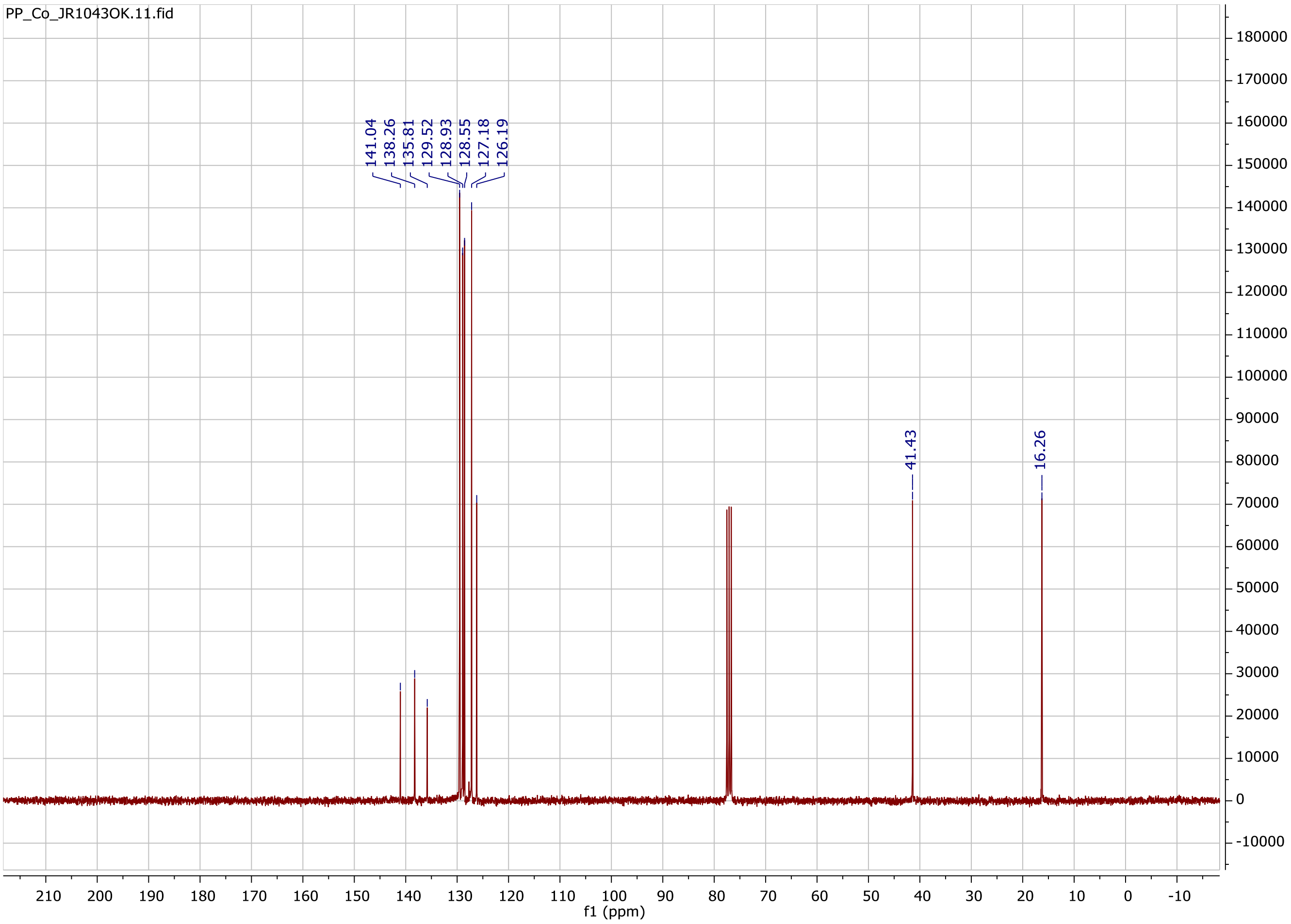
PP_Co_JR1043OK.11.fid
141.04
138.26
135.81
129.52
128.93
128.55
127.18
126.19
41.43
16.26
f1 (ppm)

3x

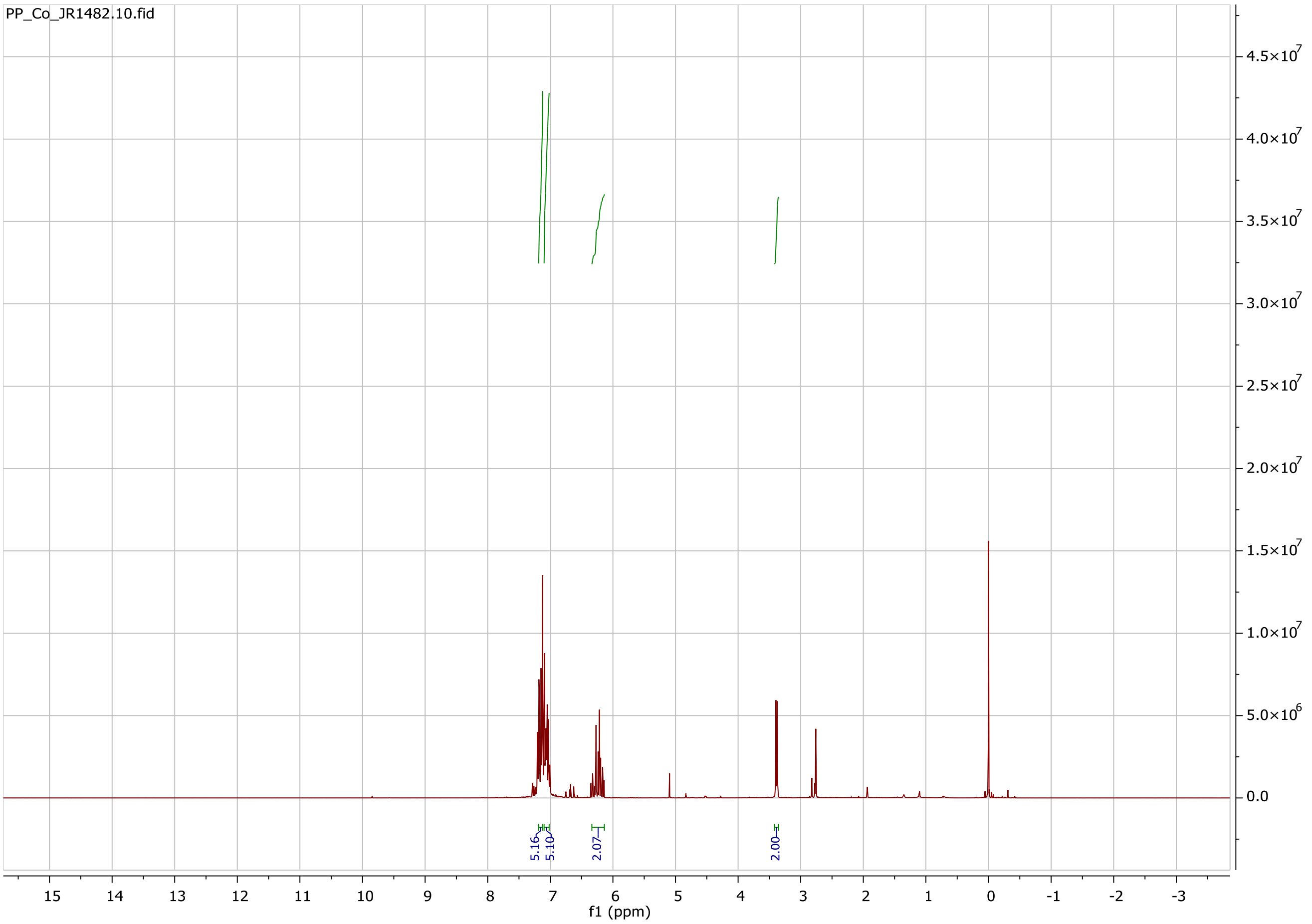
PP_Co_JR1482.10.fid
5.16
5.10
2.07
2.00
f1 (ppm)

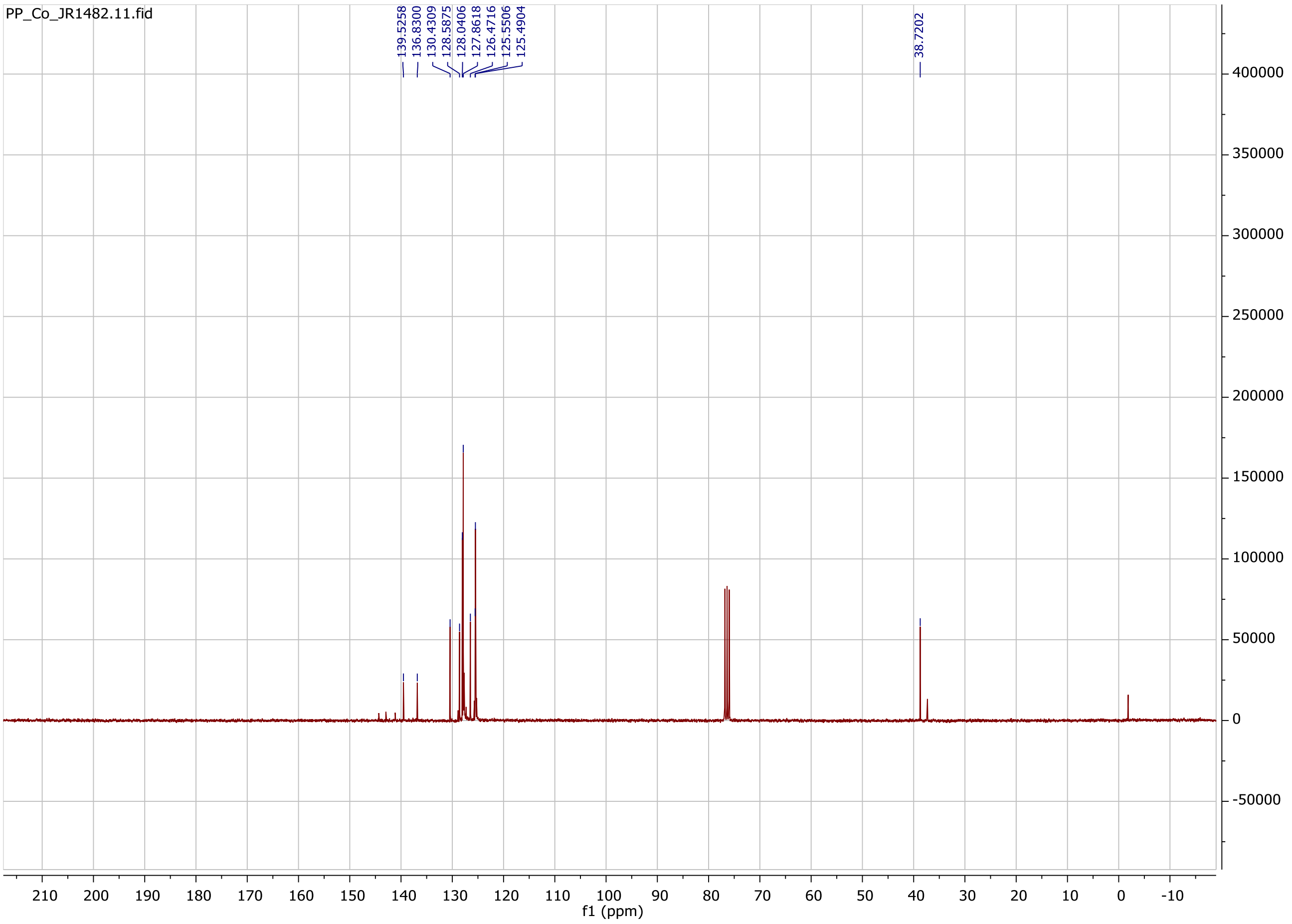
PP_Co_JR1482.11.fid
139.5258
136.8300
130.4309
128.5875
128.0406
127.8618
126.4716
125.5506
125.4904
38.7202
f1 (ppm)

3z

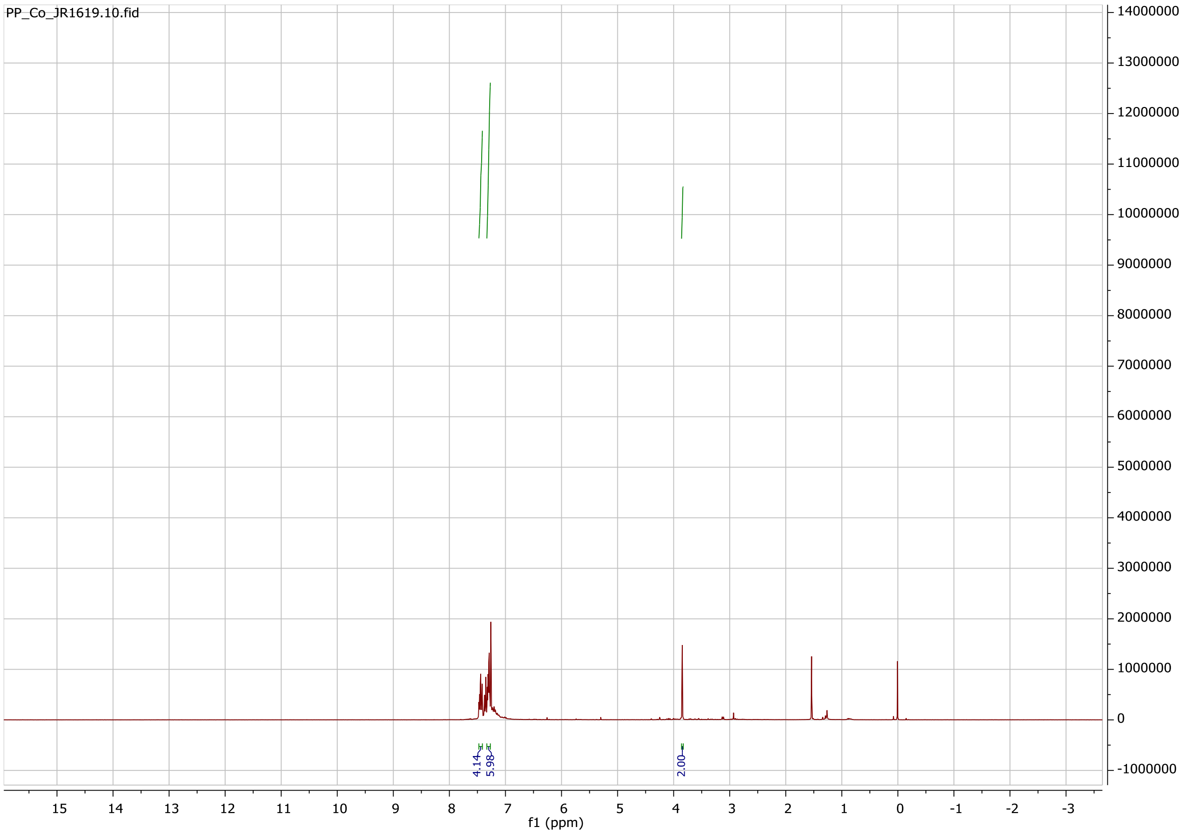
PP_Co_JR1619.10.fid
4.14
5.98
2.00
f1 (ppm)

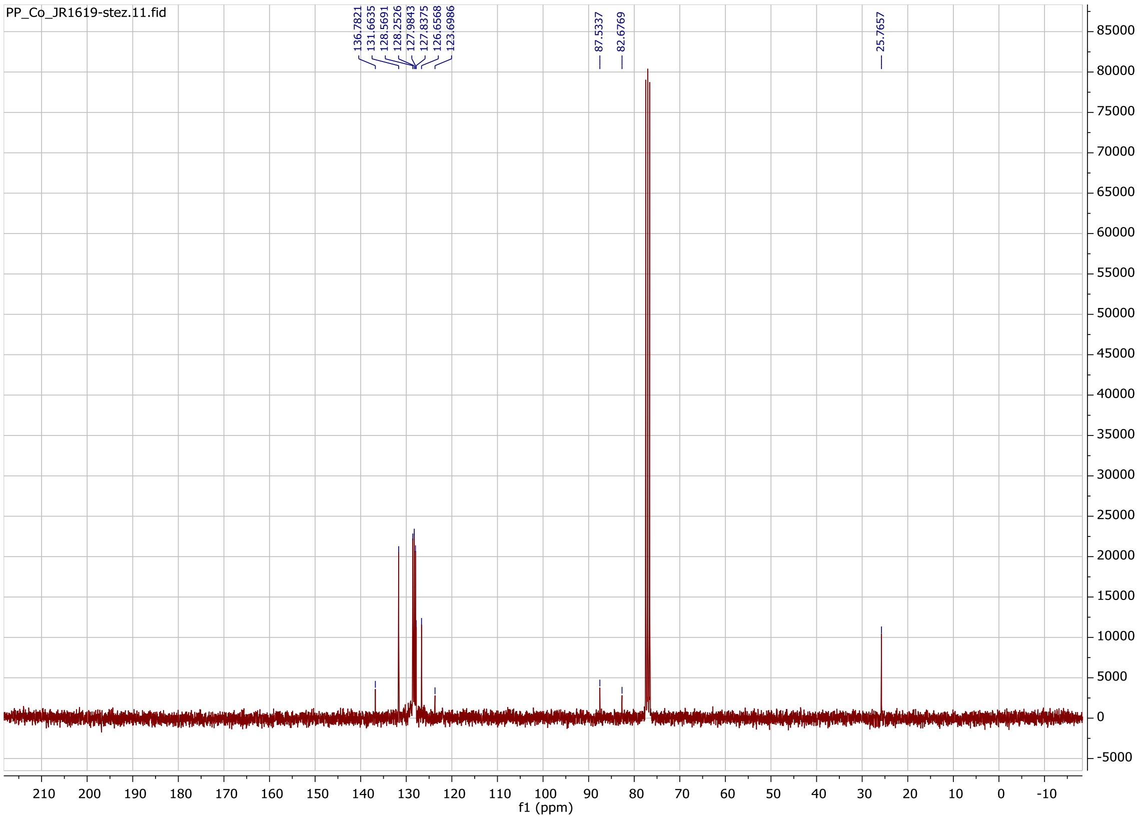
PP_Co_JR1619-stez.11.fid
136.7821
131.6635
128.5691
128.2526
127.9843
127.8375
126.6568
123.6986
87.5337
82.6769
25.7657
f1 (ppm)

3aa

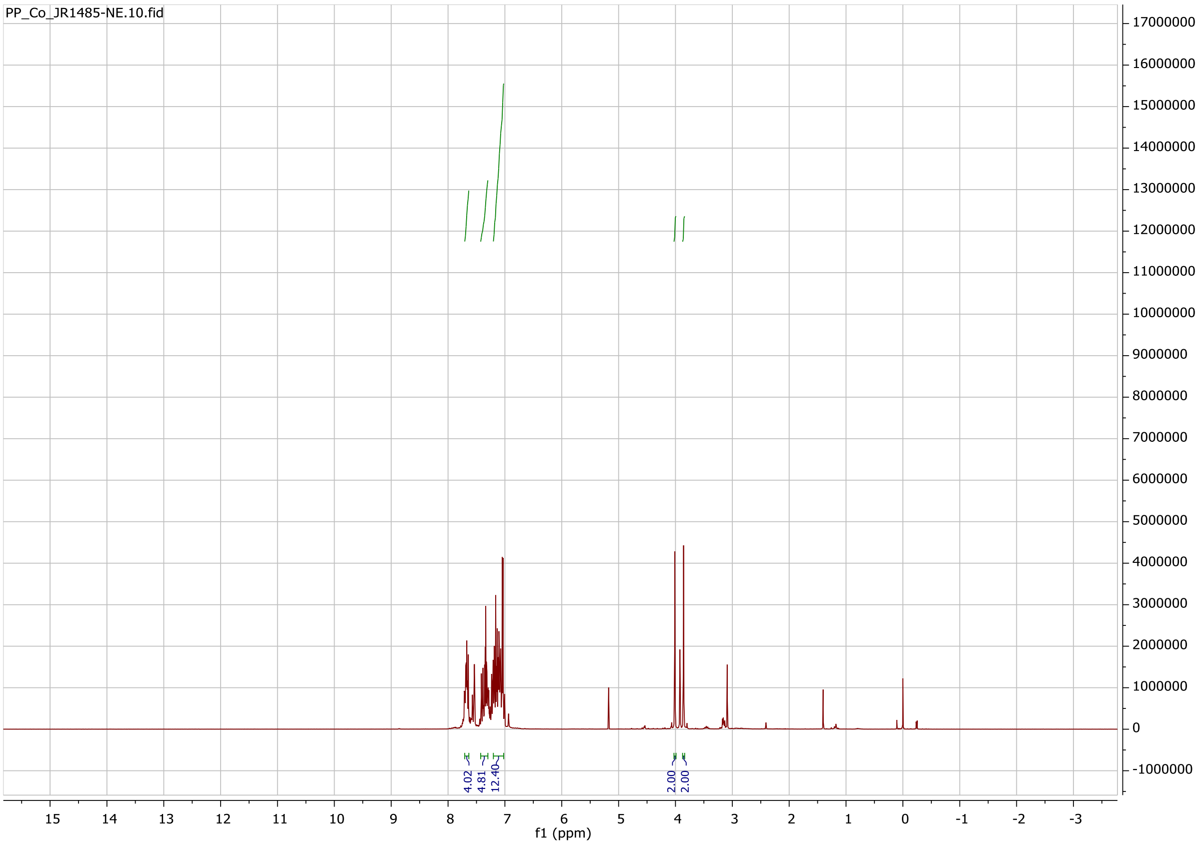

PP_Co_JR1485-NE.10.fid
4.02
4.81
12.40
2.00
2.00
f1 (ppm)

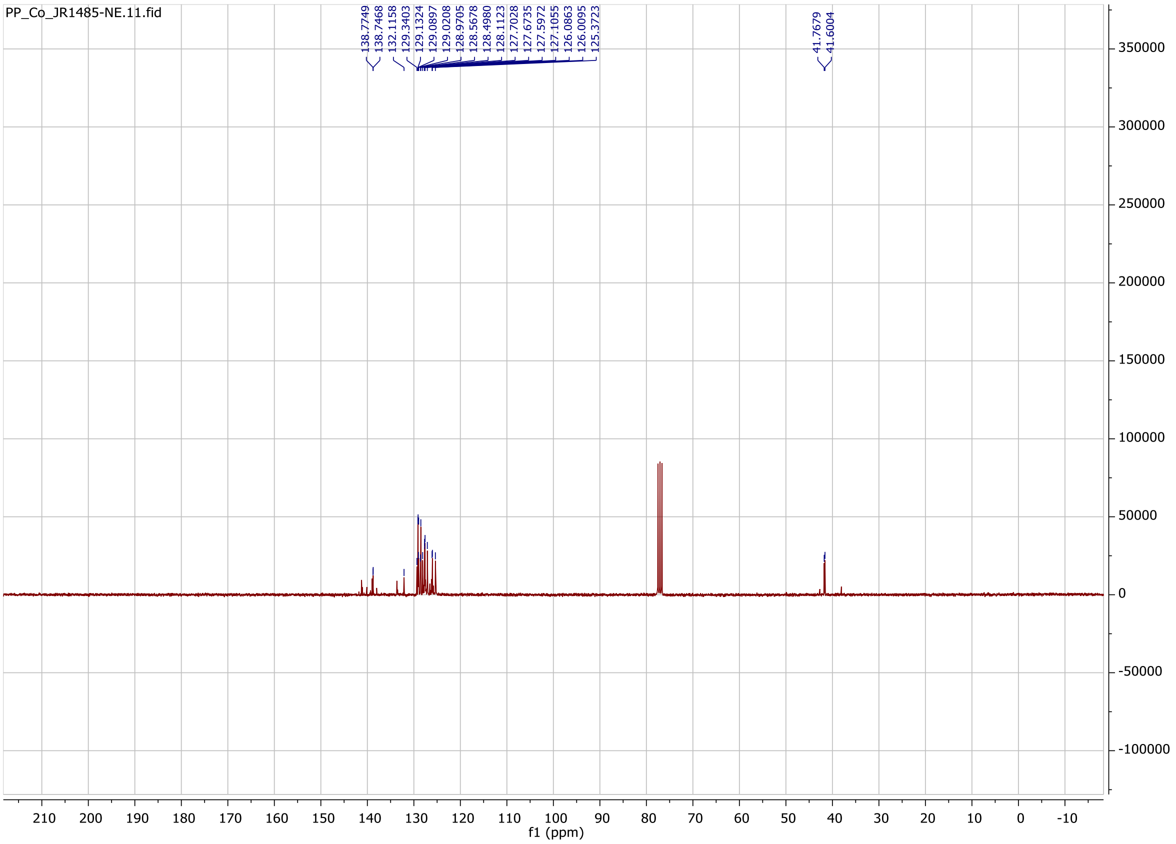

PP_Co_JR1485-NE.11.fid
138.7749
138.7468
132.1158
129.3403
129.1324
129.0897
129.0208
128.9705
128.5678
128.4980
128.1123
127.7028
127.6735
127.5972
127.1055
126.0863
126.0095
125.3723
41.7679
41.6004
f1 (ppm)

7. Computational methods

All calculations were conducted with Gaussian 16 package.[17] Structures of minima and transition states were optimized employing BP86 functional, def2-SVP basis set[18] Frequency analysis was performed at the same level of theory to provide correction to thermodynamic functions and confirm the nature of optimized structures (minima and transition states featured zero and one imaginary frequency, respectively). Single point energies were calculated with BP86 functional employing def2-TZVPP[18] basis set and solvation (N,N-dimethylacetamide) with the SMD model.[19] Molecular structures were visualized in CYLview.[20]

Optimized geometries, energies and corrections to thermodynamic functions.

DMA

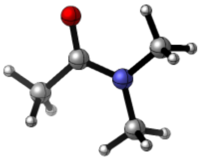

E (BP86/Def2-SVP) = -287.617611183
E (BP86/Def2-TZVPP/SMD(DMA)//BP86/Def2-SVP) = -287.954951993
E (M06/Def2-TZVPP/SMD(DMA)//BP86/Def2-SVP) = -287.756571282

Zero-point correction=	0.125690 (Hartree/Particle)
Thermal correction to Energy=	0.133471
Thermal correction to Enthalpy=	0.134415
Thermal correction to Gibbs Free Energy=	0.094124

Charge = 0 Multiplicity = 1

C	-0.73332600	-0.29532600	-0.00005500
N	0.60274300	0.08570900	-0.00023100
C	1.08670000	1.45390700	0.00000600
H	1.71666800	1.65771200	-0.89698700
H	0.25154800	2.17569200	-0.00113300
H	1.71476500	1.65826500	0.89823000
C	1.62513300	-0.95021700	0.00008500
H	2.27523300	-0.87013900	-0.90043000
H	2.27459800	-0.87029100	0.90108300
H	1.11434400	-1.93080600	-0.00014300
C	-1.78221700	0.81850800	-0.00005100
H	-1.70661400	1.46851100	0.89675600
H	-1.70682600	1.46829400	-0.89703500
H	-2.77212000	0.32829400	0.00011700
O	-1.06981700	-1.48084100	0.00015600

PhI

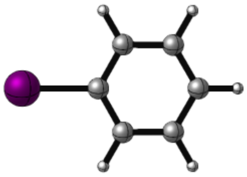

E (BP86/Def2-SVP) = -529.308006841
E (BP86/Def2-TZVPP/SMD(DMA)//BP86/Def2-SVP) = -529.574190307
E (M06/Def2-TZVPP/SMD(DMA)//BP86/Def2-SVP) = -529.313711921

Zero-point correction=	0.087443 (Hartree/Particle)
Thermal correction to Energy=	0.093464
Thermal correction to Enthalpy=	0.094408
Thermal correction to Gibbs Free Energy=	0.055582

Charge = 0 Multiplicity = 1

C	3.37028600	0.00001800	0.00000000
C	2.66436100	1.21554800	0.00000200
C	1.25754200	1.22499600	-0.00000200
C	0.56711800	-0.00003300	0.00000100
C	1.25757000	-1.22501400	0.00000200
C	2.66441600	-1.21551600	-0.00000200
H	4.47145500	0.00006100	0.00000200
H	3.20866700	2.17353900	-0.00000100
H	0.70740500	2.17768800	-0.00000200
H	0.70751400	-2.17775300	0.00000400
H	3.20870900	-2.17351400	-0.00000300
I	-1.56587800	0.00000000	0.00000000

I

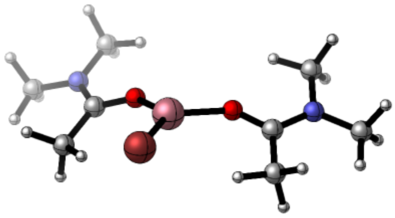

E (BP86/Def2-SVP) = -4532.24762354
E (BP86/Def2-TZVPP/SMD(DMA)//BP86/Def2-SVP) = -4533.44274602
E (M06/Def2-TZVPP/SMD(DMA)//BP86/Def2-SVP) = -4532.40012462

Zero-point correction=	0.253378 (Hartree/Particle)
Thermal correction to Energy=	0.275203
Thermal correction to Enthalpy=	0.276148
Thermal correction to Gibbs Free Energy=	0.195567

Charge = 0 Multiplicity = 3

Co	0.00014600	-0.54333900	0.32221900
O	1.44100400	0.74551700	0.09114900
C	2.63858700	0.64857400	-0.29783000
N	3.51546100	1.68382100	-0.05769400
C	3.11024600	-0.59218700	-1.02284500
C	4.90540000	1.69130100	-0.47866500
C	3.06849000	2.86019100	0.67523400
H	3.30386800	-0.39467400	-2.09969400
H	4.03378900	-1.02046700	-0.58152100
H	2.29650300	-1.34175300	-0.93678500
H	5.59410100	1.70383500	0.39788300
H	5.14178900	0.80390900	-1.09078500
H	5.12393000	2.59751200	-1.08817700
H	3.65197500	2.98598700	1.61504800

H	3.20090000	3.77970200	0.06198900
H	1.99933500	2.73565200	0.92254100
O	-1.43952200	0.74644600	0.08844100
C	-2.63779300	0.65004400	-0.29851100
N	-3.51409800	1.68518600	-0.05579400
C	-3.11110500	-0.59030900	-1.02313000
C	-4.90365700	1.69525900	-0.47787000
C	-3.06478000	2.86227300	0.67451400
H	-4.03317200	-1.01925400	-0.57931600
H	-3.30810700	-0.39212800	-2.09923400
H	-2.29701100	-1.33976900	-0.93982500
H	-5.11837400	2.59676400	-1.09584100
H	-5.14387000	0.80313100	-1.08152700
H	-5.59222300	1.71890200	0.39845400
H	-3.19155300	3.78048100	0.05801300
H	-3.65090700	2.99321000	1.61191000
H	-1.99685300	2.73448800	0.92543400
Br	-0.00205100	-2.85500000	0.27868100

II

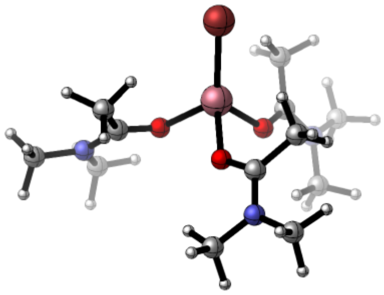

E (BP86/Def2-SVP) = -4819.88111172
E (BP86/Def2-TZVPP/SMD(DMA)//BP86/Def2-SVP) = -4821.39532232
E (M06/Def2-TZVPP/SMD(DMA)//BP86/Def2-SVP) = -4820.16546759

Zero-point correction=	0.378994 (Hartree/Particle)
Thermal correction to Energy=	0.410625
Thermal correction to Enthalpy=	0.411569
Thermal correction to Gibbs Free Energy=	0.305958

Charge = 0 Multiplicity = 3

Co	-0.02521200	0.13853300	0.63021800
O	-1.36216200	1.12154400	-0.51491800
C	-2.61634700	1.07187300	-0.43427700
N	-3.38873300	1.79843200	-1.32324500
C	-3.27933700	0.23288400	0.63590900
C	-4.83900900	1.77302600	-1.36926300
C	-2.74093200	2.66253000	-2.29847600
H	-4.20728500	0.67962200	1.04488200
H	-3.51505500	-0.78583400	0.26092900
H	-2.53555000	0.12050300	1.45585300
H	-5.18794500	1.57616400	-2.40874500
H	-5.24542900	0.97653600	-0.72168700
H	-5.28269700	2.74518200	-1.04842300

H	-3.00635700	2.35860700	-3.33677100
H	-3.05456900	3.72243500	-2.16114300
H	-1.64728700	2.58277500	-2.16355200
O	1.52914100	0.57757300	-0.57288300
C	2.43868200	1.44044700	-0.45830000
N	3.36979500	1.58813600	-1.47129100
C	2.52158100	2.31172300	0.77529500
C	4.43651800	2.57206100	-1.47055400
C	3.31254900	0.71775400	-2.63573000
H	3.55574000	2.44196900	1.15360400
H	2.09198100	3.32112800	0.59351600
H	1.90498500	1.81537900	1.55674200
H	4.41651900	3.16699600	-2.41231900
H	4.33100500	3.27415600	-0.62524900
H	5.44138000	2.09206300	-1.40232300
H	3.19934400	1.31366800	-3.56979700
H	4.24297800	0.11181700	-2.72795300
H	2.44423200	0.04347100	-2.52500700
Br	0.00072100	0.27815400	2.99758200
O	-0.37786500	-1.59197300	-0.33967900
C	0.26443100	-2.66985800	-0.23090000
N	-0.05610100	-3.74453900	-1.04298300
C	1.38224800	-2.78678200	0.78058800
C	0.62193700	-5.02747700	-1.01745200
C	-1.18185500	-3.64111800	-1.95849300
H	1.47509700	-3.79184400	1.23784800
H	2.36095800	-2.51531800	0.32964300
H	1.16637900	-2.03940500	1.57705200
H	0.85067700	-5.35809700	-2.05569800
H	1.57748100	-4.96356100	-0.46757900
H	-0.00102900	-5.82404900	-0.54466400
H	-0.86563400	-3.87979500	-2.99900200
H	-1.99714200	-4.34760900	-1.67815600
H	-1.56772200	-2.60647900	-1.92086100

III

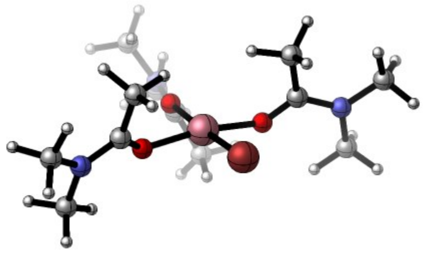

E (BP86/Def2-SVP) = -4819.85388554
E (BP86/Def2-TZVPP/SMD(DMA)//BP86/Def2-SVP) = -4821.37283608
E (M06/Def2-TZVPP/SMD(DMA)//BP86/Def2-SVP) = -4820.16546759

Zero-point correction=	0.380846 (Hartree/Particle)
Thermal correction to Energy=	0.411288
Thermal correction to Enthalpy=	0.412232
Thermal correction to Gibbs Free Energy=	0.313760

Charge = 0 Multiplicity = 1

Co	-0.13231300	-0.09721700	-0.09400300
O	1.73381900	-0.38256300	-0.26109600
C	2.45579400	-1.14353600	0.44735500
N	3.66505600	-1.56071800	-0.05635400
C	2.01765500	-1.58503600	1.82284100
C	4.61507500	-2.37632500	0.67873800
C	4.06956700	-1.16142100	-1.39682100
H	1.71574000	-2.65350200	1.82044700
H	2.79758900	-1.41935800	2.59687600
H	1.10666900	-0.99373500	2.04931900
H	5.53885900	-1.80539700	0.93431200
H	4.17123100	-2.75639100	1.61508400
H	4.92187200	-3.25294900	0.06552600
H	4.89105400	-0.40828800	-1.36973300
H	4.43257600	-2.04647100	-1.96307800
H	3.19705500	-0.72553200	-1.91515900
O	0.13126100	1.74395900	0.29750800
C	0.87696700	2.59277700	-0.26860500
N	1.14296700	3.78688500	0.37758500
C	1.47119100	2.32860400	-1.63192400
C	1.90694600	4.86735300	-0.21733800
C	0.46883200	4.09254000	1.63080100
H	1.31002600	3.17311700	-2.33503500
H	2.56110100	2.11423200	-1.58996100
H	0.95987000	1.41773000	-2.00530000
H	2.54400600	5.34730700	0.55783800
H	2.57362800	4.49283900	-1.01509100
H	1.25515800	5.66413100	-0.65228200
H	1.19979500	4.45769400	2.38550900
H	-0.30825200	4.88113200	1.49485900
H	-0.02035400	3.17343200	2.00016200
Br	-0.53247600	-2.42951600	-0.36862600
O	-1.97594100	0.28836000	-0.28134100
C	-2.98483300	-0.15142000	0.33266000
N	-4.23159200	0.05374200	-0.21289700
C	-2.85659900	-0.86389800	1.65899100
C	-5.47005900	-0.40252800	0.39070700
C	-4.36540300	0.75990300	-1.47950800
H	-3.50607100	-0.41651300	2.44166800
H	-3.08507100	-1.94560800	1.56426400
H	-1.78852500	-0.78102400	1.94187000
H	-6.05066800	-1.01574900	-0.33521000
H	-5.27396700	-1.02616400	1.27990300
H	-6.11572600	0.45365900	0.69742300
H	-4.87624000	0.11884400	-2.23218000
H	-4.96460100	1.69011900	-1.35327600
H	-3.35656600	1.02090400	-1.84505000

IV

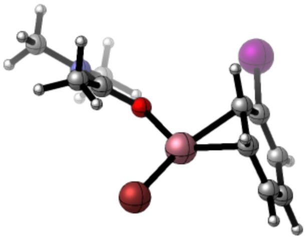

E (BP86/Def2-SVP) = -4773.94534524
E (BP86/Def2-TZVPP/SMD(DMA)//BP86/Def2-SVP) = -4775.06345145
E (M06/Def2-TZVPP/SMD(DMA)//BP86/Def2-SVP) = -4773.96273581

Zero-point correction=	0.215049 (Hartree/Particle)
Thermal correction to Energy=	0.234862
Thermal correction to Enthalpy=	0.235807
Thermal correction to Gibbs Free Energy=	0.159067

Charge = 0 Multiplicity = 3

Co	-1.16116400	-0.66569600	0.01891200
C	-0.47737900	-3.28842700	-0.15548400
C	-0.51569200	-2.34055000	0.92566200
C	0.52585000	-1.32893700	1.00793700
C	1.56454300	-1.36208400	0.01620800
C	1.56466300	-2.26300000	-1.04262500
H	-1.15338500	-2.54576200	1.80166400
H	0.68346700	-0.76271900	1.93943400
H	2.36627400	-2.24935500	-1.79525200
C	0.51925900	-3.23445100	-1.12298100
H	0.52114100	-3.95329900	-1.95742300
O	-0.54273700	1.21104900	-0.17850700
C	-1.01612900	2.33474200	0.14166700
N	-0.46148300	3.46587800	-0.38139900
C	-2.17539800	2.43774100	1.10919700
C	-0.89636900	4.81877500	-0.06069100
C	0.63941900	3.36954200	-1.33834000
H	-1.82823600	2.78532800	2.10559900
H	-2.96589300	3.12869700	0.75623400
H	-2.61542500	1.42525500	1.20684200
H	-1.31594000	5.32358600	-0.95941000
H	-1.66424700	4.81776400	0.73103900
H	-0.03281000	5.42212100	0.29509700
H	0.33875100	3.80207100	-2.31745300
H	1.52256000	3.92981200	-0.96363800
H	0.91136800	2.30797000	-1.46888700
H	-1.25974600	-4.06116400	-0.20917200
I	3.18996500	0.01331300	0.21464500
Br	-3.48711300	-0.88319300	-0.10962700

V

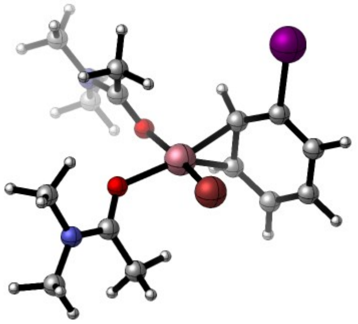

E (BP86/Def2-SVP) = -5061.55089043
E (BP86/Def2-TZVPP/SMD(DMA)//BP86/Def2-SVP) = -5062.99677873
E (M06/Def2-TZVPP/SMD(DMA)//BP86/Def2-SVP) = -5061.68928383

Zero-point correction=	0.342558 (Hartree/Particle)
Thermal correction to Energy=	0.371075
Thermal correction to Enthalpy=	0.372020
Thermal correction to Gibbs Free Energy=	0.277309

Charge = 0 Multiplicity = 1

Co	0.52823500	-0.06473300	0.51040000
C	-2.49683600	-0.47166800	0.61980400
C	-1.38522300	0.34761200	1.03243900
C	-0.57990400	-0.13719400	2.15942000
C	-0.93858000	-1.39506200	2.77544900
C	-1.98908900	-2.15037000	2.29424500
H	-1.49361500	1.43476000	0.86748700
H	-0.08164700	0.60466500	2.81273000
H	-2.24212200	-3.11592800	2.76170600
C	-2.78779300	-1.69297900	1.18938100
H	-3.62516100	-2.30652000	0.82681100
O	2.39202700	-0.14546200	-0.14520300
C	3.20799400	-1.10082900	-0.02332300
N	4.12498500	-1.32264400	-1.01037600
C	3.23542000	-1.95584500	1.22582800
C	5.19785500	-2.30262600	-0.92947200
C	4.03901900	-0.57236100	-2.25834400
H	3.17667800	-3.03632300	0.99192000
H	4.15425100	-1.76294500	1.82260700
H	2.34109400	-1.68692400	1.81723100
H	6.18313300	-1.81656800	-1.10610500
H	5.22513400	-2.78236200	0.06346800
H	5.06604900	-3.09648100	-1.69839900
H	4.81854400	0.22086400	-2.31279000
H	4.18151900	-1.26222800	-3.11638100
H	3.04033100	-0.10609700	-2.32462500
O	0.98035300	1.79534300	0.97000300
C	1.08195000	2.68309200	0.08001600
N	1.58235200	3.91360300	0.40984500
C	0.66041000	2.39349900	-1.34473000
C	1.72804000	5.01387400	-0.53084900
C	1.95014800	4.20769600	1.79120000

H	-0.02610200	3.16365300	-1.75055400
H	1.53788300	2.31128300	-2.01920400
H	0.13512800	1.41321100	-1.33008900
H	2.75817100	5.42947400	-0.47440400
H	1.55044000	4.68080000	-1.56779000
H	1.01733300	5.84005000	-0.30017700
H	3.00805100	4.54532900	1.84544200
H	1.30580600	5.01437700	2.20566400
H	1.82316000	3.29342100	2.39658100
Br	0.22345900	-2.17938500	-0.49299900
H	-0.37922200	-1.73442200	3.66355400
I	-3.73639900	0.26876200	-0.97135100

VI

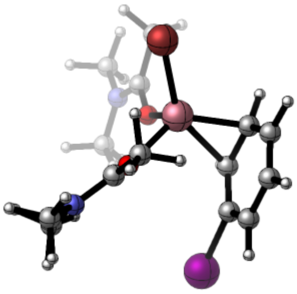

E (BP86/Def2-SVP) = -5061.55131687
E (BP86/Def2-TZVPP/SMD(DMA)//BP86/Def2-SVP) = -5063.02241666
E (M06/Def2-TZVPP/SMD(DMA)//BP86/Def2-SVP) = -5061.73254078

Zero-point correction=	0.342487 (Hartree/Particle)
Thermal correction to Energy=	0.371044
Thermal correction to Enthalpy=	0.371988
Thermal correction to Gibbs Free Energy=	0.277500

Charge = 0 Multiplicity = 3

Co	0.93548600	0.55006700	0.53028900
C	-0.34306300	1.56556500	2.89083600
C	0.28096300	0.35359600	2.42700900
C	-0.50797300	-0.58153800	1.64274400
C	-1.87549000	-0.24993500	1.39274500
C	-2.45815200	0.93783300	1.81556100
H	1.19212200	0.00060700	2.94484400
H	-0.17819000	-1.62301200	1.51612300
H	-3.51230200	1.16316100	1.59811100
C	-1.66474000	1.85519100	2.57586200
H	-2.12571600	2.79109600	2.93148900
O	-0.23451700	0.84239400	-1.11873400
C	-0.69263100	1.91484500	-1.58806900
N	-1.27124100	1.90504800	-2.83132400
C	-0.58561000	3.18618100	-0.78200200
C	-1.76506600	3.07025500	-3.55002300
C	-1.39649400	0.64045200	-3.55140900
H	0.49284200	3.44326000	-0.68800400
H	-1.15454000	4.05235900	-1.16180700

H	-0.92811000	2.95005800	0.24642300
H	-2.86650700	3.00956200	-3.70246400
H	-1.53626100	4.00580700	-3.01342800
H	-1.28836200	3.12908800	-4.55360200
H	-2.44898900	0.49071300	-3.87542100
H	-0.74821100	0.63537500	-4.45597800
H	-1.09437900	-0.18217800	-2.88046500
O	1.60959200	-1.27743100	-0.22457100
C	2.70152400	-1.89165000	-0.25409900
N	2.78166400	-3.09147200	-0.91782000
C	3.93689800	-1.34826100	0.43656300
C	3.98017100	-3.91209200	-0.99776500
C	1.60577700	-3.62761300	-1.59569200
H	4.21700800	-1.97410400	1.31041400
H	4.81004300	-1.30070900	-0.24498800
H	3.70822700	-0.31784700	0.77625800
H	4.30731800	-4.03492500	-2.05503800
H	4.81173400	-3.46459100	-0.42764700
H	3.78510500	-4.92775600	-0.58611000
H	1.80962800	-3.76106400	-2.68102800
H	1.32781200	-4.61740500	-1.17107100
H	0.76645900	-2.92290200	-1.46075200
H	0.24503300	2.26309500	3.50763600
I	-3.07651200	-1.67457900	0.33276700
Br	2.72385800	2.15432800	0.51397000

TS1

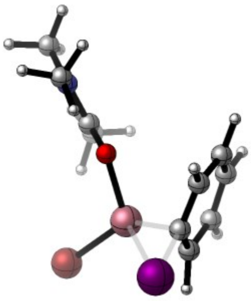

E (BP86/Def2-SVP) = -4773.94086764
E (BP86/Def2-TZVPP/SMD(DMA)//BP86/Def2-SVP) = -4775.05735643
E (M06/Def2-TZVPP/SMD(DMA)//BP86/Def2-SVP) = -4773.95226262

Zero-point correction= 0.214381 (Hartree/Particle)
Thermal correction to Energy= 0.233623
Thermal correction to Enthalpy= 0.234567
Thermal correction to Gibbs Free Energy= 0.159381

Charge = 0 Multiplicity = 3

Co	-0.17623300	0.66941400	0.40409900
C	-0.19320300	-1.22405100	2.89688300
C	0.41544000	0.02915000	2.73973500
C	1.30066700	0.29484800	1.63527500

C	1.45147800	-0.76107700	0.65888200
C	0.90575100	-2.05675500	0.88915200
H	0.26017100	0.81659900	3.49447000
H	1.98713100	1.15747400	1.66112700
H	1.11406700	-2.86746400	0.17465400
C	0.08748700	-2.28624600	1.99866000
H	-0.35306500	-3.28237400	2.15790000
O	-1.55116300	-0.64153000	-0.28574800
C	-2.80484300	-0.54285300	-0.19716400
N	-3.59283900	-1.34500000	-0.97295300
C	-3.39888800	0.46610700	0.75666400
C	-5.04839100	-1.40174400	-0.92924300
C	-2.96977000	-2.24252300	-1.94515600
H	-2.86031600	1.42410900	0.57864100
H	-4.48510100	0.63895800	0.66493400
H	-3.16558300	0.15415000	1.79619300
H	-5.37927000	-2.45860200	-0.83358400
H	-5.45126100	-0.84237100	-0.06856500
H	-5.49430100	-0.98901100	-1.86178600
H	-3.15811700	-3.30439100	-1.67373100
H	-3.39400400	-2.05959200	-2.95562400
H	-1.88209800	-2.05720400	-1.95959100
H	-0.86293800	-1.39483600	3.75524600
I	2.96129700	-0.55736400	-0.86422800
Br	-0.57088400	2.84082200	-0.34178200

TS2

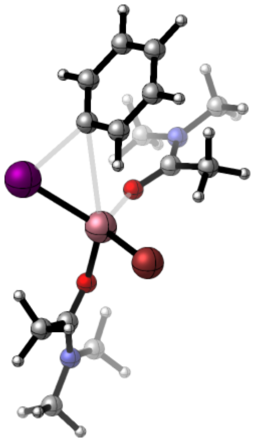

E (BP86/Def2-SVP) = -5061.55978387
E (BP86/Def2-TZVPP/SMD(DMA)//BP86/Def2-SVP) = -5063.00653604
E (M06/Def2-TZVPP/SMD(DMA)//BP86/Def2-SVP) = -5061.71206288

Zero-point correction= 0.341163 (Hartree/Particle)
Thermal correction to Energy= 0.370575
Thermal correction to Enthalpy= 0.371519
Thermal correction to Gibbs Free Energy= 0.271460

Charge = 0 Multiplicity = 3

Co	0.55532000	-0.02556000	0.14857300
C	-4.75244800	-0.77229000	0.83416900
C	-3.70697200	-1.32379700	1.59865300
C	-2.44886300	-1.57661700	1.01128400
C	-2.26929900	-1.26919100	-0.34148100
C	-3.29509300	-0.72818800	-1.12562700
H	-3.86498300	-1.55888700	2.66477900
H	-1.61929000	-1.97599200	1.62037300
H	-3.13499700	-0.51280900	-2.19597600
C	-4.54598100	-0.47228800	-0.52632000
H	-5.36616300	-0.04690700	-1.12985400
O	-0.44741200	1.62905600	-0.35537400
C	-1.37191700	2.29401100	0.18005700
N	-2.02570100	3.24525200	-0.55907500
C	-1.77016000	2.05957100	1.61934400
C	-3.15050200	4.02970100	-0.07415600
C	-1.65573300	3.46971700	-1.95209200
H	-1.81027400	2.99961800	2.20604400
H	-2.76344600	1.56533700	1.67595000
H	-1.02178600	1.37672900	2.06988500
H	-4.05702100	3.83255800	-0.68933300
H	-3.38849400	3.78142900	0.97376400
H	-2.92860200	5.11876900	-0.13670600
H	-1.38353600	4.53581400	-2.11375300
H	-0.79462400	2.82486100	-2.19978400
H	-2.50447300	3.22162200	-2.62718200
O	2.36115100	0.73288100	-0.36896200
C	3.49977100	0.20981300	-0.24473300
N	4.61475900	0.99290400	-0.39461300
C	3.60638200	-1.26160700	0.06607400
C	5.99109100	0.54898700	-0.22827600
C	4.45930800	2.40716900	-0.72439900
H	2.87557600	-1.78404500	-0.58867600
H	4.60377400	-1.71596300	-0.06515500
H	3.24425300	-1.42021800	1.10623200
H	6.51517900	1.20489500	0.50124400
H	6.04050100	-0.48476800	0.15196800
H	6.54901600	0.59990500	-1.19072400
H	4.87647800	3.04432800	0.08621800
H	4.99954400	2.64536200	-1.66684800
H	3.38456200	2.62755400	-0.84598100
I	-0.22882900	-1.92415600	-1.45703900
Br	0.84134300	-0.59649600	2.46353000
H	-5.73472000	-0.58396800	1.29707600

TS3

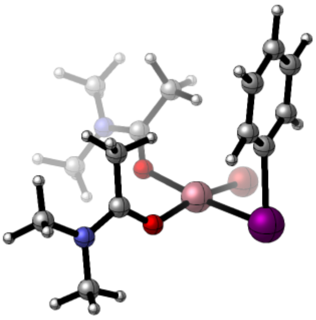

E (BP86/Def2-SVP) = -5061.52496459
E (BP86/Def2-TZVPP/SMD(DMA)//BP86/Def2-SVP) = -5062.98295404
E (M06/Def2-TZVPP/SMD(DMA)//BP86/Def2-SVP) = -5061.67675593

Zero-point correction=	0.341862 (Hartree/Particle)
Thermal correction to Energy=	0.370415
Thermal correction to Enthalpy=	0.371359
Thermal correction to Gibbs Free Energy=	0.276706

Charge = 0 Multiplicity = 1

Co	0.20586100	-0.03763400	-0.72805300
C	-2.94465900	-1.00365900	3.48804200
C	-2.71935900	-1.96172500	2.47760200
C	-2.42632000	-1.56277900	1.16191500
C	-2.33870200	-0.18794800	0.87121700
C	-2.58935900	0.78260900	1.85928900
H	-2.77311500	-3.03743300	2.71451500
H	-2.22659200	-2.30713200	0.37359700
H	-2.55648700	1.85582800	1.61142800
C	-2.88225000	0.36558000	3.17238000
H	-3.07326200	1.12271600	3.95145800
O	1.96849900	-0.38728500	-0.05691600
C	2.41472400	-1.34088700	0.64204800
N	3.75969100	-1.60433000	0.60789700
C	1.52258700	-2.14794200	1.55796400
C	4.41119200	-2.63743600	1.39658300
C	4.62991500	-0.85582200	-0.29159300
H	1.49511000	-3.21762300	1.26975200
H	1.85087200	-2.06715700	2.61709700
H	0.49645400	-1.75066300	1.45200900
H	5.18596600	-2.19825900	2.06564200
H	3.68358900	-3.18391100	2.02001500
H	4.91762900	-3.37269000	0.73172600
H	5.17043400	-1.55154100	-0.96998600
H	4.01225500	-0.16430000	-0.89065200
H	5.38579400	-0.27387300	0.28214500
O	0.59559400	1.81729200	-0.54692400
C	1.04535300	2.49589000	0.41792100
N	1.56533900	3.74464400	0.16864500
C	1.01176000	1.96078800	1.83059400
C	2.07912200	4.63146300	1.19821600
C	1.52609200	4.29377900	-1.18166000

H	2.02279600	1.67318000	2.18986200
H	0.58271400	2.68712600	2.55086000
H	0.37788600	1.05322700	1.80458000
H	1.38909600	5.48637900	1.39117400
H	2.23935500	4.09307800	2.14871500
H	3.05618600	5.05704100	0.87913600
H	0.86290300	5.18697000	-1.22717700
H	2.54451000	4.60225000	-1.50514600
H	1.13849900	3.52157900	-1.86910500
I	-2.15539400	0.46957000	-1.26948400
Br	0.05275800	-2.26211500	-1.52498900
H	-3.18002700	-1.32557000	4.51487300

VII

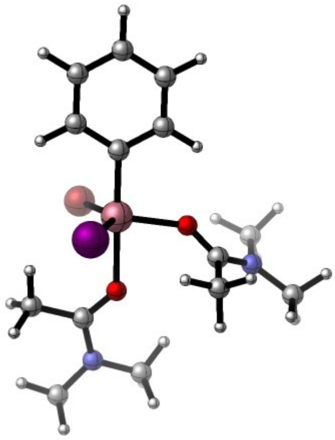

E (BP86/Def2-SVP) = -5061.60635173
E (BP86/Def2-TZVPP/SMD(DMA)//BP86/Def2-SVP) = -5063.04857614
E (M06/Def2-TZVPP/SMD(DMA)//BP86/Def2-SVP) = -5061.71648894

Zero-point correction=	0.343217 (Hartree/Particle)
Thermal correction to Energy=	0.372533
Thermal correction to Enthalpy=	0.373477
Thermal correction to Gibbs Free Energy=	0.275405

Charge = 0 Multiplicity = 3

Co	0.45025500	-0.08493700	0.20524200
C	5.07035000	0.90467500	0.55028700
C	4.60500400	-0.39313300	0.83402500
C	3.23690600	-0.69743800	0.73192600
C	2.31419200	0.29882200	0.34340900
C	2.78027500	1.59859100	0.06105400
H	5.31302800	-1.18122600	1.13977700
H	2.89111800	-1.71696600	0.96173000
H	2.07629500	2.38925600	-0.23752200
Br	0.28367600	-0.56335900	2.51307800
C	4.15408500	1.89664300	0.16487800
H	4.50446400	2.91825300	-0.05922200
O	-1.65938400	-0.47308100	0.05836500
C	-2.33290800	-1.49221500	0.33725300
N	-3.70012800	-1.39512300	0.42174500

C	-1.69473200	-2.84340100	0.58783800
C	-4.59310900	-2.51458400	0.68346100
C	-4.35669000	-0.11010700	0.22002300
H	-1.90245100	-3.19945900	1.61720800
H	-2.05828400	-3.60256100	-0.13489300
H	-0.60339100	-2.73938000	0.46810300
H	-5.28865700	-2.67358300	-0.17135200
H	-4.02937700	-3.44784000	0.84743200
H	-5.20900200	-2.31789700	1.58918600
H	-4.95318000	-0.10816500	-0.72011500
H	-5.04475100	0.10387400	1.06638900
H	-3.58610000	0.67837700	0.16519300
O	-0.20867800	1.81216500	-0.07086600
C	-1.08684500	2.37323100	-0.77213100
N	-1.75038100	3.45171200	-0.25666600
C	-1.39620600	1.90357700	-2.17724000
C	-2.65408100	4.30085200	-1.02049900
C	-1.45539100	3.89704900	1.10538700
H	-2.47974800	1.73481300	-2.33653100
H	-1.04804800	2.64694200	-2.92535500
H	-0.85539700	0.95306700	-2.34790500
H	-2.22688300	5.32141500	-1.14455300
H	-2.84528600	3.88227300	-2.02319900
H	-3.62667800	4.40397800	-0.49182500
H	-0.83839300	4.82281800	1.09673900
H	-2.40428800	4.11405300	1.63890200
H	-0.90060600	3.10142000	1.63354500
I	0.83847400	-1.41616700	-1.97576300
H	6.14387100	1.14009100	0.63090600

VIII

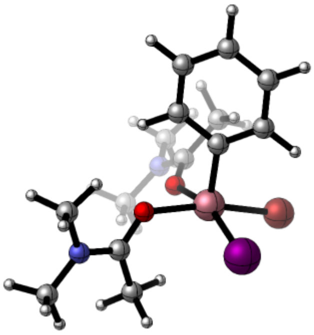

E (BP86/Def2-SVP) = -5061.60068385
E (BP86/Def2-TZVPP/SMD(DMA)//BP86/Def2-SVP) = -5063.04847411
E (M06/Def2-TZVPP/SMD(DMA)//BP86/Def2-SVP) = -5061.73103107

Zero-point correction=	0.344084 (Hartree/Particle)
Thermal correction to Energy=	0.372866
Thermal correction to Enthalpy=	0.373811
Thermal correction to Gibbs Free Energy=	0.279359

Charge = 0 Multiplicity = 1

Co	-0.02688800	-0.15671800	0.27862800
C	-0.84226000	-2.73963700	-3.58166400
C	-0.62788000	-3.38556500	-2.35080000
C	-0.36526000	-2.64443000	-1.18240800
C	-0.31685100	-1.23273800	-1.23526400
C	-0.52961000	-0.58476700	-2.47311400
H	-0.65992600	-4.48644700	-2.29069500
H	-0.20178500	-3.16770700	-0.22960600
H	-0.49344900	0.51336200	-2.53653800
C	-0.79196500	-1.33612200	-3.63640100
H	-0.95655400	-0.81224200	-4.59364600
O	-1.49602900	1.13749900	0.01873700
C	-2.75312900	1.07560200	-0.07898600
N	-3.48141000	2.20288500	0.18838400
C	-3.48323300	-0.17391000	-0.51418500
C	-4.93395500	2.27858100	0.11331900
C	-2.81574000	3.43517200	0.59716800
H	-4.16158500	-0.53673500	0.28388400
H	-4.07817200	0.01514300	-1.43211200
H	-2.74201400	-0.96404200	-0.71808900
H	-5.25117300	3.02076800	-0.65314400
H	-5.37414300	1.30110300	-0.14373600
H	-5.35272100	2.60058100	1.09219600
H	-3.10565500	3.71138400	1.63476600
H	-1.72400000	3.28436200	0.55050400
H	-3.10356900	4.26954200	-0.07883400
O	1.07441700	1.13622800	-0.75025600
C	1.77305900	2.06183200	-0.26031500
N	2.70205400	2.67654300	-1.04769500
C	1.60515800	2.50019600	1.18065000
C	3.56982700	3.75960000	-0.60800700
C	2.90946300	2.22098500	-2.42142800
H	2.46696600	2.16350400	1.79286800
H	1.49900100	3.59958100	1.27952700
H	0.69723600	2.02051300	1.59126700
H	3.40944400	4.66989500	-1.22794900
H	3.38903200	4.01980600	0.44847300
H	4.63670200	3.46264900	-0.71107300
H	2.62899800	3.01799900	-3.14471800
H	3.97958900	1.96693600	-2.57649300
H	2.29032700	1.32482200	-2.60112500
I	2.14390700	-1.24776500	1.05201900
Br	-1.29160300	-1.28742400	1.88720900
H	-1.04488400	-3.32663900	-4.49203100

IX

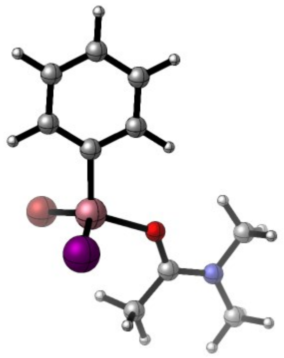

E (BP86/Def2-SVP) = -4773.97649560
E (BP86/Def2-TZVPP/SMD(DMA)//BP86/Def2-SVP) = -4775.09144495
E (M06/Def2-TZVPP/SMD(DMA)//BP86/Def2-SVP) = -4773.96782926

Zero-point correction=	0.215786 (Hartree/Particle)
Thermal correction to Energy=	0.235879
Thermal correction to Enthalpy=	0.236823
Thermal correction to Gibbs Free Energy=	0.159311

Charge = 0 Multiplicity = 3

Co	0.19981200	-0.05883000	0.35576400
C	3.82712000	2.32929800	-1.37942400
C	4.05037400	1.44288900	-0.30927200
C	2.98952000	0.69329200	0.23142500
C	1.69934500	0.84645800	-0.31029400
C	1.46208400	1.72373300	-1.38468000
H	5.06118100	1.33007900	0.11579600
H	3.17092700	0.00441300	1.07013800
H	0.45475300	1.82723400	-1.81694200
Br	0.23257900	0.51877700	2.58570300
C	2.53562600	2.46720000	-1.91552900
H	2.35320800	3.15492700	-2.75772300
O	-1.48378800	0.73274300	-0.41028100
C	-2.68596700	0.42039500	-0.17375100
N	-3.67931100	1.11534800	-0.79116500
C	-2.98693900	-0.70806100	0.78230400
C	-5.10114200	0.79313900	-0.72436200
C	-3.34160300	2.25331300	-1.64796600
H	-2.32037900	-0.57255200	1.66074200
H	-4.03170700	-0.77068400	1.13194300
H	-2.69513500	-1.66788500	0.30472900
H	-5.51502700	0.72506600	-1.75313600
H	-5.27431100	-0.17537900	-0.22683200
H	-5.66392000	1.58284300	-0.17945900
H	-3.45594400	1.98845500	-2.72185700
H	-4.02084600	3.10063700	-1.41965900
H	-2.29672100	2.55458500	-1.46108200
I	0.54660700	-2.26040800	-0.76311100
H	4.66356300	2.91182300	-1.79732900

X

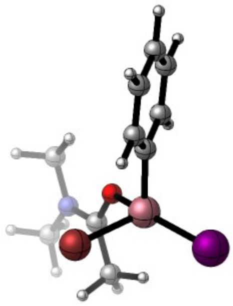

E (BP86/Def2-SVP) = -4773.95355963
E (BP86/Def2-TZVPP/SMD(DMA)//BP86/Def2-SVP) = -4775.07682817
E (M06/Def2-TZVPP/SMD(DMA)//BP86/Def2-SVP) = -4773.96782926

Zero-point correction=	0.216264 (Hartree/Particle)
Thermal correction to Energy=	0.235913
Thermal correction to Enthalpy=	0.236857
Thermal correction to Gibbs Free Energy=	0.162830

Charge = 0 Multiplicity = 1

Co	0.13655700	-0.22829500	-0.16963100
C	1.99651000	4.04414400	0.02257400
C	2.36688800	3.10773600	1.00655700
C	1.81736300	1.81336700	1.00836500
C	0.87587500	1.45510400	0.01994200
C	0.48747500	2.39509600	-0.96000100
H	3.10057900	3.38222100	1.78253800
H	2.12178300	1.08599300	1.77534300
H	-0.25789700	2.13795100	-1.72955700
Br	-0.34476900	-0.86304600	1.96895700
C	1.06184500	3.68232300	-0.96204600
H	0.76317700	4.40676800	-1.73811400
O	-1.66939100	0.45741300	-0.46703400
C	-2.71081500	-0.25900200	-0.54096700
N	-3.92687200	0.34681300	-0.49694000
C	-2.60596600	-1.76223500	-0.68083500
C	-5.20324100	-0.35316100	-0.57973600
C	-4.01535000	1.80090500	-0.35329800
H	-2.71427300	-2.24707400	0.31142300
H	-3.34814900	-2.18964600	-1.38207800
H	-1.59065300	-2.00532900	-1.05379500
H	-5.74278500	-0.08853200	-1.51618200
H	-5.06838900	-1.44713200	-0.54454400
H	-5.84536800	-0.05997200	0.27818000
H	-4.49345800	2.24906600	-1.25109900
H	-4.62987500	2.05472500	0.53616500
H	-2.99957600	2.21452600	-0.23049500
I	2.13337600	-1.50585400	-0.75972000
H	2.43552200	5.05459400	0.02542700

XII

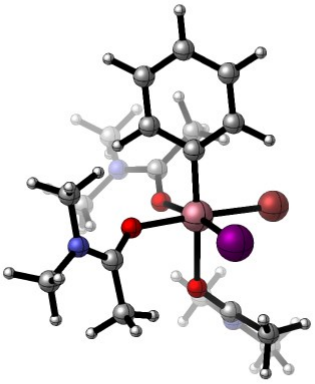

E (BP86/Def2-SVP) = -5349.22741350
E (BP86/Def2-TZVPP/SMD(DMA)//BP86/Def2-SVP) = -5350.99243400
E (M06/Def2-TZVPP/SMD(DMA)//BP86/Def2-SVP) = -5349.49561875

Zero-point correction=	0.471453 (Hartree/Particle)
Thermal correction to Energy=	0.509310
Thermal correction to Enthalpy=	0.510254
Thermal correction to Gibbs Free Energy=	0.396399

Charge = 0 Multiplicity = 1

Co	0.01898400	-0.04936100	-0.18123800
C	2.87000600	3.07481200	-2.39193900
C	2.30541700	2.01605200	-3.12629200
C	1.47432600	1.06675700	-2.50278400
C	1.17878500	1.15038900	-1.12094800
C	1.75840300	2.21787800	-0.39228700
H	2.51473100	1.91936400	-4.20515100
H	1.04454200	0.25387600	-3.10562800
H	1.56410500	2.31352500	0.68671700
C	2.59108600	3.16940800	-1.01842500
H	3.02523800	3.98899000	-0.41940100
O	-1.46433200	-1.20637900	0.98659800
C	-2.43127400	-1.90191800	0.59405800
N	-3.71470600	-1.50243500	0.86325200
C	-2.22720200	-3.21561500	-0.13416600
C	-4.90246600	-2.23319200	0.44756100
C	-3.95118000	-0.18708900	1.44434900
H	-2.54714500	-4.07564000	0.49324600
H	-2.79017000	-3.24433200	-1.08803400
H	-1.14881300	-3.31182000	-0.36401400
H	-5.36724000	-1.77206400	-0.45392300
H	-4.66538500	-3.28615100	0.21704100
H	-5.65593000	-2.22143700	1.26448700
H	-4.49940200	0.45927100	0.72387100
H	-4.55711400	-0.27353800	2.37328800
H	-2.97122300	0.27090200	1.66845100

O	1.26630100	0.00545500	1.40361400
C	1.78004600	-0.84867500	2.17262700
N	3.06460600	-0.66075400	2.59953800
C	1.00994400	-2.04763900	2.68337600
C	3.72993400	-1.49793000	3.58712500
C	3.87766500	0.41689900	2.04150900
H	1.54349300	-2.99546300	2.47246200
H	0.84339100	-1.96931500	3.77974900
H	0.03243000	-2.05581000	2.16977200
H	4.14219900	-0.87052300	4.40829700
H	3.03360100	-2.23020300	4.02918400
H	4.57733600	-2.05330800	3.12644900
H	4.04227400	1.22244400	2.79202200
H	4.86732500	0.01456200	1.73888500
H	3.36886600	0.84149900	1.15782200
O	-0.91846900	1.46852600	0.73715900
C	-1.27626800	2.64910200	0.49692500
N	-1.54506800	3.46931900	1.56673600
C	-1.42773000	3.20319500	-0.90120900
C	-2.01375600	4.84209100	1.45423000
C	-1.38387200	2.97008300	2.92847200
H	-2.42740500	3.66014200	-1.05076900
H	-0.65148300	3.96925600	-1.10565400
H	-1.31043500	2.36831900	-1.61697500
H	-1.33791700	5.52640600	2.01344500
H	-2.03935300	5.17128500	0.40210400
H	-3.03582900	4.95286300	1.88311600
H	-0.75309600	3.67035400	3.51777300
H	-2.36837000	2.87892400	3.43998600
H	-0.90038900	1.97806100	2.88803700
I	1.34834300	-2.11810100	-1.02953500
Br	-1.55834400	-0.16213500	-1.97977100
H	3.52142400	3.81502000	-2.88464700

TS4

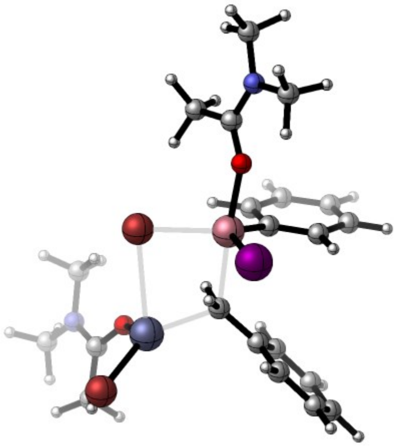

E (BP86/Def2-SVP) = -9685.94018720

E (BP86/Def2-TZVPP/SMD(DMA)//BP86/Def2-SVP) = -9688.25826499

E (M06/Def2-TZVPP/SMD(DMA)//BP86/Def2-SVP) = -9686.07640913

Zero-point correction= 0.457277 (Hartree/Particle)
Thermal correction to Energy= 0.498152
Thermal correction to Enthalpy= 0.499096
Thermal correction to Gibbs Free Energy= 0.367449

Charge = 0 Multiplicity = 3

Co	1.46390200	-0.05511400	-0.12831800
C	2.33943900	-0.74567500	4.47197300
C	1.65125300	-1.73041800	3.74161300
C	1.30841200	-1.50495700	2.39176100
C	1.65768200	-0.28873200	1.77017700
C	2.32977600	0.70675700	2.50570300
H	1.37081000	-2.68274300	4.22285000
H	0.76008200	-2.28217700	1.83230900
H	2.58387500	1.67175100	2.03680300
C	2.67529300	0.47131300	3.85112900
H	3.20489300	1.25445600	4.41863700
O	3.23400500	-0.83509200	-0.46875600
C	3.85298000	-1.92902000	-0.49415200
N	5.19449800	-1.90824500	-0.74218100
C	3.14783000	-3.24678000	-0.25855000
C	6.05587700	-3.08193900	-0.71543100
C	5.86559700	-0.63753000	-1.02059800
H	3.47637300	-4.03973100	-0.95806100
H	3.31997800	-3.59718200	0.78098700
H	2.06326400	-3.07722300	-0.39920000
H	6.89987800	-2.91671900	-0.01045900
H	5.50618100	-3.97891100	-0.38336700
H	6.48807100	-3.28193800	-1.72132900
H	6.46978800	-0.73076900	-1.94773000
H	5.10754000	0.15605900	-1.14968700
H	6.54458100	-0.36279900	-0.18383800
I	2.11682600	2.07489200	-1.55224500
Br	0.00780300	-1.61173400	-1.47069300
H	2.60406100	-0.92149200	5.52707400
C	-0.57076900	0.82950400	0.53526100
H	-0.16007200	1.04154900	-0.48244600
C	-0.76080700	1.95414400	1.46291300
C	-0.62991200	3.30094900	1.03085100
C	-1.11653600	1.72151200	2.81816900
C	-0.85283200	4.36450600	1.91720200
H	-0.34473600	3.50230900	-0.01398100
C	-1.34322100	2.78795200	3.69980500
H	-1.20642900	0.68337900	3.17768600
C	-1.21338400	4.11698700	3.25505100
H	-0.74497600	5.40064900	1.55782600
H	-1.61719900	2.58062500	4.74713800
H	-1.39034100	4.95512400	3.94792500
Zn	-2.22234200	0.19531500	-0.67270500
O	-3.16319500	-1.25757100	0.40258800
C	-4.35520400	-1.65021900	0.54181400

N	-4.58210000	-2.96007600	0.82890800
C	-5.49867400	-0.67188300	0.40991900
C	-5.88116900	-3.52808400	1.17269000
C	-3.45861900	-3.89941900	0.85647600
H	-6.46662900	-1.12805900	0.13594100
H	-5.62007300	-0.11417700	1.36299100
H	-5.21180100	0.06272700	-0.37332800
H	-5.80115700	-4.09100300	2.12735200
H	-6.64247100	-2.74121000	1.30548500
H	-6.22783600	-4.23446500	0.38658200
H	-3.21848700	-4.19560400	1.90104800
H	-3.72820100	-4.81202500	0.28524700
H	-2.57218600	-3.42181700	0.40296400
H	-0.65741800	-0.19024000	0.94061300
Br	-3.60516300	1.15278500	-2.28072700

TS5

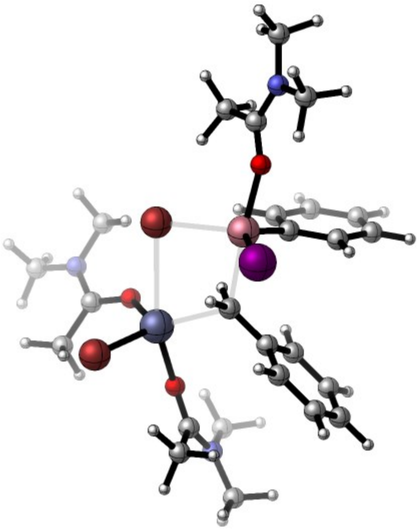

E (BP86/Def2-SVP) = -9973.57711788
E (BP86/Def2-TZVPP/SMD(DMA)//BP86/Def2-SVP) = -9976.21531825
E (M06/Def2-TZVPP/SMD(DMA)//BP86/Def2-SVP) = -9973.85658995

Zero-point correction=	0.584835 (Hartree/Particle)
Thermal correction to Energy=	0.634970
Thermal correction to Enthalpy=	0.635914
Thermal correction to Gibbs Free Energy=	0.485467

Charge = 0 Multiplicity = 3

Co	1.88123600	0.10524100	-0.09524800
C	2.38097100	1.46770100	4.41897200
C	1.84930900	0.19730100	4.13519200
C	1.62071300	-0.19613600	2.79974100
C	1.92547000	0.68168500	1.73759200
C	2.44560400	1.96080600	2.02546600
H	1.60386700	-0.49652100	4.95734700
H	1.19685200	-1.19354800	2.59135600
H	2.67071900	2.66565600	1.20771000
C	2.67651600	2.34663100	3.36035400

H	3.08640800	3.34848600	3.57188600
O	3.78485600	-0.39610400	-0.06387100
C	4.56233700	-1.26952000	0.39539300
N	5.90139000	-1.13078700	0.16397000
C	4.04578500	-2.44955600	1.18822000
C	6.92946200	-2.00022600	0.71667800
C	6.38649700	-0.01733600	-0.65236700
H	4.58163900	-3.39194600	0.96487800
H	4.12396200	-2.24281800	2.27658300
H	2.97543000	-2.57644700	0.93248100
H	7.68030100	-1.39531600	1.27114500
H	6.50108000	-2.73550700	1.41881300
H	7.46548500	-2.54894600	-0.09000700
H	7.06652000	-0.40213000	-1.44183400
H	5.52588100	0.49482600	-1.11996200
H	6.94944400	0.71037500	-0.02733000
I	2.30462400	1.56075800	-2.26355500
Br	0.79527000	-2.07855900	-0.62626800
H	2.55709500	1.77581300	5.46234200
C	-0.32643900	0.78609400	0.06406800
H	-0.03593900	0.53808300	-0.97755600
C	-0.70643400	2.16724000	0.38053400
C	-0.77284100	3.16578700	-0.62938500
C	-1.02258300	2.55389200	1.71080100
C	-1.14807100	4.48274100	-0.32139100
H	-0.50302700	2.89581000	-1.66273400
C	-1.40015800	3.87046100	2.01485100
H	-0.95224000	1.80449600	2.51546700
C	-1.46942100	4.84552900	1.00067500
H	-1.17942100	5.23809900	-1.12369400
H	-1.62413800	4.14450100	3.05925500
H	-1.75375900	5.88257400	1.24163400
Zn	-1.80931400	-0.63444700	-0.54602700
O	-3.43488600	0.38948200	0.40692500
C	-4.09696600	1.42468800	0.14232200
N	-4.75537700	2.07564800	1.14867800
C	-4.19010000	1.96216400	-1.27013100
C	-5.45850700	3.34082000	0.98814000
C	-4.64393900	1.59331400	2.52142500
H	-5.23375400	2.18505200	-1.56965500
H	-3.58979000	2.89097900	-1.36630800
H	-3.77520700	1.19714100	-1.95613200
H	-4.91407800	4.15702900	1.51290000
H	-5.54744200	3.61741200	-0.07563100
H	-6.48069000	3.27153400	1.42048100
H	-4.08311800	2.32352200	3.14469300
H	-5.65495300	1.45510800	2.96239400
H	-4.10059000	0.63250400	2.51621300
O	-2.20849200	-2.15749700	0.78403100
C	-3.01532800	-3.12261300	0.73514600
N	-2.69914200	-4.27337800	1.39524900

C	-4.30383300	-2.99671900	-0.04320200
C	-3.59759200	-5.40280900	1.59811100
C	-1.37880300	-4.39818500	2.01609700
H	-4.81919900	-3.94868800	-0.26024000
H	-4.99248300	-2.31878700	0.50207700
H	-4.05073600	-2.49418000	-1.00421300
H	-3.64618200	-5.65464100	2.67995400
H	-4.62115800	-5.16928100	1.25958600
H	-3.23843900	-6.30446300	1.05397300
H	-1.43317000	-4.22417100	3.11394800
H	-0.99157300	-5.42318700	1.84143000
H	-0.69081300	-3.66042800	1.56284500
H	-0.32148400	0.04866400	0.88316500
Br	-2.57870200	-1.09215300	-2.75945000

TS6

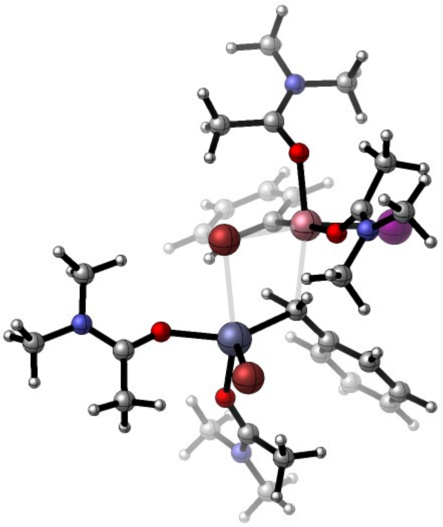

E (BP86/Def2-SVP) = -10261.1976068
E (BP86/Def2-TZVPP/SMD(DMA)//BP86/Def2-SVP) = -10264.1519050
E (M06/Def2-TZVPP/SMD(DMA)//BP86/Def2-SVP) = -10261.6170050

Zero-point correction=	0.712520 (Hartree/Particle)
Thermal correction to Energy=	0.771805
Thermal correction to Enthalpy=	0.772750
Thermal correction to Gibbs Free Energy=	0.604149

Charge = 0 Multiplicity = 3

Co	1.75625400	0.04777300	-0.02697600
C	1.60557800	1.18112500	-4.70530300
C	0.55791800	1.55506800	-3.84718600
C	0.57731200	1.18613400	-2.48672600
C	1.63998900	0.42256300	-1.93865500
C	2.68647700	0.05572900	-2.82183200
H	-0.28999700	2.14740200	-4.23176700

H	-0.24461900	1.54042900	-1.84332800
H	3.52255700	-0.55787800	-2.45316500
C	2.67141400	0.43039100	-4.18010200
H	3.50220100	0.11523900	-4.83451900
O	3.37162500	1.28944300	0.10389000
C	3.86738700	2.19876900	-0.61427500
N	5.17237600	2.10415600	-0.99954600
C	3.04592400	3.39728100	-1.03492700
C	5.88270000	3.13307900	-1.74567400
C	5.94493200	0.90523100	-0.67492400
H	3.48868100	4.34489500	-0.66248200
H	2.96269400	3.45589000	-2.13954400
H	2.03273200	3.28039200	-0.60419200
H	6.19468400	2.74840400	-2.74177400
H	5.24980600	4.02309300	-1.90042800
H	6.79906400	3.44603200	-1.19790000
H	6.65826000	1.10145800	0.15650800
H	5.25815300	0.08710300	-0.38486000
H	6.52643900	0.59099700	-1.56633700
O	1.70676200	-0.27437000	2.02134500
C	2.44555400	-0.29808300	3.03270700
N	1.86916800	-0.36912700	4.27049200
C	3.95422500	-0.24910800	2.91066600
C	2.59625200	-0.63280100	5.50292400
C	0.41120100	-0.31337700	4.38828200
H	4.44087800	0.32351400	3.72393000
H	4.36480300	-1.28057100	2.89047600
H	4.18529200	0.21705500	1.93663000
H	2.21301500	-1.56021800	5.98440800
H	3.67401600	-0.77385100	5.31230300
H	2.46992700	0.20136100	6.22917600
H	-0.03789000	-1.33045600	4.42487200
H	0.14376600	0.22105100	5.32256000
H	-0.02014300	0.22756600	3.52685400
I	3.08697700	-2.28228100	-0.23127600
Br	0.07429000	2.10063600	0.83370900
H	1.58976100	1.46669000	-5.76996500
C	-0.43287900	-0.94203500	-0.06158600
H	-0.12860500	-0.74266200	0.97765400
C	-0.66073900	-2.32743700	-0.45934800
C	-0.67455600	-3.37447900	0.50419000
C	-0.88734400	-2.68744800	-1.81703500
C	-0.91407500	-4.70459300	0.13310800
H	-0.49020600	-3.12450800	1.56115100
C	-1.12283200	-4.01937600	-2.18714700
H	-0.85086800	-1.90022900	-2.58722400
C	-1.14408900	-5.04004700	-1.21589600
H	-0.91135800	-5.49331000	0.90342400
H	-1.27158600	-4.27004400	-3.25094100
H	-1.31895600	-6.08761100	-1.50919100
Zn	-2.10484000	0.36720200	0.52892700

O	-3.50632600	-0.60644700	-0.82308100
C	-4.04025200	-1.73807700	-0.90918300
N	-4.49654100	-2.17841100	-2.12465100
C	-4.21571700	-2.62616200	0.30473200
C	-5.03208100	-3.50986600	-2.36571400
C	-4.28407900	-1.36295200	-3.31505000
H	-5.26373900	-2.97060800	0.42138800
H	-3.55726000	-3.51687100	0.23154800
H	-3.92667300	-2.04203500	1.20019700
H	-4.30894600	-4.12679600	-2.94522600
H	-5.24405100	-4.03347400	-1.41836000
H	-5.97664600	-3.44358100	-2.94889600
H	-3.56034400	-1.85430400	-4.00250100
H	-5.24227600	-1.22010000	-3.86053500
H	-3.87815300	-0.38408200	-3.00497600
O	-2.89643400	2.06696600	-0.38438700
C	-3.90063500	2.78723200	-0.16538900
N	-3.84285400	4.11199400	-0.49549500
C	-5.14959500	2.19714900	0.45116500
C	-4.97721000	5.02494300	-0.50435500
C	-2.57259500	4.67261400	-0.96005200
H	-5.82566700	2.93083500	0.92595700
H	-5.70871600	1.63064400	-0.32240800
H	-4.81413200	1.46569300	1.21848900
H	-5.07324600	5.50127500	-1.50504500
H	-5.92127000	4.49523200	-0.29095000
H	-4.84671800	5.83631500	0.24625700
H	-2.55227500	4.76123300	-2.06946300
H	-2.44383500	5.68557600	-0.52535600
H	-1.74081100	4.01931900	-0.63347200
H	-0.36850200	-0.17755500	-0.84914400
Br	-3.07234200	0.07842600	2.73132300

XIII

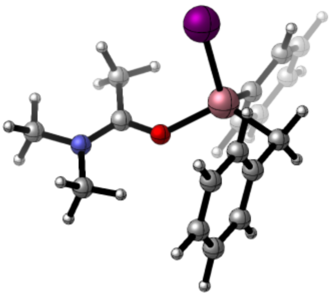

E (BP86/Def2-SVP) = -2470.57575974
E (BP86/Def2-TZVPP/SMD(DMA)//BP86/Def2-SVP) = -2471.669451
E (M06/Def2-TZVPP/SMD(DMA)//BP86/Def2-SVP) = -2470.702157

Zero-point correction=	0.329066 (Hartree/Particle)
Thermal correction to Energy=	0.354102
Thermal correction to Enthalpy=	0.355046
Thermal correction to Gibbs Free Energy=	0.266358

Charge = 0 Multiplicity = 3

Co	0.40543300	-0.45559500	-0.15952100
C	4.66711900	0.18491400	-2.08502400
C	3.52483000	0.79437100	-2.63382400
C	2.25856700	0.57426300	-2.05740900
C	2.12003100	-0.25592500	-0.91879200
C	3.27294300	-0.87414700	-0.38071200
H	3.61641400	1.44612500	-3.51899600
H	1.37341400	1.05907400	-2.50391800
H	3.18854500	-1.52479900	0.50499100
C	4.53609800	-0.64833500	-0.95828900
H	5.42707300	-1.12905500	-0.52106600
I	0.46537100	-0.60045100	2.41402000
H	5.65787300	0.35665000	-2.53572700
C	0.03044200	-2.09847200	-1.15238200
H	0.44462400	-2.88185800	-0.48515700
C	-1.44068200	-1.98954500	-1.17009800
C	-2.12723900	-1.52450800	-2.32824000
C	-2.21930900	-2.28863000	-0.01679800
C	-3.52017200	-1.37653600	-2.33486500
H	-1.54487100	-1.29146800	-3.23505700
C	-3.61415200	-2.13827600	-0.02666100
H	-1.71154600	-2.64186700	0.89496600
C	-4.27324000	-1.68113200	-1.18247400
H	-4.02880500	-1.03070500	-3.24966400
H	-4.19320900	-2.38389700	0.87806200
H	-5.36932400	-1.56954600	-1.19022600
H	0.50837800	-2.12401600	-2.14974100
O	-0.66890200	1.19529500	-0.50148600
C	-0.61909600	2.37325700	-0.05215700
N	-1.72626500	3.16157000	-0.14119600
C	0.64397300	2.91875000	0.57885300
C	-1.80723000	4.52531200	0.36618100
C	-2.95343400	2.64143700	-0.74368600
H	0.92967700	3.90588500	0.16354400
H	0.52145900	3.01617500	1.67759200
H	1.46166600	2.19885700	0.39402100
H	-2.63924600	4.60989900	1.09876200
H	-0.87367300	4.82359600	0.87172400
H	-2.00757600	5.24176800	-0.46107100
H	-3.77604000	2.64055700	0.00336300
H	-3.25827600	3.28043200	-1.60033900
H	-2.77955900	1.60899900	-1.09356500

XIV

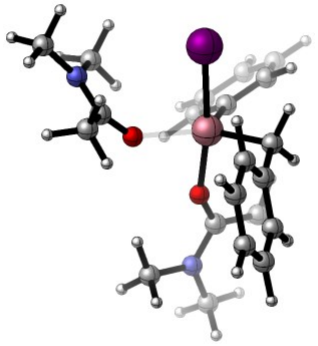

E (BP86/Def2-SVP) = -2758.20204461
E (BP86/Def2-TZVPP/SMD(DMA)//BP86/Def2-SVP) = -2759.61845597
E (M06/Def2-TZVPP/SMD(DMA)//BP86/Def2-SVP) = -2758.45653196

Zero-point correction= 0.457756 (Hartree/Particle)
Thermal correction to Energy= 0.491248
Thermal correction to Enthalpy= 0.492192
Thermal correction to Gibbs Free Energy= 0.388111

Charge = 0 Multiplicity = 1

Co	-0.00756600	0.12413700	0.26666800
C	-0.85740600	4.69324800	1.04547800
C	-0.57029100	4.17560900	-0.23004500
C	-0.31742600	2.79996000	-0.39958700
C	-0.35745900	1.90338100	0.70095500
C	-0.65245100	2.43991800	1.97772800
H	-0.53820900	4.84689400	-1.10553800
H	-0.07560700	2.42251900	-1.40773400
H	-0.70977400	1.77639900	2.85440100
C	-0.89460400	3.81629300	2.14562100
H	-1.12565800	4.20513700	3.15187700
O	-0.60968900	-0.46565200	-1.66812000
C	-1.56000300	-1.13167100	-2.15088500
N	-2.73436700	-0.54582800	-2.52101600
C	-1.40119200	-2.63121100	-2.32907500
C	-3.91702200	-1.28907300	-2.93537800
C	-2.95387700	0.87874900	-2.28502700
H	-2.04542000	-3.16731500	-1.60033200
H	-1.65837700	-2.98122900	-3.34962900
H	-0.34929700	-2.88661900	-2.11004800
H	-4.40127100	-0.77735600	-3.79433100
H	-3.66257100	-2.31709300	-3.24512100
H	-4.65638200	-1.34497200	-2.10472000
H	-3.69659000	1.02498700	-1.47160200
H	-2.00803800	1.34945700	-1.96959600
H	-3.32809000	1.36107600	-3.21377000
O	1.65267400	0.48996000	-0.67663700
C	2.66708400	1.23216300	-0.69430700
N	3.55046000	1.08735900	-1.73238000
C	2.95207600	2.26918000	0.37201700
C	4.80996700	1.80646500	-1.84694000
C	3.28713300	0.09917600	-2.77628200

H	3.88071900	2.02709700	0.93064900
H	3.06727800	3.28163900	-0.06550700
H	2.10119600	2.29585400	1.07367900
H	4.86032300	2.35151000	-2.81560700
H	4.93049800	2.54006100	-1.03207800
H	5.67140700	1.10190900	-1.81094300
H	3.44263700	0.56019200	-3.77487800
H	3.97239600	-0.77210700	-2.68061900
H	2.24444400	-0.25279300	-2.68257700
I	-2.29244200	-0.67934100	1.18966800
H	-1.05352200	5.76903400	1.18126500
C	0.92533400	-0.32446400	1.92516300
H	0.18170500	-0.44482200	2.73669800
C	1.60749400	-1.60018200	1.57360900
C	3.00397000	-1.64944900	1.31263300
C	0.88613600	-2.82099600	1.46593300
C	3.64725800	-2.84657800	0.96544200
H	3.59664200	-0.72510100	1.41569400
C	1.52813800	-4.01994300	1.11691600
H	-0.19766000	-2.81329000	1.66720200
C	2.91110800	-4.04175300	0.85964000
H	4.73516000	-2.85200000	0.78550400
H	0.94182700	-4.95136700	1.05241100
H	3.41422600	-4.98383900	0.58843400
H	1.64934700	0.47143100	2.19456700

XV

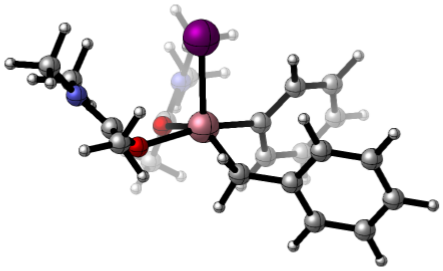

E (BP86/Def2-SVP) = -2758.19012026
E (BP86/Def2-TZVPP/SMD(DMA)//BP86/Def2-SVP) = -2759.60710871
E (M06/Def2-TZVPP/SMD(DMA)//BP86/Def2-SVP) = -2758.46725072

Zero-point correction= 0.456422 (Hartree/Particle)
Thermal correction to Energy= 0.490500
Thermal correction to Enthalpy= 0.491444
Thermal correction to Gibbs Free Energy= 0.384096

Charge = 0 Multiplicity = 3

Co	0.40543300	-0.45559500	-0.15952100
C	4.66711900	0.18491400	-2.08502400
C	3.52483000	0.79437100	-2.63382400
C	2.25856700	0.57426300	-2.05740900
C	2.12003100	-0.25592500	-0.91879200
C	3.27294300	-0.87414700	-0.38071200
H	3.61641400	1.44612500	-3.51899600

H	1.37341400	1.05907400	-2.50391800
H	3.18854500	-1.52479900	0.50499100
C	4.53609800	-0.64833500	-0.95828900
H	5.42707300	-1.12905500	-0.52106600
I	0.46537100	-0.60045100	2.41402000
H	5.65787300	0.35665000	-2.53572700
C	0.03044200	-2.09847200	-1.15238200
H	0.44462400	-2.88185800	-0.48515700
C	-1.44068200	-1.98954500	-1.17009800
C	-2.12723900	-1.52450800	-2.32824000
C	-2.21930900	-2.28863000	-0.01679800
C	-3.52017200	-1.37653600	-2.33486500
H	-1.54487100	-1.29146800	-3.23505700
C	-3.61415200	-2.13827600	-0.02666100
H	-1.71154600	-2.64186700	0.89496600
C	-4.27324000	-1.68113200	-1.18247400
H	-4.02880500	-1.03070500	-3.24966400
H	-4.19320900	-2.38389700	0.87806200
H	-5.36932400	-1.56954600	-1.19022600
H	0.50837800	-2.12401600	-2.14974100
O	-0.66890200	1.19529500	-0.50148600
C	-0.61909600	2.37325700	-0.05215700
N	-1.72626500	3.16157000	-0.14119600
C	0.64397300	2.91875000	0.57885300
C	-1.80723000	4.52531200	0.36618100
C	-2.95343400	2.64143700	-0.74368600
H	0.92967700	3.90588500	0.16354400
H	0.52145900	3.01617500	1.67759200
H	1.46166600	2.19885700	0.39402100
H	-2.63924600	4.60989900	1.09876200
H	-0.87367300	4.82359600	0.87172400
H	-2.00757600	5.24176800	-0.46107100
H	-3.77604000	2.64055700	0.00336300
H	-3.25827600	3.28043200	-1.60033900
H	-2.77955900	1.60899900	-1.09356500

TS8

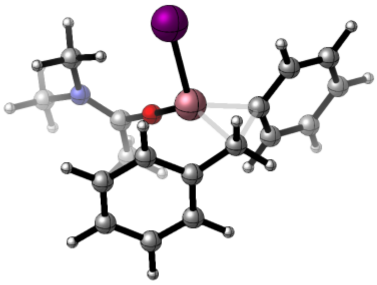

E (BP86/Def2-SVP) = -2470.56110440
E (BP86/Def2-TZVPP/SMD(DMA)//BP86/Def2-SVP) = -2471.64787941
E (M06/Def2-TZVPP/SMD(DMA)//BP86/Def2-SVP) = -2470.69220758

Zero-point correction=	0.329042 (Hartree/Particle)
Thermal correction to Energy=	0.352993
Thermal correction to Enthalpy=	0.353937
Thermal correction to Gibbs Free Energy=	0.270230

Charge = 0 Multiplicity = 3

Co	0.36321700	-0.01889300	0.24987900
C	3.76508800	-2.30240600	-1.95358800
C	2.57954100	-2.95936600	-1.57268300
C	1.69403200	-2.35390700	-0.66566300
C	1.95904100	-1.05925400	-0.14802100
C	3.16769200	-0.41901800	-0.51510400
H	2.34288400	-3.95555500	-1.98215400
H	0.77822900	-2.88943000	-0.36352700
H	3.39870000	0.57899500	-0.11108900
C	4.05296900	-1.03367800	-1.41808200
H	4.97850600	-0.50925700	-1.70752600
I	0.93351200	2.54545900	0.18080600
H	4.46303700	-2.78053000	-2.65915200
C	1.48157800	-0.83904500	1.82171500
H	1.94858100	0.07479700	2.23097000
C	0.05915400	-1.06209200	2.14540400
C	-0.52272100	-2.37101800	2.14279100
C	-0.81534000	0.06224400	2.32219100
C	-1.89932700	-2.54576900	2.28788000
H	0.13862200	-3.24718600	2.04571400
C	-2.20989200	-0.13740600	2.46442900
H	-0.39229400	1.06565400	2.48295700
C	-2.75466400	-1.42524100	2.43404100
H	-2.32204400	-3.56351800	2.30078900
H	-2.86158900	0.73639700	2.62523900
H	-3.83956200	-1.57294100	2.55744700
H	2.12086800	-1.72294100	1.97502400
O	-1.16889500	-0.53133600	-0.92911900
C	-2.27832200	-0.67477800	-1.49203900
N	-3.07391300	0.39122900	-1.79525400
C	-2.74304300	-2.07512300	-1.86087800
C	-4.35878500	0.28481000	-2.47403700
C	-2.65316200	1.75469800	-1.47307300

H	-3.70359100	-2.33597600	-1.37109800
H	-2.87302600	-2.19122200	-2.95688300
H	-1.96915700	-2.78454100	-1.51980300
H	-4.33422600	0.83281300	-3.44215400
H	-4.62738100	-0.76562800	-2.67701800
H	-5.16279000	0.73656900	-1.85202200
H	-2.55169100	2.35439600	-2.40337500
H	-3.41108500	2.24313300	-0.82372700
H	-1.67846700	1.74691200	-0.95004600

TS9

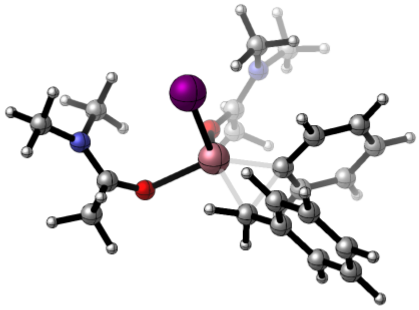

E (BP86/Def2-SVP) = -2758.17839155
E (BP86/Def2-TZVPP/SMD(DMA)//BP86/Def2-SVP) = -2759.59446886
E (M06/Def2-TZVPP/SMD(DMA)//BP86/Def2-SVP) = -2758.46020928

Zero-point correction=	0.455965 (Hartree/Particle)
Thermal correction to Energy=	0.489468
Thermal correction to Enthalpy=	0.490413
Thermal correction to Gibbs Free Energy=	0.384743

Charge = 0 Multiplicity = 3

Co	-0.30417300	0.02144200	-0.56085100
C	3.36439800	2.86545700	0.19483800
C	2.78963800	2.78433500	-1.08751200
C	1.88168400	1.75579400	-1.39592000
C	1.49370200	0.79542300	-0.41976800
C	2.12126200	0.86420800	0.85348500
H	3.06277400	3.51956700	-1.86349200
H	1.45464500	1.70349600	-2.41246600
H	1.87859400	0.10519600	1.61408900
C	3.02551700	1.89352400	1.15849000
H	3.48002100	1.93201000	2.16268800
O	-1.19357500	1.86798200	-0.77010300
C	-1.03138400	3.09697100	-0.55614300
N	-0.75571600	3.60111400	0.67614800
C	-1.15850400	4.05351700	-1.73211600
C	-0.46378100	5.00523400	0.93762600
C	-0.70249600	2.73437600	1.85307000
H	-1.85633700	4.89289600	-1.53709300
H	-0.16768900	4.47988100	-1.99621200
H	-1.52538100	3.47139500	-2.59593800
H	0.56857800	5.10943800	1.33761700
H	-0.54297900	5.61679700	0.02329800

H	-1.16789200	5.41167100	1.69662800
H	-1.50667500	3.01703400	2.56730000
H	-0.83093000	1.67645400	1.55789500
H	0.27951200	2.84228400	2.35736300
O	-1.80338700	-0.79117900	-1.88695600
C	-2.65617000	-1.66240600	-1.57387800
N	-3.81604200	-1.32603100	-0.94012100
C	-2.38394600	-3.12222200	-1.88736900
C	-4.71614500	-2.29086900	-0.32142000
C	-4.06420500	0.06914800	-0.58619300
H	-2.04365800	-3.63807600	-0.96295300
H	-3.26128200	-3.66654600	-2.28953900
H	-1.56472100	-3.16139100	-2.62780100
H	-5.77105700	-1.99637000	-0.50724100
H	-4.56271900	-3.30513100	-0.72818500
H	-4.54765500	-2.32690300	0.77851900
H	-5.12134700	0.32491200	-0.81272100
H	-3.86969300	0.23183000	0.49673600
H	-3.37987600	0.71727400	-1.16121000
I	-1.10216100	-1.19659500	1.74041800
H	4.08972600	3.65994400	0.43288800
C	1.32842800	-0.96883300	-1.25231200
H	1.42671100	-0.70660200	-2.32511000
C	2.55603500	-1.66380800	-0.74962600
C	2.50177700	-2.51373400	0.38062000
C	3.79568500	-1.50144400	-1.41125000
C	3.65252200	-3.18196100	0.82887200
H	1.54341000	-2.63880200	0.91269400
C	4.94531400	-2.17036000	-0.96271900
H	3.85448400	-0.83654900	-2.28886300
C	4.87897600	-3.01461000	0.16044300
H	3.58932700	-3.84146200	1.70958000
H	5.90073000	-2.03206000	-1.49440100
H	5.78056400	-3.54090100	0.51297800
H	0.46618900	-1.68711700	-1.13924200

TS10

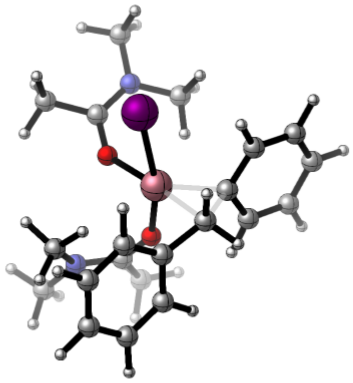

E (BP86/Def2-SVP) = -5034.43966907

E (BP86/Def2-TZVPP/SMD(DMA)//BP86/Def2-SVP) = -2759.5656

E (M06/Def2-TZVPP/SMD(DMA)//BP86/Def2-SVP) = -2758.41803247

Zero-point correction= 0.458081 (Hartree/Particle)
Thermal correction to Energy= 0.490320
Thermal correction to Enthalpy= 0.491265
Thermal correction to Gibbs Free Energy= 0.392656

Charge = 0 Multiplicity = 1

Co	0.04535300	0.21564000	-0.02490200
C	2.30651600	3.71189900	2.01715400
C	0.97163900	3.43141900	2.37899600
C	0.14561600	2.66433900	1.55137900
C	0.62107300	2.07667700	0.32570400
C	1.95623700	2.44902200	-0.04947700
H	0.56143200	3.83305200	3.32135100
H	-0.89633500	2.49189900	1.85481200
H	2.35200900	2.10280700	-1.01613300
C	2.77666200	3.22354400	0.78365500
H	3.80358100	3.45492000	0.45337900
O	0.41505000	-1.58166000	0.63889100
C	1.42181500	-2.32350300	0.81566100
N	2.60521000	-1.87048600	1.30249800
C	1.26694000	-3.79708300	0.49099600
C	3.83246900	-2.65852900	1.29712200
C	2.78365800	-0.46803600	1.67235300
H	1.76399900	-4.01702300	-0.47776200
H	1.68349200	-4.47038400	1.26642700
H	0.18901600	-4.00313700	0.36828900
H	4.34172300	-2.57398600	2.28071100
H	3.63340700	-3.72448800	1.09544900
H	4.52596200	-2.27822100	0.51414400
H	3.44642600	0.04616100	0.94497200
H	1.80640500	0.05040400	1.65932500
H	3.23475200	-0.40979100	2.68562700
O	-1.26249800	0.23470300	1.46079600
C	-2.10608500	-0.47479100	2.06169300
N	-2.74199000	-1.55277200	1.51749600
C	-2.44665000	-0.07977300	3.49317200
C	-3.71640800	-2.36180500	2.23993200
C	-2.57657700	-1.92110800	0.11502600
H	-3.52696700	0.13769500	3.62391600
H	-2.16916600	-0.87566700	4.21589700
H	-1.86466100	0.82590500	3.73820100
H	-3.48086600	-3.43992100	2.10531900
H	-3.71192700	-2.14410300	3.32129600
H	-4.74495300	-2.19156400	1.84853400
H	-2.16550800	-2.94921200	0.02938200
H	-3.55662000	-1.87594900	-0.40459600
H	-1.88361900	-1.21611600	-0.37827600
I	1.65572200	-0.46627900	-2.02892500
H	2.95228600	4.32103600	2.66914300
C	-0.47283100	2.10413300	-1.04485300
H	0.17487600	2.08765000	-1.93978500

C	-1.76547700	1.35828800	-1.30581100
C	-2.94654400	1.67924200	-0.57382300
C	-1.92133400	0.53321700	-2.45576100
C	-4.19603700	1.15606200	-0.93551500
H	-2.87533900	2.37050400	0.28136700
C	-3.17523300	0.02178200	-2.82168600
H	-1.03554400	0.30337100	-3.06870900
C	-4.32421600	0.31906700	-2.06188500
H	-5.08951600	1.43275000	-0.35094500
H	-3.25716500	-0.60785400	-3.72295500
H	-5.31029600	-0.07249500	-2.35912600
H	-0.75305500	3.15636600	-0.85551700

XVI

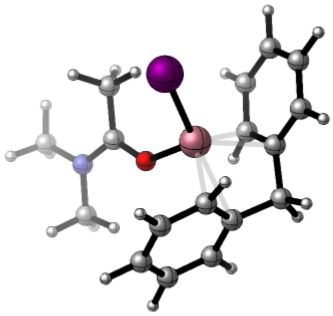

E (BP86/Def2-SVP) = -2470.60087840
E (BP86/Def2-TZVPP/SMD(DMA)//BP86/Def2-SVP) = -2471.69355064
E (M06/Def2-TZVPP/SMD(DMA)//BP86/Def2-SVP) = -2470.75160855

Zero-point correction=	0.330689 (Hartree/Particle)
Thermal correction to Energy=	0.354882
Thermal correction to Enthalpy=	0.355826
Thermal correction to Gibbs Free Energy=	0.272418

Charge = 0 Multiplicity = 3

Co	0.33955200	0.29431900	-0.01687600
C	2.60187800	-0.92123100	2.50732900
C	1.24638800	-0.61630300	2.64776100
C	0.70824100	0.59396200	2.12078600
C	1.57963800	1.51381200	1.44535500
C	2.96100400	1.18140800	1.32957100
H	0.58325000	-1.30304000	3.19839700
H	-0.30309900	0.91371200	2.41927400
H	3.63675400	1.89074000	0.82406800
C	3.46472500	-0.01279900	1.84883300
H	4.53343500	-0.25208900	1.73555500
I	1.57267700	-1.41676200	-1.50586100
H	3.00016300	-1.86584400	2.90933500
C	1.03576000	2.87487900	1.03086600
H	1.84959300	3.49090200	0.59031400
C	-0.07931300	2.65420000	0.02146900
C	-1.41454200	3.07096000	0.25840200
C	0.24842100	2.05961400	-1.24500700
C	-2.40382800	2.92051400	-0.72015900

H	-1.66175100	3.54282400	1.22426900
C	-0.77437500	1.92147000	-2.23248200
H	1.30319900	1.97945800	-1.55928300
C	-2.08156900	2.33190300	-1.96917700
H	-3.42868300	3.27507200	-0.52448700
H	-0.51099900	1.49468000	-3.21298900
H	-2.85897100	2.21936800	-2.74191900
H	0.65547900	3.43073300	1.91537200
O	-1.59465100	-0.24967100	0.43069800
C	-2.18055300	-1.34915800	0.59474800
N	-3.54761200	-1.37755900	0.67517900
C	-1.41228800	-2.64999600	0.71126700
C	-4.33425800	-2.58375500	0.88844400
C	-4.31506600	-0.14226300	0.54975300
H	-1.53436200	-3.10514000	1.71648300
H	-1.73966300	-3.39157700	-0.04546700
H	-0.34232200	-2.43196200	0.53046900
H	-5.02069200	-2.76205300	0.03067800
H	-3.68879300	-3.47042900	1.00529000
H	-4.95807300	-2.48408900	1.80466500
H	-5.05578100	-0.23374500	-0.27408400
H	-4.86784200	0.07170000	1.49127300
H	-3.62173200	0.68952100	0.33069100

XVII

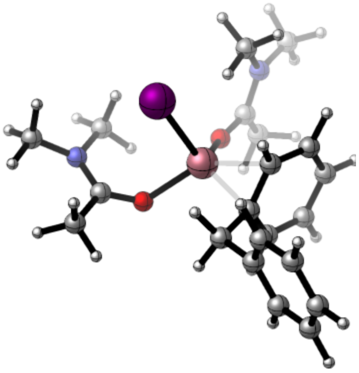

E (BP86/Def2-SVP) = -2758.19872136
E (BP86/Def2-TZVPP/SMD(DMA)//BP86/Def2-SVP) = -2759.64607223
E (M06/Def2-TZVPP/SMD(DMA)//BP86/Def2-SVP) = -2758.51680138

Zero-point correction= 0.455949 (Hartree/Particle)
Thermal correction to Energy= 0.490678
Thermal correction to Enthalpy= 0.491622
Thermal correction to Gibbs Free Energy= 0.378886

Charge = 0 Multiplicity = 3

Co	-0.38222000	0.06837300	-0.32085400
C	0.73325400	3.46700000	-0.25429300
C	1.06726300	2.94491300	-1.53397100
C	1.45633800	1.61369600	-1.67464600
C	1.51714900	0.71688500	-0.54676100
C	1.12552500	1.25125600	0.74258100

H	1.03624400	3.59946800	-2.42040100
H	1.74908500	1.23151600	-2.66700700
H	1.34273700	0.67821100	1.65742900
C	0.77226500	2.62733800	0.86116400
H	0.55499900	3.03401000	1.86286100
O	-1.81125000	1.42050800	-0.78920000
C	-2.45597300	2.47041300	-0.55855400
N	-2.87216100	2.81840300	0.69547400
C	-2.82290400	3.36896900	-1.73085200
C	-3.63041700	4.02386300	0.99928700
C	-2.58006500	1.96328500	1.84361500
H	-3.92191300	3.46510900	-1.85701300
H	-2.40077100	4.38832700	-1.61706600
H	-2.39753600	2.91366200	-2.64239300
H	-3.06300800	4.67517300	1.70117500
H	-3.85251000	4.60698600	0.08958400
H	-4.59459000	3.76231700	1.48964000
H	-3.51846900	1.53236500	2.25710900
H	-1.91011700	1.13607500	1.54062600
H	-2.08562200	2.55499300	2.64285700
O	-0.71872200	-1.30316500	-1.83188300
C	-1.16611300	-2.47635900	-1.71081700
N	-2.48417600	-2.70778700	-1.45895700
C	-0.21851400	-3.65061400	-1.84539800
C	-3.01226800	-3.99336000	-1.01786200
C	-3.38846500	-1.57259600	-1.29385100
H	0.05065800	-4.01622100	-0.83040400
H	-0.63311400	-4.49645500	-2.42864700
H	0.70341700	-3.28706500	-2.33370400
H	-3.98868900	-4.19025400	-1.50912900
H	-2.32351500	-4.81807500	-1.26883900
H	-3.16203200	-3.98881200	0.08531900
H	-4.38364500	-1.83747500	-1.70766600
H	-3.49078300	-1.31360200	-0.21631800
H	-2.97744500	-0.69476100	-1.82267300
I	-0.89147800	-1.60808000	1.74247700
H	0.47113500	4.53142900	-0.14179300
C	2.37402100	-0.55417700	-0.63171900
H	2.30548500	-0.95412000	-1.66533300
C	3.83296300	-0.31969000	-0.26363900
C	4.31430700	-0.65333300	1.02104700
C	4.73326100	0.25984800	-1.18504400
C	5.65314000	-0.41309700	1.37685900
H	3.62725300	-1.11391500	1.75024600
C	6.07185400	0.50107900	-0.83383000
H	4.37863500	0.52593500	-2.19456200
C	6.53736700	0.16620100	0.45058300
H	6.00789900	-0.68421800	2.38447200
H	6.75806300	0.95139700	-1.56946500
H	7.58750600	0.35336100	0.72666800
H	1.94913600	-1.32536900	0.04587700

PhBn

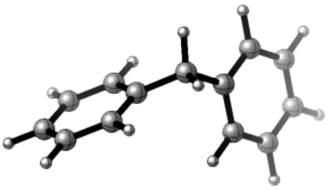

E (BP86/Def2-SVP) = -502.244150526
E (BP86/Def2-TZVPP/SMD(DMA)//BP86/Def2-SVP) = -502.793829659
E (M06/Def2-TZVPP/SMD(DMA)//BP86/Def2-SVP) = -502.395627521

Zero-point correction=	0.203568 (Hartree/Particle)
Thermal correction to Energy=	0.214211
Thermal correction to Enthalpy=	0.215155
Thermal correction to Gibbs Free Energy=	0.164731

Charge = 0 Multiplicity = 1

C	-3.68597300	0.88224500	0.14633300
C	-2.66050800	1.14490700	1.07374900
C	-1.47116500	0.39977600	1.04261000
C	-1.28233600	-0.62538600	0.08813700
C	-2.31702400	-0.87626300	-0.83730100
H	-2.78872700	1.93888000	1.82712600
H	-0.66924900	0.61781000	1.76710300
H	-2.18674100	-1.67154500	-1.59051800
C	-3.50996000	-0.13089100	-0.81048500
H	-4.30462700	-0.34374300	-1.54362300
H	-4.61855600	1.46829000	0.16954900
C	-0.00055000	-1.44798000	0.07572700
H	-0.04638600	-2.16458300	-0.77387000
C	1.28195800	-0.63252800	-0.02257100
C	2.31203900	-0.77824500	0.93012700
C	1.47596000	0.27946300	-1.08479500
C	3.50540600	-0.04034800	0.82604400
H	2.17780600	-1.48447700	1.76678200
C	2.66570100	1.01664200	-1.19315300
H	0.67776100	0.41491200	-1.83315800
C	3.68652200	0.85948500	-0.23713000
H	4.29641800	-0.16992000	1.58221800
H	2.79798900	1.72138100	-2.02996400
H	4.61944600	1.43938100	-0.32119600
H	0.04444800	-2.07164100	0.99579700

8. References

(1) Dan, X.; Yang, Q.; Xing, L.; Tang, Y.; Wang, W.; Cai, Y. Heterogeneous Metallaphotocatalytic C(Sp ^{2})–C(Sp ^{3}) Cross-Coupling Reactions with Integrated Bipyridyl-Ni(II)-Carbon Nitride. *Org. Lett.* **2023**, *25* (22), 4124–4129. https://doi.org/10.1021/acs.orglett.3c01384.

(2) St. Denis, J. D.; Scully, C. C. G.; Lee, C. F.; Yudin, A. K. Development of the Direct Suzuki–Miyaura Cross-Coupling of Primary *B* -Alkyl MIDA-Boronates and Aryl Bromides. *Org. Lett.* **2014**, *16* (5), 1338–1341. https://doi.org/10.1021/ol500057a.

(3) Cao, Q.; Howard, J. L.; Wheatley, E.; Browne, D. L. Mechanochemical Activation of Zinc and Application to Negishi Cross-Coupling. *Angew. Chem. Int. Ed.* **2018**, *57* (35), 11339–11343. https://doi.org/10.1002/anie.201806480.

(4) Cheng, Y.; Dong, W.; Wang, L.; Parthasarathy, K.; Bolm, C. Iron-Catalyzed Hetero-Cross-Dehydrogenative Coupling Reactions of Sulfoximines with Diarylmethanes: A New Route to *N* -Alkylated Sulfoximines. *Org. Lett.* **2014**, *16* (7), 2000–2002. https://doi.org/10.1021/ol500573f.

(5) Forster, F.; Metsänen, T. T.; Irran, E.; Hrobárik, P.; Oestreich, M. Cooperative Al–H Bond Activation in DIBAL-H: Catalytic Generation of an Alumenium-Ion-Like Lewis Acid for Hydrodefluorinative Friedel–Crafts Alkylation. *J. Am. Chem. Soc.* **2017**, *139* (45), 16334–16342. https://doi.org/10.1021/jacs.7b09444.

(6) Yang, K.; Lu, J.; Li, L.; Luo, S.; Fu, N. Electrophotochemical Metal-Catalyzed Decarboxylative Coupling of Aliphatic Carboxylic Acids. *Chem. – Eur. J.* **2022**, *28* (70), e202202370. https://doi.org/10.1002/chem.202202370.

(7) Larsen, M. A.; Wilson, C. V.; Hartwig, J. F. Iridium-Catalyzed Borylation of Primary Benzylic C–H Bonds without a Directing Group: Scope, Mechanism, and Origins of Selectivity. *J. Am. Chem. Soc.* **2015**, *137* (26), 8633–8643. https://doi.org/10.1021/jacs.5b04899.

(8) Wang, Z.-Y.; Ma, B.; Xu, H.; Wang, X.; Zhang, X.; Dai, H.-X. Arylketones as Aryl Donors in Palladium-Catalyzed Suzuki–Miyaura Couplings. *Org. Lett.* **2021**, *23* (21), 8291–8295. https://doi.org/10.1021/acs.orglett.1c03048.

(9) Lyons, D. J. M.; Dinh, A. H.; Ton, N. N. H.; Crocker, R. D.; Mai, B. K.; Nguyen, T. V. Ring Contraction of Tropylium Ions into Benzenoid Derivatives. *Org. Lett.* **2022**, *24* (13), 2520–2525. https://doi.org/10.1021/acs.orglett.2c00663.

(10) He, T.; Klare, H. F. T.; Oestreich, M. Catalytically Generated Meerwein's Salt-Type Oxonium Ions for Friedel–Crafts C(Sp2)–H Methylation with Methanol. *J. Am. Chem. Soc.* **2023**, *145* (6), 3795–3801. https://doi.org/10.1021/jacs.2c13341.

(11) Dan, X.; Yang, Q.; Xing, L.; Tang, Y.; Wang, W.; Cai, Y. Heterogeneous Metallaphotocatalytic C(Sp2)-C(Sp3) Cross-Coupling Reactions with Integrated Bipyridyl-Ni(II)-Carbon Nitride. *Org. Lett.* **2023**, *25* (22), 4124–4129. https://doi.org/10.1021/acs.orglett.3c01384.

(12) Xiong, Y.; Wu, X. Deoxygenative Coupling of Alcohols with Aromatic Nitriles Enabled by Direct Visible Light Excitation. *Org. Biomol. Chem.* **2023**, *21* (47), 9316–9320. https://doi.org/10.1039/D3OB01676E.

(13) Kong, D.; Moon, P. J.; Qian, W.; Lundgren, R. J. Expanding the Limit of Pd-Catalyzed Decarboxylative Benzylations. *Chem. Commun.* **2018**, *54* (50), 6835–6838. https://doi.org/10.1039/C8CC02380H.

(14) *Extremely Efficient Cross-Coupling of Benzylic Halides with Aryltitanium Tris(isopropoxide) Catalyzed by Low Loadings of a Simple Palladium(II) Acetate/Tris(p-tolyl)phosphine System - Chen - 2010 - Advanced Synthesis & Catalysis - Wiley Online Library*. https://onlinelibrary.wiley.com/doi/10.1002/adsc.201000311 (accessed 2024-07-17).

(15) Zhu, M.; Qiu, Z.; Zhang, Y.; Du, H.; Li, J.; Zou, D.; Wu, Y.; Wu, Y. Synthesis of (*E*)-Prop-1-Ene-1,3-Diyldibenzene Derivatives via Direct Decarboxylative Coupling of α,β-Unsaturated Carboxylic Acids with Benzyl Boronic Acid Pinacol Ester. *Tetrahedron Lett.* **2017**, *58* (23), 2255–2257. https://doi.org/10.1016/j.tetlet.2017.04.084.

(16) *Application of Hantzsch Ester and Meyer Nitrile in Radical Alkynylation Reactions | Organic Letters*. https://pubs-1acs-1org-101a140gj07a0.han.amu.edu.pl/doi/10.1021/acs.orglett.8b03050#_i9 (accessed 2025-06-26).

(17) Gaussian 16, Revision C.01, M. J. Frisch, G. W. Trucks, H. B. Schlegel, G. E. Scuseria, M. A. Robb, J. R. Cheeseman, G. Scalmani, V. Barone, G. A. Petersson, H. Nakatsuji, X. Li, M. Cari-Cato, A. V. Marenich, J. Bloino, B. G. Janesko, R. Gomperts, B. Mennucci, H. P. Hratchian, J. V. Ortiz, A. F. Izmaylov, J. L. Sonnenberg, D. Williams-Young, F. Ding, F. Lipparini, F. Egidi, J. Goings, B. Peng, A. Petrone, T. Henderson, D. Ranasinghe, V. G. Zakrzewski, J. Gao, N. Rega, G. Zheng, W. Liang, M. Hada, M. Ehara, K. Toyota, R. Fukuda, J. Hasegawa, M. Ishida, T. Nakajima, Y. Honda, O. Kitao, H. Nakai, T. Vreven, K. Throssell, J. A. Montgomery, Jr., J. E. Peralta, F. Ogliaro, M. J. Bearpark, J. J. Heyd, E. N. Brothers, K. N. Kudin, V. N. Staroverov, T. A. Keith, R. Kobayashi, J. Normand, K. Raghavachari, A. P. Rendell, J. C. Burant, S. S. Iyengar, J. Tomasi, M. Cossi, J. M. Millam, M. Klene, C. Adamo, R. Cammi, J. W. Ochterski, R. L. Mar-Tin, K. Morokuma, O. Farkas, J. B. Foresman, and D. J. Fox, Gaussian, Inc., Wallingford CT, 2019.

(18) Weigend, F.; Ahlrichs, R. Balanced Basis Sets of Split Valence, Triple Zeta Valence and Quadruple Zeta Valence Quality for H to Rn: Design and Assessment of Accuracy. *Phys. Chem. Chem. Phys.* **2005**, *7* (18), 3297–3305. https://doi.org/10.1039/B508541A.

(19) Marenich, A. V.; Cramer, C. J.; Truhlar, D. G. Universal Solvation Model Based on Solute Electron Density and on a Continuum Model of the Solvent Defined by the Bulk Dielectric Constant and Atomic Surface Tensions. *J. Phys. Chem. B* **2009**, *113* (18), 6378–6396. https://doi.org/10.1021/jp810292n.

(20) CYLview20; Legault, C. Y., Université de Sherbrooke, 2020 (Http://Www.Cylview.Org).
